# Multiscale footprints reveal the organization of *cis*-regulatory elements

**DOI:** 10.1038/s41586-024-08443-4

**Published:** 2025-01-22

**Authors:** Yan Hu, Max A. Horlbeck, Ruochi Zhang, Sai Ma, Rojesh Shrestha, Vinay K. Kartha, Fabiana M. Duarte, Conrad Hock, Rachel E. Savage, Ajay Labade, Heidi Kletzien, Alia Meliki, Andrew Castillo, Neva C. Durand, Eugenio Mattei, Lauren J. Anderson, Tristan Tay, Andrew S. Earl, Noam Shoresh, Charles B. Epstein, Amy J. Wagers, Jason D. Buenrostro

**Affiliations:** 1 Gene Regulation Observatory, Broad Institute of MIT and Harvard, Cambridge, MA USA; 2 Department of Stem Cell and Regenerative Biology, Harvard University, Cambridge, MA USA; 3 Division of Genetics and Genomics, Boston Children’s Hospital, Boston, MA USA; 4 Eric and Wendy Schmidt Center, Broad Institute of MIT and Harvard, Cambridge, MA USA; 5 Paul F. Glenn Center for the Biology of Aging, Harvard Medical School, Boston, MA USA; 6 Present Address: Department of Genetics and Genomic Sciences, Icahn School of Medicine at Mount Sinai, New York, NY USA

**Keywords:** Epigenomics, Gene regulatory networks, Epigenomics, Machine learning

## Abstract

*Cis*-regulatory elements (CREs) control gene expression and are dynamic in their structure and function, reflecting changes in the composition of diverse effector proteins over time^1^. However, methods for measuring the organization of effector proteins at CREs across the genome are limited, hampering efforts to connect CRE structure to their function in cell fate and disease. Here we developed PRINT, a computational method that identifies footprints of DNA–protein interactions from bulk and single-cell chromatin accessibility data across multiple scales of protein size. Using these multiscale footprints, we created the seq2PRINT framework, which uses deep learning to allow precise inference of transcription factor and nucleosome binding and interprets regulatory logic at CREs. Applying seq2PRINT to single-cell chromatin accessibility data from human bone marrow, we observe sequential establishment and widening of CREs centred on pioneer factors across haematopoiesis. We further discover age-associated alterations in the structure of CREs in murine haematopoietic stem cells, including widespread reduction of nucleosome footprints and gain of de novo identified Ets composite motifs. Collectively, we establish a method for obtaining rich insights into DNA-binding protein dynamics from chromatin accessibility data, and reveal the architecture of regulatory elements across differentiation and ageing.

## Abstract (web summary)

We developed PRINT, a computational method that identifies footprints of DNA–protein interactions from bulk and single-cell chromatin accessibility data across multiple scales of protein size.

## Main

Through homeostasis, development and disease, cells utilize *cis-*regulatory elements (CREs) to regulate gene expression^1^. CREs integrate the binding of structurally diverse regulatory proteins that dynamically move to recruit or evict cooperating factors^2^ and determine the overall function and potential of cells^3^. One major challenge in functional genomics is to identify the precise genomic locations and dynamics of these regulatory proteins across all cell types, to understand the logic of genetic networks and decipher the function of non-coding genetic variation. This challenge is remarkable for its complexity and scale: in humans, cells use diverse combinations of around 2,000 transcription factors (TFs) to modulate the activity of roughly 1 million candidate CREs (cCREs) that regulate the expression of approximately 30,000 genes^4^. To decode this regulatory complexity, thousands of chromatin immunoprecipitation sequencing (ChIP–seq) experiments were performed across a broad range of regulatory proteins and cellular contexts^4^. However, these efforts are limited in that ChIP-based methods cannot scale to measure the binding of all regulatory proteins across all cellular contexts.

Single-cell assay for transposase-accessible chromatin using sequencing (scATAC–seq)^5–7^ has emerged as a powerful and scalable tool for measuring the accessibility of cCREs across the full cellular diversity of fetal and adult tissues^8^. Because TFs predominantly bind open chromatin^9^, the intersection of TF motifs with accessible regions is often used as a proxy for TF binding^10^. To achieve higher precision, statistical methods use chromatin accessibility to ‘footprint’ protein binding at cCREs by quantifying the protection of DNA from DNase^11^, MNase or Tn5 cleavage^5,12^. However, footprinting methods are limited by the sequence bias of enzymes^13^, focus primarily on TF-scale objects (roughly 20 base pairs (bp)), do not detect a large fraction of TFs^14^ or are not well adapted to single-cell methods. Recent advances in deep learning have been valuable in the investigation of diverse aspects of gene regulation^15,16^, allowing for de novo interpretation and in silico manipulation of the sequence features underlying complex patterns in biological data. Motivated by these advances, we sought to combine the precision of DNA footprinting with the inferential power of deep learning to generate accurate maps of diverse regulatory proteins from scATAC–seq data at high genomic and cell-state resolution.

Here we describe a two-step decoding of cCREs. First, we create a new tool, named PRINT (for ‘protein–regulatory element interactions at nucleotide resolution using transposition’), that corrects for enzymatic sequence bias and defines multiscale footprint representations of cCREs, showing regulatory proteins (for example, TFs and nucleosomes) of diverse size. We then develop seq2PRINT, a deep learning framework that parses the sequence-level organization of multiscale footprints in cCREs. We find that seq2PRINT enables computationally tractable and precise TF binding prediction in both bulk and scATAC–seq. We apply seq2PRINT to scATAC–seq and RNA sequencing (RNA-seq) analysis of human bone marrow cells, and track TF and nucleosome binding dynamics across human haematopoiesis. We find that many cCREs exhibit switching of regulatory TFs through differentiation in a manner not reflected by overall accessibility. Tracking regulatory changes through differentiation, we elucidate a stepwise model of activation of erythroid and lymphoid cCREs. Because epigenetic alterations, including aberrant nucleosome remodelling, are a hallmark of ageing^17^, we examine cCRE changes across ageing in mouse HSCs. We find global alteration of nucleosome positioning within cCREs and identify key age-associated TFs across cCREs. These include both decreased activity of nucleosome-associated TFs, such as Yy1 and Nrf1, and increased binding at de novo motifs representing Ets and Runx family members in a broad range of cobinding configurations. Together, these results show that multiscale footprinting, combined with deep learning sequence models, is a powerful method for prediction of TF binding and elucidation of the structural dynamics of cCREs at genome scale.

## Identification of multiscale footprints

**Fig. 1:**
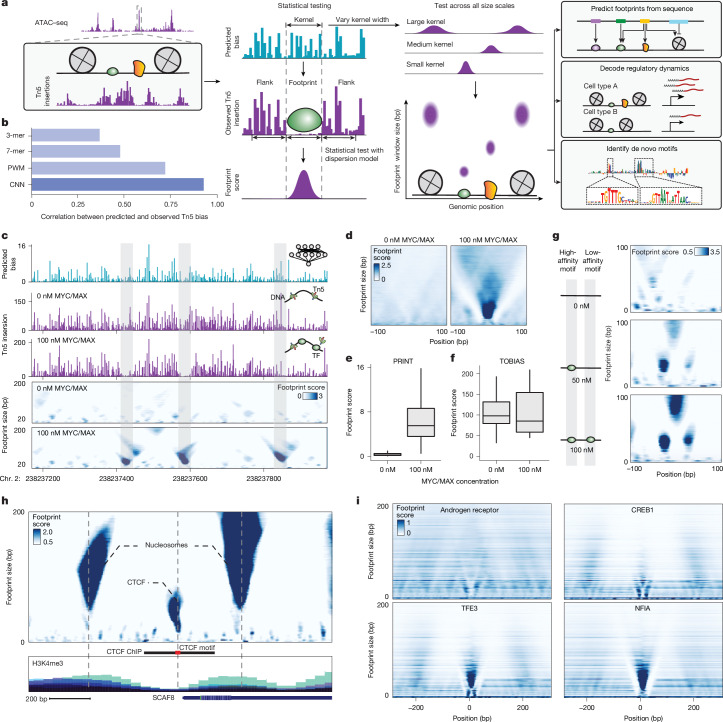
Multiscale footprinting detects DNA–protein interactions across spatial scales. **a**, Overview of the multiscale footprinting workflow. **b**, Bar plot comparing the performance of different Tn5 bias correction models. **c**, Predicted Tn5 bias, observed Tn5 insertion and multiscale footprints on BAC DNA incubated with either 0 nM (top) or 100 nM (bottom) MYC/MAX at example region chr. 2:238237173–238237972. **d**, Aggregate multiscale footprints on BAC DNA incubated with either 0 or 100 nM MYC/MAX at MYC/MAX motif sites. **e**,**f**, Box plots of footprint score at MYC/MAX motif sites with either 0 or 100 nM MYC/MAX, showing results for PRINT (**e**, *n* = 275 positions) and TOBIAS^18^ (**f**, *n* = 158 positions). Boxes show first, second and third quartiles, whiskers show the furthest point falling within the first quartile − 1.5× interquartile range (IQR) or third quartile + 1.5× IQR. **g**, Multiscale footprints at two adjacent MYC/MAX motif sites with 0 nM (top), 50 nM (middle) and 100 nM (bottom) MYC/MAX. **h**, Multiscale footprints in the cCRE region chr. 6:154732971–154733770 in HepG2. Bottom tracks are layered ENCODE histone ChIP signals. **i**, Aggregate multiscale footprints for example TFs including AR, CREB1, TFE3 and NFIA. CNN, convolutional neural network.

**Extended Data Fig. 1:**
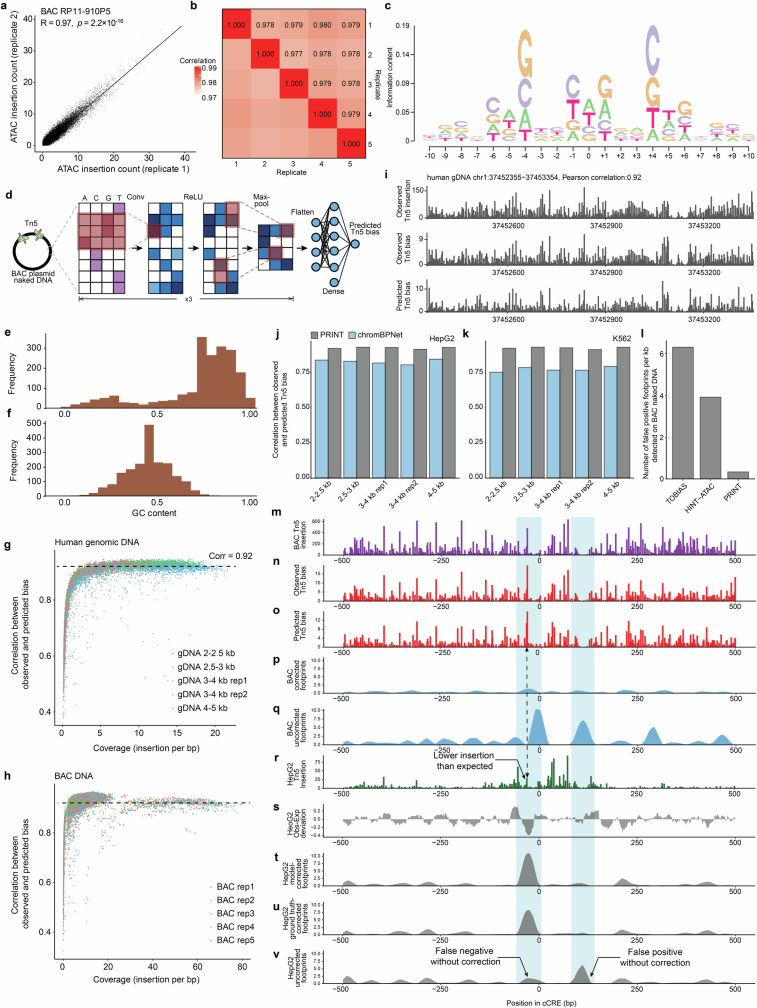
Tn5 bias modelling and footprinting. **a**, Scatter plot comparing single nucleotide observed Tn5 insertion bias on BAC RP11-910P5 from replicate 1 and 2. Pearson’s correlation coefficient = 0.97, p-value = 2.2 × 10^−16^. **b**, Heatmap showing Pearson correlation coefficient of observed Tn5 on all BACs among 5 replicates. **c**, Motif logo of Tn5 sequence bias. **d**, Schematic illustration of the Tn5 bias prediction model. **e**, Histogram of local GC-content in a +/− 10 bp window for top 2000 genomic positions where the neural network Tn5 bias model achieved the highest improvement in prediction error compared to the PWM bias model. **f**, Histogram of local GC-content in a +/− 10 bp window for bottom 2000 genomic positions where the neural network Tn5 bias model achieved the least improvement in prediction error compared to the PWM bias model. **g**-**h**, Scatter plot showing the performance of the Tn5 bias model (trained on BAC data) on human genomic DNA (**g**) and BAC naked DNA (**h**) at different sequencing coverage. Performance is evaluated using the Pearson correlation coefficient between predicted and observed Tn5 bias. **i**, Tracks showing observed Tn5 insertion and Tn5 bias on human genomic DNA, as well as the Tn5 bias predicted by the neural network model trained on BAC data. Observed Tn5 bias is calculated by dividing the Tn5 insertion at each position by the local average Tn5 insertion within a +/− 50 bp window. Showing example region at chr1:37452355-37453354. **j**-**k**, Bar plots showing correlation between observed Tn5 bias and Tn5 bias predicted by ChromBPNet and PRINT, respectively. The ChromBPNet model was trained on (**j**) HepG2 and (**k**) K562 ATAC data^66^. **l**, Bar plot showing the frequency of calling false positive footprints by previous ATAC-footprinting methods and PRINT. **m**-**v**, Testing our footprinting framework in an example cCRE region. **m**-**q**, Results for BAC naked DNA. **m**, Observed raw Tn5 insertion counts. **n**, Observed Tn5 bias. **o**, Tn5 bias predicted by the convolutional neural network. **p**, Footprint scores with bias correction. **q**, Footprint scores without bias correction. **r-v**, Results for HepG2 chromatin ATAC-seq. **r**, Observed raw Tn5 insertion counts. **s**, Observed-expected deviation of centre / (centre + flank) insertion ratio. **t**, Footprint scores with model-based bias correction. **u**, Footprint scores with bias correction using ground truth bias in **n**. **v**, Footprint scores without bias correction.

We developed PRINT, a computational approach to detect footprints of DNA-binding proteins of diverse size from bulk or single-cell ATAC–seq data (Fig. 1a). To first overcome the sequence bias of Tn5 transposase^5^, which can significantly confound footprint detection, we trained a convolutional neural network on Tn5 insertion data on deproteinized DNA from bacterial artificial chromosomes (BACs) (Extended Data Fig. 1a–d and Supplementary Table 1). This model significantly outperformed *k*-mer and position weight matrix (PWM) models (*R* = 0.94; Fig. 1b), particularly in regions of high GC content, performed similarly well on Tn5 insertion data from extracted human genomic DNA data (*R* = 0.92) and outperformed Tn5 bias correction by ChromBPNet^15^ (Extended Data Fig. 1e–k). We provide precalculated Tn5 bias prediction for the human genome and common model organisms, as well as a pretrained deep learning model as a resource to the field (‘Data availability’).

**Extended Data Fig. 2:**
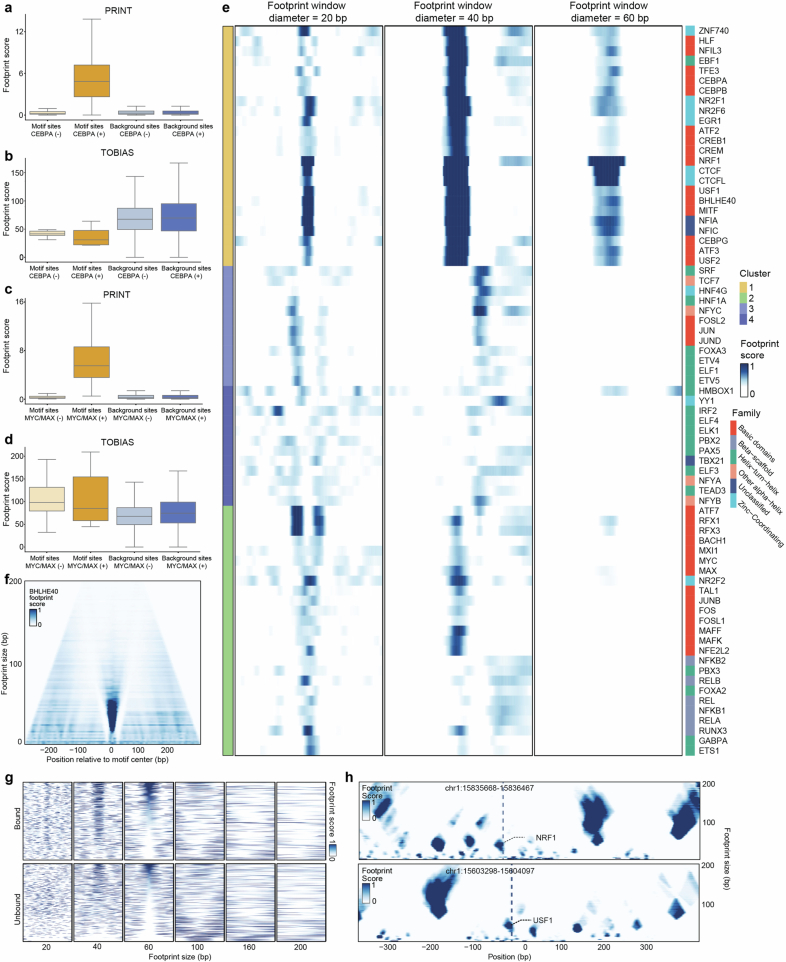
Multiscale footprinting detects binding of diverse TFs. **a-d**, Box plots showing footprint scores at motif sites or background (non-motif) sites with 0 nM or 100 nM TFs. Showing results for PRINT (**a**, **c**) and TOBIAS (**b**, **d**). Both CEBPA (**a**-**b**) and MYC/MAX (**c**-**d**) were tested for footprinting. Number of single base pair positions included were (**a**) n = 207880, (**b**) n = 104895, (**c**) n = 207880, and (**d**) n = 104896. Boxes show 1^st^, 2^nd^, and 3^rd^ quartile. Whiskers show the farthest point falling within the 1^st^ quartile − 1.5 interquartile range (IQR) or 3^rd^ quartile + 1.5 IQR. **e**, Heatmap showing clustering of multiscale aggregate footprints of different TFs. Each row is the multiscale aggregate footprints of a specific TF. Left colour bar shows TF cluster membership. Right colour bar shows the TF family each TF belongs to^87^. Showing footprint results with footprint window diameter of 20 bp, 40 bp, and 60 bp, respectively. **f**, Aggregate multiscale footprints of BHLHE40 in CD8 + T cells. **g**, Footprint at different scales at bound and unbound USF1 motif sites as defined by ChIP-exo data. **h**, Multiscale footprints at example NRF1 and USF1 binding sites indicated by ChIP-exo.

PRINT identifies footprints across diverse scales of protein size with high sensitivity and specificity. We developed a statistical approach that quantifies the significance of depletion of observed Tn5 insertions, relative to an estimated background dispersion at a given position, to yield a footprint score (Methods). This approach reduces false-positive detection on deproteinized DNA by an order of magnitude, in contrast to previous footprinting methods^18^ (Extended Data Fig. 1l–v). Inspired by earlier methods using MNase^19^ or ATAC–seq^5,20,21^ fragment size to infer DNA-bound proteins of varying size, we next computed footprint scores across window sizes ranging 4–200 bp. We validated this method in vitro on deproteinized DNA incubated with purified MYC/MAX or CEBPA. Strong footprints were detected at TF motif sites only in the presence of purified TF with very low background signal, whereas a well-established ATAC–seq footprinting method^18^ did not detect any distinction between foreground and background (Fig. 1c–f and Extended Data Fig. 2a–d). Furthermore, we identified an increase in footprints at low-affinity sites at higher concentrations (100 versus 50 nM) of MYC/MAX, suggesting that footprint scores are sensitive to TF occupancy at a given site (Fig. 1g).

We found that PRINT can detect footprints in mammalian cells. We observed distinct footprint patterns corresponding to nucleosomes and specific TFs (Fig. 1h–i). TF binding patterns could be clustered into four representative categories (Extended Data Fig. 2e). Consistent with previous studies using DNase I^14^, we found that footprint strength varied among TFs, including some that do not leave detectable footprints, potentially due to weak or transient binding on DNA. Footprints can also be detected for repressor TFs (Extended Data Fig. 2f). We validated footprints by benchmarking against data from ChIP with exonucleases (ChIP–exo), finding agreement at TF-bound sites and possible false negatives of ChIP–exo^22^ (Extended Data Fig. 2g–h). Taken together, our results show that multiscale footprinting with PRINT can robustly detect many distinct DNA-binding proteins.

## A DNA sequence model for footprints

**Fig. 2:**
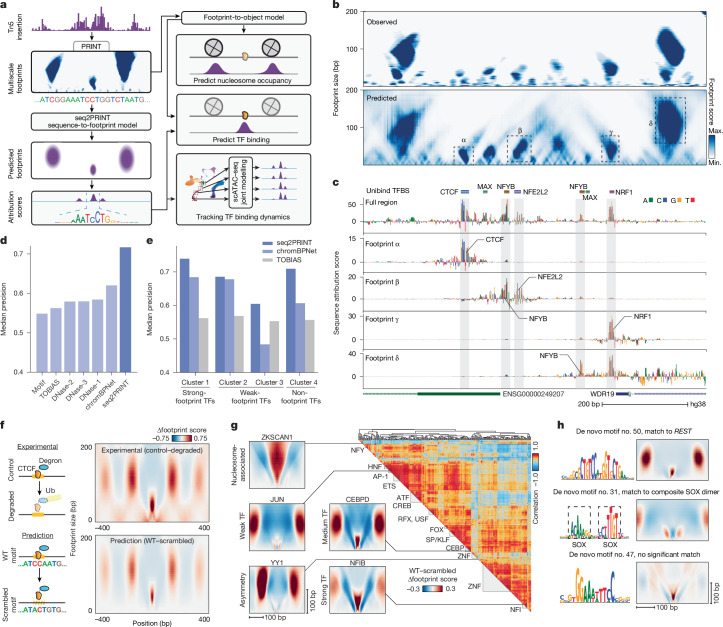
Decoding the genomic syntax of cCRE organization. **a**, Schematic of footprint-to-object prediction and seq2PRINT models and their applications. **b**, Observed (top) and predicted multiscale footprints (bottom) in example region chr. 4:39181940–39182739. **c**, Seq2PRINT sequence attribution scores in the region depicted in **b**, showing attribution scores calculated with respect to multiscale footprints in the whole region (track 1) or specific footprints (tracks 2–5). **d**, Bar plot showing median precision of TF binding prediction by different methods. **e**, Bar plot showing median precision of TF binding prediction by seq2PRINT, ChromBPNet and TOBIAS for different TF clusters, as defined in Extended Data Fig. 2e. **f**, Left, schematic representation of experimental depletion of degron-tagged CTCF and in silico disruption of CTCF binding sites. Right, observed changes in multiscale footprints at CTCF motif sites following experimental CTCF depletion, and seq2PRINT-predicted multiscale footprint changes following in silico CTCF motif disruption. **g**, Left, seq2PRINT-predicted changes in multiscale footprints following in silico ablation of motif sites of example TFs. Right, heatmap showing clustering of TFs based on seq2PRINT-predicted changes following in silico motif ablation. **h**, Representative de novo motifs identified by TF motif discovery from sequence attribution scores (TF-MoDISco) from seq2PRINT-predicted sites, and corresponding predicted changes in multiscale footprints following in silico motif ablation (colour scale as in **g**). Max., maximum; min., minimum.

**Extended Data Fig. 3:**
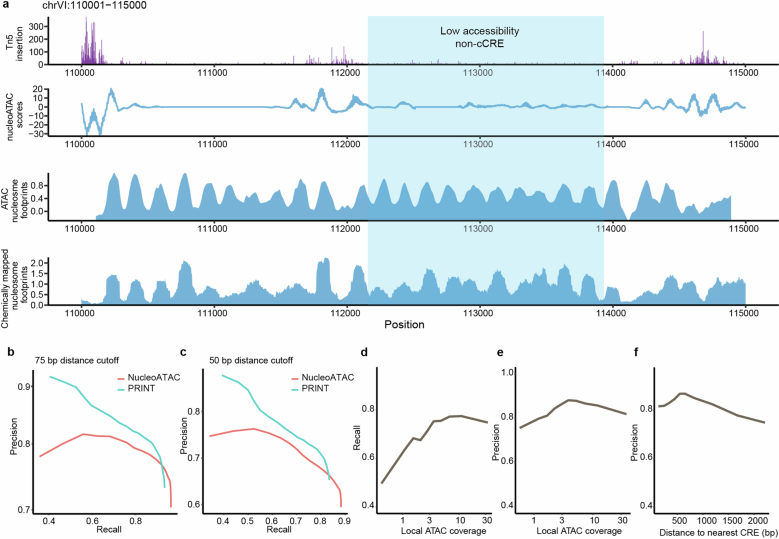
Footprint-to-nucleosome prediction. **a**, Tracks showing observed Tn5 insertion, nucleosome occupancy predicted by NucleoATAC and our footprint-to-nucleosome model, as well as ground truth nucleosome occupancy measured by chemical mapping. **b**, **c**, Precision-recall curve of NucleoATAC and PRINT for nucleosome mapping. Predicted nucleosome centres are considered to be a true positive prediction if it is within 75 bp (**c**) or 50 bp (**d**) from the nearest ground truth nucleosome centre. **d**-**f**, Line plots showing the relationship between model performance and local coverage or distance to nearest cCRE.

We next sought to use multiscale footprints to predict the binding of specific proteins to DNA. We designed models that predict the binding of TFs and nucleosomes (Fig. 2a, Methods and Supplementary Notes). The nucleosome model uses multiscale footprints as input to predict nucleosome summits mapped by nucleosome chemical mapping data^23^; we found that this model outperformed previous work^20^ (Extended Data Fig. 3a–f).

**Extended Data Fig. 4:**
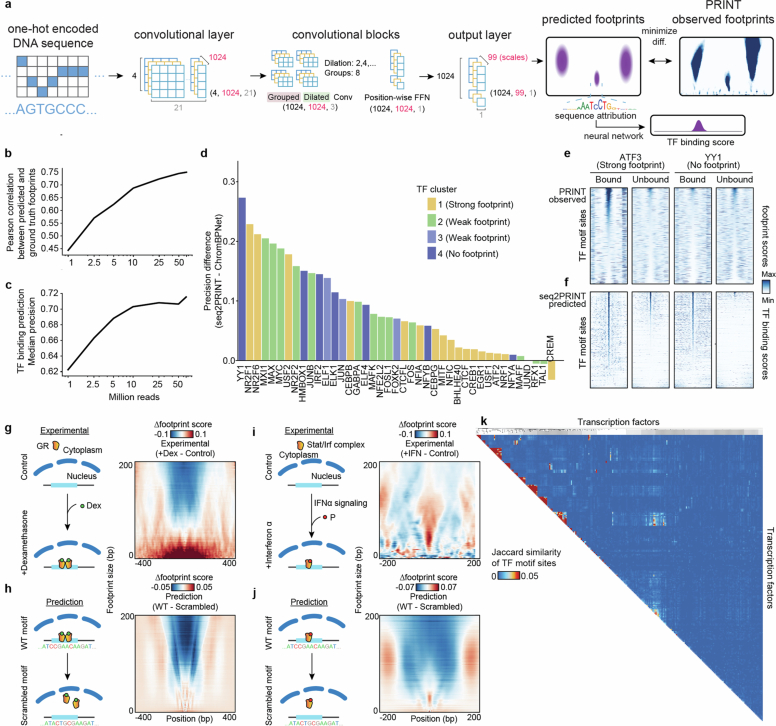
The seq2PRINT framework. **a**, Schematic illustration of the seq2PRINT model. **b**-**c**, Down-sampling test for seq2PRINT. Showing (**b**) Pearson correlation coefficient between predicted and ground truth multiscale footprints and (**c**) median precision of TF binding prediction at top 10% predicted binding sites. Here, ground truth footprints were defined as observed footprints without any down-sampling. **d**, Waterfall plot showing precision improvement of seq2PRINT over ChromBPNet for individual TFs. **e**, Observed footprints (40 bp scale) at bound and unbound motif sites for ATF3 and YY1. Bound and unbound motif sites are defined by overlap with ChIP-seq peaks. **f**, TF binding scores predicted by seq2PRINT at bound and unbound motif sites for ATF3 and YY1. **g**, Observed changes in multiscale footprints at GR binding sites after dexamethasone treatment to induce relocalization of GR into the nucleus and binding to DNA^24^. **h**, seq2PRINT-predicted differences in multiscale footprints at GR motif sites comparing WT GR motif and in silico disruption of GR motif. (Pearson correlation=0.73 with observed changes). **i**, Observed changes in multiscale footprints at Stat2 binding sites in B cells after IFN treatment^25^. **j**, seq2PRINT-predicted differences in multiscale footprints at Stat2 binding sites comparing WT Stat2 motif and in silico disruption of Stat2 motif. (Pearson correlation=0.23 with observed changes). **k**, Heatmap showing clustering of TFs based on Jaccard similarity of motif matches. Rows and columns are individual TFs.

Building on recent advances in deep learning^15,16^, we created a model that uses DNA sequence to predict multiscale footprints (seq2PRINT; Fig. 2a and Extended Data Fig. 4a). The model was able to predict both nucleosome and TF footprints using local DNA sequence as the sole input (Fig. 2b). We observed an overall correlation of 0.75 between predicted and observed multiscale footprints in ATAC–seq data from HepG2 cells that was robust to subsampling of read depth (Extended Data Fig. 4b).

We extracted the key sequence features learned by seq2PRINT, and found that the resulting basewise DNA sequence attribution scores enable dissection of the TF binding architecture within a cCRE. In an example locus, attribution scores calculated with respect to the whole cCRE highlighted short sequences overlapping with TF motif positions across the region, whereas calculation of scores for specific footprint objects highlighted specific motifs (Fig. 2b,c). For footprints α and γ, the sequence model identified the motif underlying that footprint, whereas for footprint β both the NFE2L2 motif underneath the footprint and a neighbouring NFYB motif were detected, indicating potential binding coordination between nearby TFs. Footprint δ probably represents a nucleosome and is predicted by nearby TF NRF1 and NFYB motifs, showing even longer-range dependencies and revealing the factors most associated with nucleosome positioning. The latter two examples further suggested that some TFs lacking a strong footprint (for example, NFYB) can be detected by seq2PRINT, probably due to effects on neighbouring elements, and that this approach could be used to model interactions between DNA-binding proteins within a cCRE.

seq2PRINT can be used to predict the genome-wide binding of TFs. We used the sequence attribution scores from seq2PRINT to generate a TF binding score trained to predict ChIP–seq data (Methods). The TF binding score was able to predict TF binding with high precision and outperformed previous methods^15,18^ (Fig. 2d). We found that we were even able to predict TFs with weak or no direct footprint for which other methods demonstrated particularly low performance (Fig. 2e, Extended Data Figs. 2e and 4c–f and Supplementary Table 2). To investigate how the model detected these TFs, we simulated the effects of loss of TF binding by scrambling their motif sequences. The model predicted changes to nearby footprints across scales such as nucleosomes, which correlated well with experimentally determined footprint changes including degron-induced CTCF depletion^4^ (aggregate *R* = 0.93, median per-locus *R* = 0.66; Fig. 2f). Similar results were obtained with dexamethasone-induced glucocorticoid receptor relocation and DNA binding^24^, as well as with IFN-induced Stat2 binding to DNA^25^ (Extended Data Fig. 4g–j). We applied this approach broadly and identified TFs with strong effects on nucleosomes flanking the TF (for example, JUN and YY1) or at the same position of the TF (for example, ZKSCAN1) (Fig. 2g). The predicted changes in footprint score were highly similar among TF families, in a manner largely independent of binding site similarity (Extended Data Fig. 4k).

seq2PRINT attribution scores identify key DNA sequence patterns predictive of footprints, enabling the identification of motifs de novo. Using the trained model, we clustered and aligned local sequence attribution scores and identified 106 de novo motifs. These de novo motifs recovered known motifs in an unbiased fashion, as well as composite motifs such as dimers of SOX (Fig. 2h). Several de novo motifs were associated with strong TF or nucleosomal footprints despite no significant match to a known motif database^26^ (Fig. 2h, bottom). We provide the full list of de novo motifs and their associated multiscale footprints discovered in this work in Supplementary Data 1 and 2.

## cCREs restructure across haematopoiesis

**Fig. 3:**
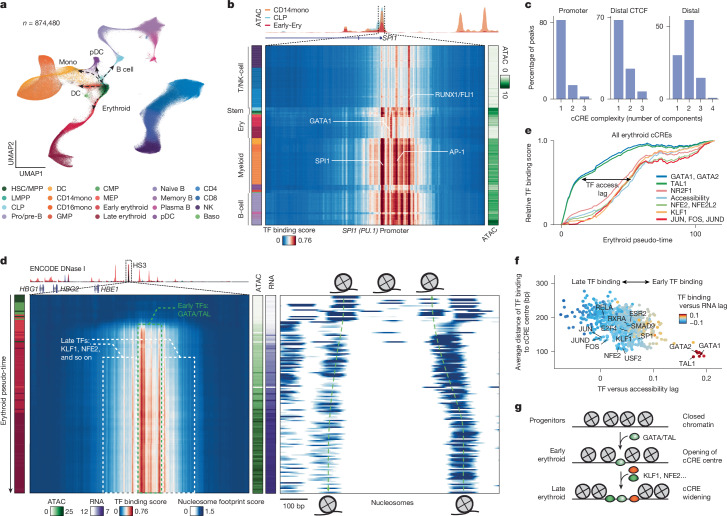
Emerging intra-cCRE dynamics in human haematopoiesis. **a**, Uniform manifold approximation and projection (UMAP) of the human bone marrow SHARE–seq dataset. **b**, Top, ATAC–seq signal surrounding the highlighted *SPI1* promoter region chr. 11:47378111–47378911. Bottom, TF binding scores derived from seq2PRINT in the same *SPI1* promoter region across 1,000 pseudo-bulks. Each row represents a pseudo-bulk and each column a single base-pair position. Left-hand colour legend for cell types as in **a**; right-hand colour legend, accessibility of the promoter region in each pseudo-bulk. **c**, Percentage of all cCREs with the given complexity of TF binding modes. Individual cCRE binding patterns were decomposed into principal components, and complexity was defined as the number of components needed to explain 98% of variance. **d**, Left, TF binding scores derived from seq2PRINT in the locus control region HS3 enhancer region at chr. 11:5284362-5285162 across erythroid differentiation. Rows represent pseudo-bulks ordered by pseudo-time. Colour legend as in **a**. Middle, colour bars represent ATAC–seq signal in the same region, and RNA level of *HBB*. Right, nucleosome footprint scores (100 bp scale) in the same region and pseudo-bulks. **e**, Line plot showing TF binding scores of representative TFs within erythroid cCREs across the pseudo-time of erythroid differentiation. **f**, Scatter plot comparing the timing of TF binding and relative position of TF binding sites with cCRE centres. *X* axis, time lag between TF binding and gain of cCRE accessibility; *y* axis, average distance of TF binding site to cCRE centres. **g**, Schematic illustration showing the sequential binding of TFs and widening of cCREs during differentiation. HSC/MPP, haematopoietic stem cells/multipotent progenitors; LMPP, lymphoid-primed multipotent progenitors; CLP, common lymphoid progenitors; pro/pre-B, progenitor and precursor B cells; DC, dendritic cells; CD14mono, CD14 monocytes; CD16mono, CD16 monocytes; GMP, granulocyte–monocyte progenitors; CMP, common myeloid progenitors; MEP, megakaryocyte–erythroid progenitors; early-Ery, early erythroid cells; late-Ery, late erythroid cells; Naive B, naive B cells; Memory B, memory B cells; Plasma B, plasma B cells; pDC, plasmacytoid dendritic cells; CD4, CD4 T cells; CD8, CD8 T cells; NK, natural killer cells; Baso, basophils.

**Extended Data Fig. 5:**
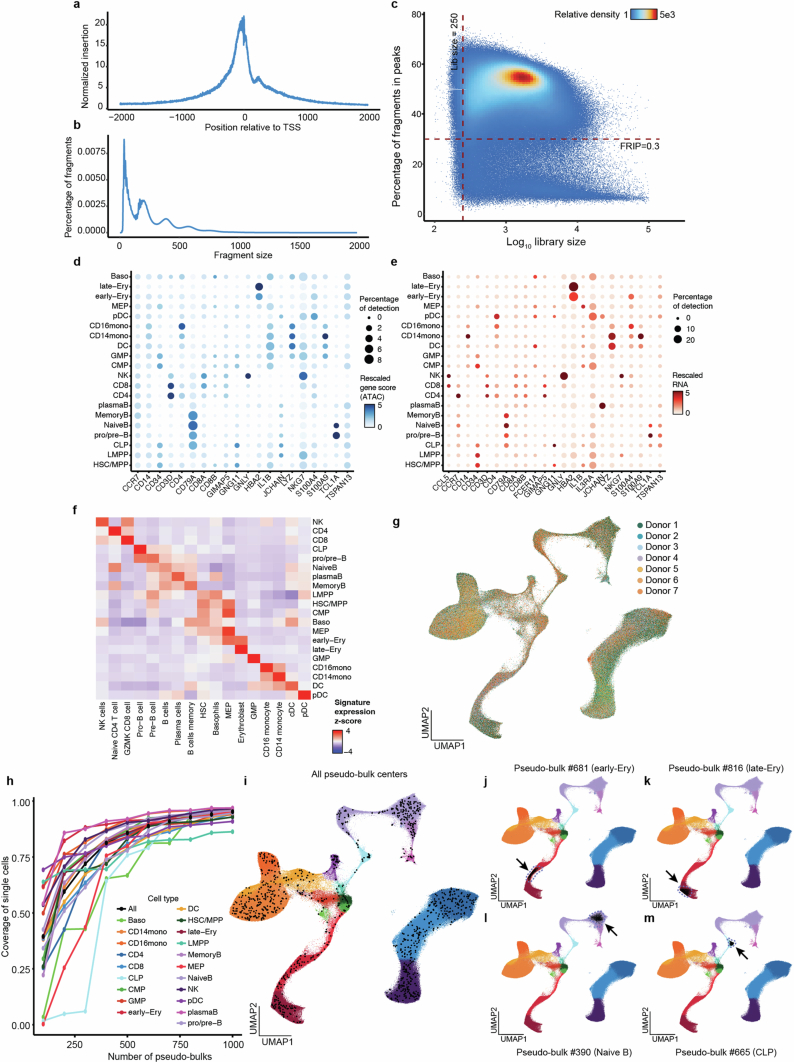
Single cell multi-omic profiling of human bone marrow. **a**, Tn5 insertion enrichment around TSSs. **b**, Fragment size distribution. **c**, Scatter plot showing library size and fraction of reads in peaks (FRIP) of single cells. Dashed lines represent thresholds of FRIP = 0.3 and library size = 250. Density is computed using the kde2d function in the MASS package and rescaled to reflect relative fold of enrichment compared to the cell with the lowest observed density. **d**, Dot plot showing gene scores (ATAC signal within a region around promoter) of marker genes across cell types. **e**, Dot plot showing RNA levels of marker genes across cell types. **f**, Heatmap showing RNA expression z-scores of cell type marker gene signatures in each cluster. Rows represent annotated clusters in our human bone marrow dataset. Columns represent individual cell-type signatures obtained from PanglaoDB^88^ and CellMarker2.0^89^. **g**, UMAP showing donor origin of single cells. **h**, Line plot showing single cell coverage for each cell type as a function of the number of pseudo-bulks. **i**, UMAP showing the positions of pseudo-bulk centres for all 1000 pseudo-bulks we generated. **j-m**, Example pseudo-bulks. Black dots represent member cells within the pseudo-bulk.

**Extended Data Fig. 6:**
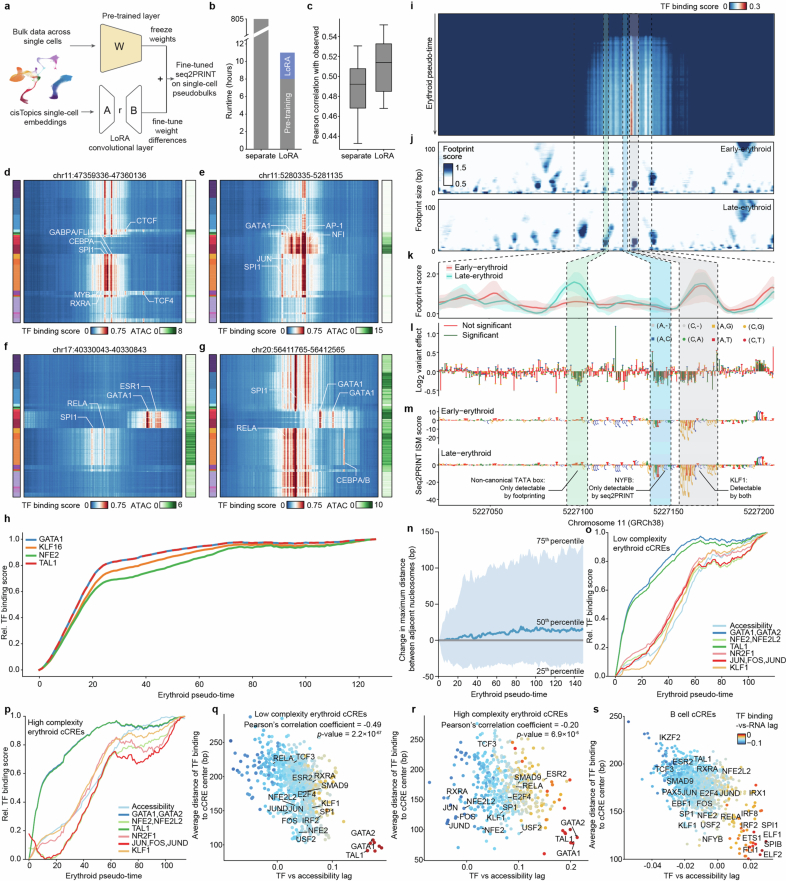
TF and nucleosome dynamics across human hematopoiesis. **a**, Schematic illustration of using LoRA to scale up seq2PRINT training on large numbers of samples (pseudo-bulks). **b**, Bar plot comparing run time of training separate seq2PRINT models on 1000 pseudo-bulks versus using LoRA fine-tuning. **c**, Box plot comparing the Pearson correlation coefficient between predicted and observed footprints by either training separate seq2PRINT models on 1000 pseudo-bulks versus using LoRA fine-tuning. Boxes show first, second, and third quartiles. Whiskers show the farthest point falling within the first quartile − 1.5 IQR or third quartile + 1.5 IQR. **d-g**, Heatmaps of TF binding scores predicted by seq2PRINT in example loci across all 1000 pseudo-bulks. Each row represents a pseudo-bulk and each column represents a single base-pair position. Left: colour legend for cell types with matching colours as Fig. 3a. Right: accessibility of the cCRE region in each pseudo-bulk. **h**, Line plot showing TF binding scores of representative TFs within the HS3 enhancer shown in Fig. 3d across the pseudo-time of erythroid differentiation. **i**-**m**, cCRE reorganization within the *HBB* promoter across erythroid differentiation. **i**, Heatmap of TF binding scores predicted by seq2PRINT in the *HBB* promoter at chr11:5226700-5227499. **j**, Multiscale footprints in the same region as **i** in early- and late-erythroid cells. **k**-**m**, Zoomed-in results in the sub-region chr11:5227022-5227208. **k**, Line plot of footprint scores at 40 bp scale in early- and late- erythroid cells. Showing mean and standard deviation across 7 human donors. **l**, MPRA log_2_ variant effect sizes obtained from ref. ^32^ in the same region as **k**. **m**, Sequence attribution scores in the same region as **k** and **l** generated by seq2PRINT in early- and late-erythroid cells. **n**, Change in the maximum distance between adjacent nucleosomes in erythroid cCREs across differentiation pseudo-time. Showing median and 25-75 percentile range at each pseudo-time. **o**-**r**, Line plot showing TF binding scores of representative TFs within (**o**) low complexity and (**p**) high complexity erythroid cCREs across the pseudo-time of erythroid differentiation. **q**-**r**, Scatter plot comparing the timing of TF binding and relative position of TF binding sites to cCRE centers in (**q**) low complexity and (**r**) high complexity erythroid cCREs. **s**, Scatter plot comparing the timing of TF binding across B lymphocyte differentiation and relative position of TF binding sites to cCRE centres. Horizontal axis: time-lag between TF binding and gain of cCRE accessibility. Vertical axis: average distance of TF binding site to cCRE centers.

Multiscale footprinting and seq2PRINT resolve the dynamics of TF binding across haematopoiesis. We used simultaneous high-throughput ATAC and RNA expression with sequencing (SHARE–seq)^27^ to generate joint scATAC–seq and scRNA-seq datasets for 874,480 bone marrow mononuclear cells from seven human donors (Fig. 3a and Extended Data Fig. 5a–g). To enable footprinting, we merged single cells into 1,000 pseudo-bulks representing all major cell types and developmental transitions (Extended Data Fig. 5h–m and Methods). A central challenge in applying deep learning sequence models is that computational intensity scales poorly with the number of cell types or conditions. To overcome this challenge, we trained a common model across all the data representing 2.2 billion reads, and used low-rank adaptation of large models (LoRA) to fine-tune a sequence model for each pseudo-bulk^28^, achieving around 80-fold improvement in speed and increased prediction accuracy as compared with training separate models for each pseudo-bulk. (Extended Data Fig. 6a–c).

Transcription factor binding predictions show distinct groups of TFs bound at the same cCRE across cell types. At the promoter for *SPI1* (PU.1), a myeloid master regulator, seq2PRINT binding scores were high at SPI1 and AP-1 sites in myeloid cells whereas only strong GATA1 binding was predicted in erythroid cells, consistent with the known regulatory relationships of these TFs^29^ (Fig. 3b). Notably, these distinct TF binding patterns and those at other loci are not distinguishable solely by measurement of the overall accessibility of the promoter (Extended Data Fig. 6d–g). We quantified the complexity of TF binding patterns at each cCRE across the genome using principal component analysis, and found that complex cCREs (more than one component) were highly enriched at distal cCREs relative to promoters (69.8 versus 17.2%; Fig. 3c), highlighting the cell-type-specific utilization of enhancers.

Analysis of TF binding along the erythroid differentiation trajectory demonstrated that the establishment of cCREs occurs sequentially. This is exemplified by the HS3 enhancer within the haemoglobin locus control region^30,31^. In both HSCs and common myeloid progenitors, nucleosomes are unphased and low TF binding is predicted. With the progress of cells along erythroid development, as ordered by pseudo-time, and expression of *HBB* (β-haemoglobin) increases, nucleosome footprints first become phased at the edge of the cCRE with strong TF binding scores at GATA/TAL motif sites. The cCRE then progressively widens, with the addition of KLF1/NFE2 factor binding at the edge (Fig. 3d and Extended Data Fig. 6h). The *HBB* promoter exhibited the same sequential TF binding patterns, and PRINT/seq2PRINT binding predictions at the locus corresponded well with previously published massively parallel reporter assay data^32^ (Extended Data Fig. 6i–m).

We found this pattern of cCREs extending outwards from a central TF across erythroid-associated cCREs genome wide. TF binding scores for GATA and TAL factors increased early in erythroid pseudo-time, whereas appreciable overall cCRE opening, and binding at NFE2, KLF1, NR2F1 and AP-1 factors, occurred later during differentiation (Fig. 3e and Extended Data Fig. 6n). Intriguingly, TFs predicted to bind before the gain in accessibility, and to target gene expression, also bind more closely to the cCRE centre, with later-binding TF motifs localized to flanking regions (Fig. 3f,g and Supplementary Table 3). These observations are largely independent of cCRE complexity (Extended Data Fig. 6o–r). This observation is in line with previous studies showing stereotypical TF motif arrangements within cCREs and enrichment of chromatin remodellers at cCRE edges^5,33,34^. We repeated this analysis on the B cell differentiation trajectory and found the same sequential establishment of cCREs with different central and flanking TFs (Extended Data Fig. 6s). Our findings connect cCRE restructuring and TF binding during in vivo differentiation, suggesting that both core- and flank-binding TFs might contribute differentially to cCRE establishment, expansion and gene expression.

## De novo motifs characterize ageing HSCs

**Fig. 4:**
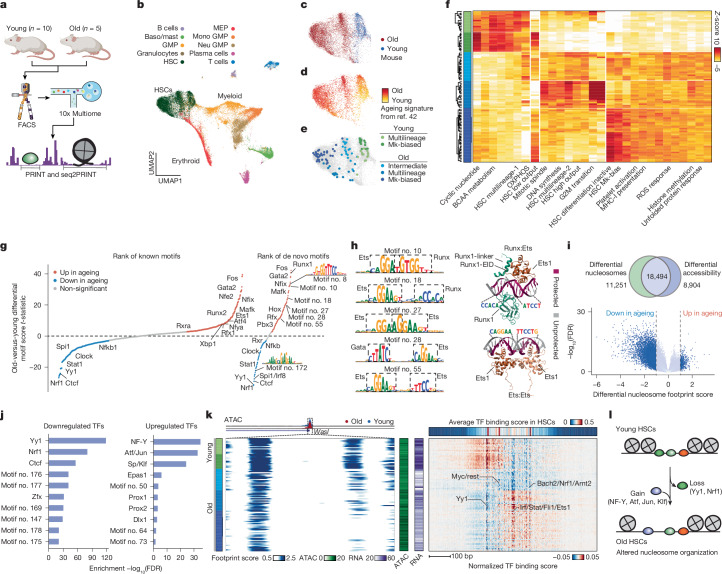
Intra-cCRE dynamics in haematopoietic ageing. **a**, Schematic of data generation and analysis. **b**, UMAP of HSC and progenitor cells. **c**, UMAP showing donor mouse age among HSCs. **d**, UMAP showing activity of the ageing gene signature obtained from ref. ^42^. **e**, UMAP showing five major HSC subpopulations. **f**, Heatmap showing clustering of 100 HSC pseudo-bulks into five major subpopulations using the expression of gene programs. Colour bar represents HSC subpopulation labels of each pseudo-bulk, as in **e**. **g**, Ranking of known (left, obtained from cisBP) and de novo (right, derived from seq2PRINT) TF motifs by old-versus-young differential chromVAR score testing *t*-statistics (two-tailed). Top motif logos with no significant cisBP match are highlighted. **h**, Example composite de novo motifs of Ets homo-/heterodimers, also showing PDB structures of dimers Runx:Ets (PDB-4L0Z) and Ets:Ets (PDB-2NNY). Solvent-inaccessible nucleotide bases are highlighted purple, and core Runx and Ets motifs are shown. **i**, Age-associated nucleosome changes. Top, Venn diagram showing overlap between cCREs with differential nucleosomes and those with differential accessibility during ageing. Bottom, volcano plot of differential nucleosome changes during ageing, pseudocoloured by density. **j**, TF motif enrichment at nucleosome footprints lost during ageing (absolute difference in footprint score greater than 1 and false discovery rate (FDR) less than 0.01). Top ten enriched motifs that are upregulated (right) or downregluated (left) in ageing. **k**, Organization of the *Wasl* promoter at chr. 6:24664695–24665494 in old and young HSCs. Left, nucleosome footprints (100 bp scale); rows represent individual HSC pseudo-bulks and columns represent single bp positions. Left-hand colour bar represents HSC subpopulations as in **e**,**f**; middle colour bars represent chromatin accessibility and *Wasl* RNA levels in pseudo-bulks; right, top colour bar represents average seq2PRINT-predicted TF binding scores across pseudo-bulks. Heatmap shows TF binding scores in each pseudo-bulk, following centring by subtraction of column averages. **l**, Schematic of age-associated cCRE reorganization. Illustrations in **a** created using BioRender (https://biorender.com).

**Extended Data Fig. 7:**
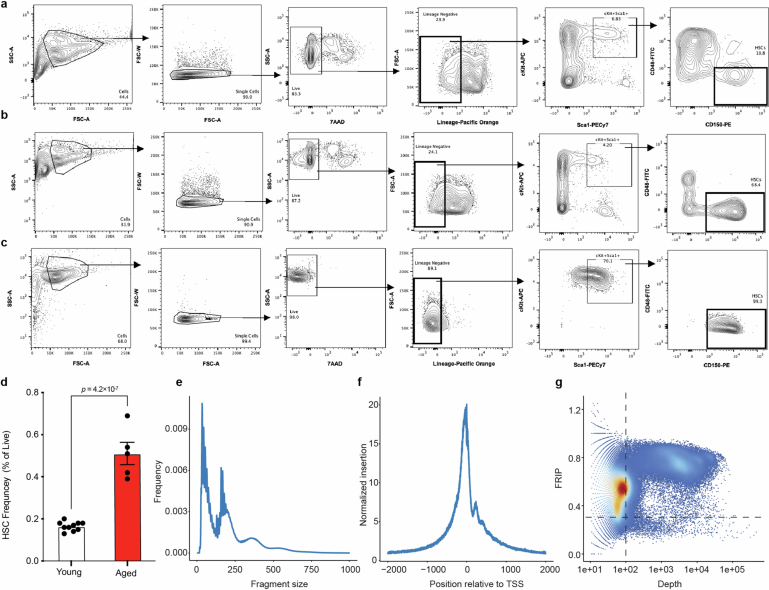
Generation of the mouse hematopoietic stem cell aging dataset. **a-b**, Flow cytometry gating strategy for isolation of haematopoietic progenitor cells (lineage negative, gate bolded; Live Lin^−^) and haematopoietic stem cells (HSCs, gate bolded; Live Lin^−^ Sca1^+^ cKit^+^ CD48^−^ CD150^+^) from the bone marrow (BM) of young (**a**, n = 10) and aged (**b**, n = 5) male C57BL6/J mice. Representative FACS plots shown from one young and one aged mouse. For individual FACS plots from each mouse, see Supplementary Data 3. **c**, Sorting results confirming purity of resorted HSCs was greater than 99%. **d**, Frequency of FACSorted HSCs in young and aged mice (two-tailed t-test; t_13_ = 9.283, p = 4.2 × 10^−7^). The frequency was determined from collecting 100k events for each animal. Displayed is the mean frequency in each group with error bars representing the standard error of mean (SEM). **e-g**, QC metrics of the dataset. **e**, Fragment size distribution. **f**, TSS enrichment. **g**, Depth-FRIP scatter plot.

**Extended Data Fig. 8:**
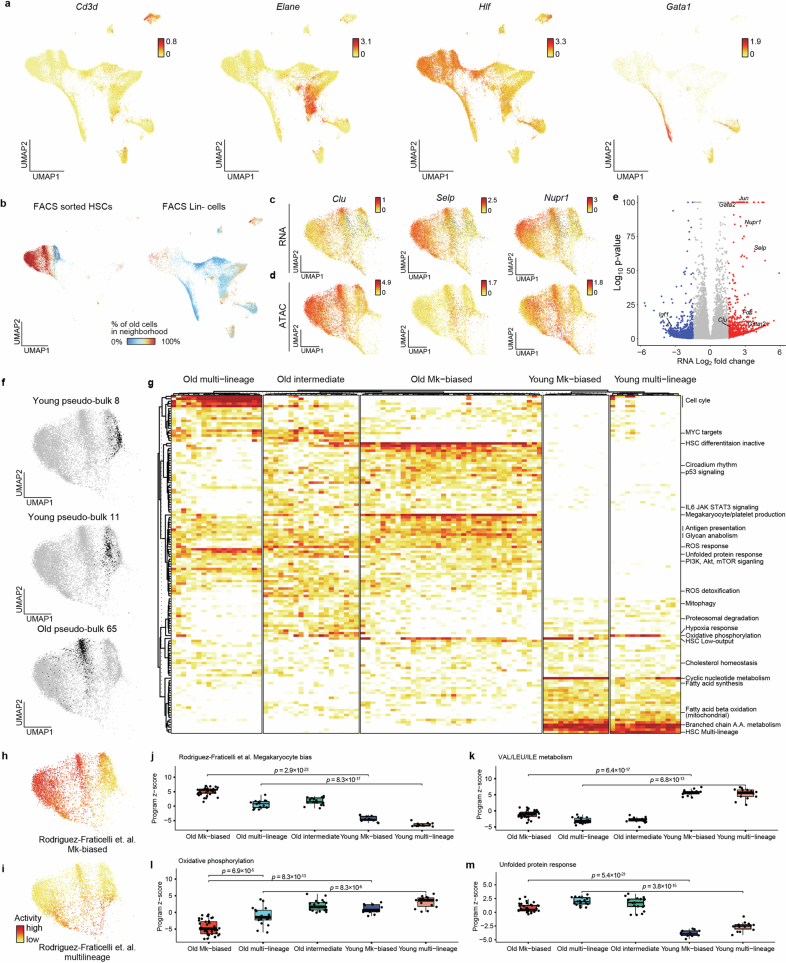
Characterizing age-related changes in mouse HSCs. **a**, UMAP showing gene scores of cell type marker genes *Cd3d*, *Elane*, *Hlf*, and *Gata1*, respectively. **b**, Percentage of old cells in the 100-cell nearest neighbourhood for FACS sorted HSCs (left) or lineage- cells (right). **c-d**, UMAP showing (**c**) RNA and (**d**) ATAC levels of aging marker genes (*Clu*, *Selp*, *Nupr1*) in HSCs. **e**, Volcano plot of differential RNA testing (old-versus-young). *P*-values were calculated using a two-sided Wald test by DESeq2. **f**, UMAP showing example pseudo-bulks. Black dots represent member cells within each pseudo-bulk. **g**, Heatmap showing clustering of pseudo-bulks (columns) and Spectra gene programs (rows). Pseudo-bulks were grouped into 5 major HSC subpopulations as labelled at the top. **h**-**i**, UMAP showing expression of gene signatures for Mk-biased and multi-lineage HSCs. Gene signatures were obtained from ref. ^41^. **j**-**m**, Box plots showing expression of example Spectra gene programs across HSC subpopulations (n = 100 pseudo-bulks). Boxes show first, second, and third quartiles. Whiskers show the farthest point falling within the first quartile − 1.5 IQR or third quartile + 1.5 IQR. *P*-values were derived using a two-sided t-test.

We next sought to use seq2PRINT to investigate changes in cCRE organization during ageing. Biological ageing is a multifactorial process that affects the physiology of a broad range of tissues, including marked changes to function and proliferation of HSCs^35,36^. Because previous studies have shown that ageing is accompanied by widespread epigenetic changes^37,38^, we hypothesized that seq2PRINT would be well suited to detection of differences in TF activity and cCRE structure across ageing. We isolated haematopoietic progenitor cells (Lineage^−^) and HSCs (Lineage^−^Sca1^+^cKit^+^CD48^−^CD150^+^) from the bone marrow of young (11 weeks old, *n* = 10) and aged (24 months old, *n* = 5) male mice by fluorescent activated cell sorting (FACS), and obtained joint ATAC–RNA profiling of 48,225 cells covering 14,640 HSCs and 33,585 haematopoietic progenitor cells using the 10x Multiome platform (Fig. 4a,b and Extended Data Fig. 7a–g). HSCs from both old and young mice clustered separately and matched previously reported ageing signatures and marker genes^39,40^ (Fig. 4c,d, Extended Data Fig. 8a–e and Supplementary Table 4). To further explore HSC heterogeneity, we identified 100 representative cell states among HSCs to generate pseudo-bulks. We identified HSCs states by collection of previously described gene sets covering lineage bias^41^ and ageing^42^, as well as gene programs learned from our data (Methods). Scoring and clustering of HSC pseudo-bulks using these gene sets identified five HSC subpopulations distinguished by age and lineage potential (Fig. 4e,f and Extended Data Fig. 8f–m). These five HSC cell states represent major categories that are consistent with previous findings^41^ and can be further partitioned, suggesting additional axes of age-associated variation (for example, oxidative phosphorylation regulation, unfolded protein response) that may be relevant to the pathogenesis of the aged immune system (Supplementary Table 5).

**Extended Data Fig. 9:**
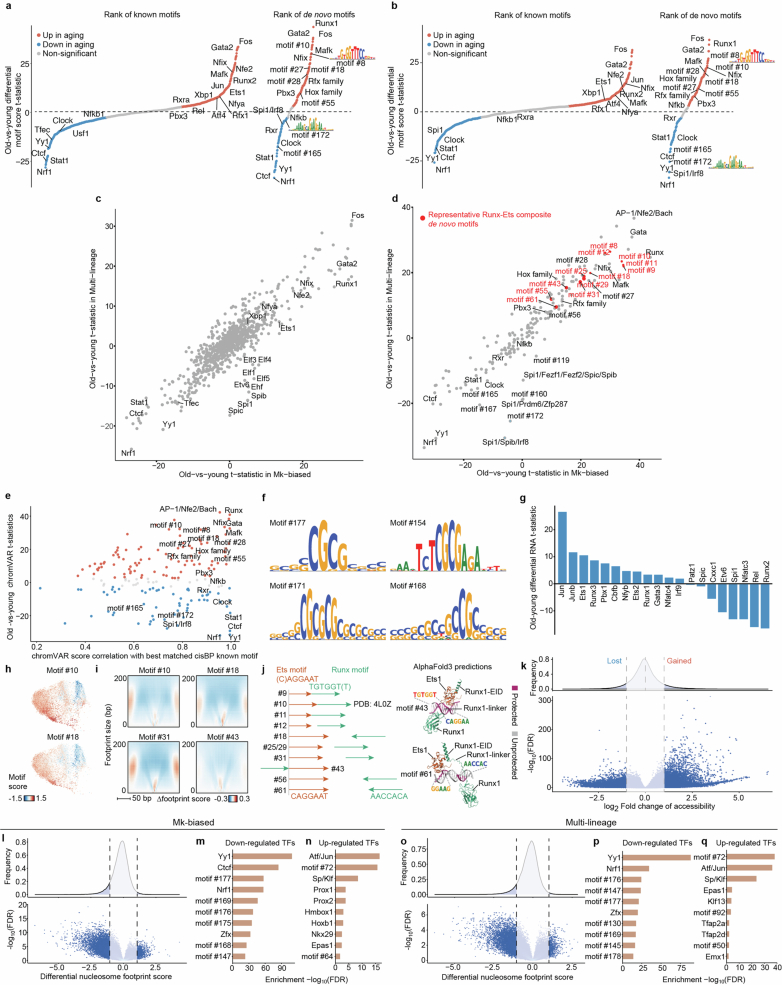
Age-associated cCRE reorganization. **a**-**b**, Ranking of known (left, obtained from cisBP) and de novo (right, derived from seq2PRINT) TF motifs by subpopulation-specific old-vs-young differential t-statistics (two-tailed) of chromVAR scores in (**a**) the Mk-biased subpopulation and (**b**) the multi-lineage subpopulation. **c**-**d**, Scatter plots comparing the results in **a**-**b** between subpopulations for (**c**) cisBP TF motifs, and (**d**) de novo TF motifs learned by seq2PRINT. **e**, Scatter plot comparing old-vs-young differential testing t-statistics (two-tailed) of de novo TF motifs and the correlation of chromVAR scores between each de novo motif and its best matched known cisBP motif. **f**, Example CpG-containing de novo motifs that are down-regulated during aging. **g**, Bar plot showing differential RNA results for partner TFs forming composite motifs with Ets1. **h**, UMAP showing chromVAR motif scores of de novo motif #10 and #18. **i**, seq2PRINT-predicted impact on multiscale footprints by example composite de novo motifs containing Ets1 and Runx1. **j**, Left: different configurations of Runx-Ets composite de novo motifs detected by seq2PRINT in HSCs. Showing the orientation and spacing of the Runx and Ets motifs. Right: AlphaFold3-predicted structures of Ets1 and Runx1 on example composite motifs. Solvent inaccessible nucleotide bases are highlighted purple and the core Runx and Ets motifs are shown. **k**, Age-associated chromatin accessibility changes. Top: density plot of old - young log_2_ fold changes in accessibility for individual cCREs. Bottom: volcano plot with the same horizontal axis values. **l**-**q**, Age associated nucleosome changes in Mk-biased (**l**-**n**) and multi-lineage (**o**-**q**) HSC subpopulations. **l**, **o**, Top: density plot of old - young nucleosome footprint score changes at individual nucleosomes detected by PRINT. Bottom: volcano plot with the same horizontal axis values. **m**, **n**, **p**, **q**, TF motif enrichment at nucleosomes footprints lost during ageing (absolute difference in footprint score > 1 and FDR < 0.01). Top 10 enriched motifs of TFs that are down-regulated (**m**, **p**) or up-regulated (**n**, **q**) in ageing.

We found that cCREs undergo extensive changes to TF binding on ageing. We applied seq2PRINT on all HSC pseudo-bulks and compared young and old HSCs, finding a marked increase in the activity of NF-I, Runx, Ets, Gata and AP-1 (for example, Fos, Jun)^10^ (Fig. 4g and Supplementary Table 6). Considering that comparison of all young and old HSCs might be confounded by changes in HSC subpopulation composition during ageing, we performed this analysis using only megakaryocyte (Mk)-biased and multilineage subpopulations, respectively. Subpopulation-specific comparison yielded overall similar results, indicating that the majority of changes are shared across subpopulations (Extended Data Fig. 9a–d). However, a subset of Ets motifs, in particular Spi1/Spib/Spic and Elf, show age-associated downregulation specifically in the multilineage subpopulation, indicating subpopulation-specific TF changes with ageing (Extended Data Fig. 9c,d).

To examine the sequence motifs learned by seq2PRINT in an unbiased and comprehensive manner, we identified de novo motifs with differential activity (Fig. 4g, Extended Data Fig. 9e and Supplementary Table 6), including the loss of many CG-rich de novo motifs in ageing, possibly related to recognition by DNA-binding factors of methylation changes characteristic of ageing in a variety of contexts^17^ (Extended Data Fig. 9f). We found an increase in activity at composite motifs containing an Ets homo- or heterodimer with Gata, AP-1 and Runx motifs (Fig. 4g–h and Extended Data Fig. 9g–h). Furthermore, we found that Ets/Runx composite motifs are particularly enriched in old multilineage HSCs (Extended Data Fig. 9h,i). Supporting this finding, previous work has proposed a role for Ets/Runx co-occurrence in HSC maintenance and myeloid fate^43^. Mechanistically, Ets binding of DNA is negatively regulated by an autoinhibitory domain that is released following cobinding with Runx or second Ets-family TF, and physical interactions have been shown in two experimentally determined structures (PDB-4L0Z and PDB-2NNY; Fig. 4h)^44,45^. To test whether seq2PRINT-predicted Ets composite motifs could represent similar direct interactions, we used AlphaFold3 (ref. ^46^) to predict structures and found that all seq2PRINT-identified Runx/Ets configurations showed a similar physical interaction regardless of orientation. In support of the validity of these predictions, AlphaFold3 structures based on known Ets/Ets and Ets/Runx motif configurations were highly concordant with the experimentally measured structure (root mean-squared deviation between predicted structures for motif no. 10 and PDB-4L0Z, 0.825 Å), and solvent-inaccessible bases at the interface with each TF matched the correct core motif (Fig. 4h, Extended Data Fig. 9j, Supplementary Notes and Supplementary Table 7). seq2PRINT thus reveals rewiring of TFs during ageing and, through de novo motif analysis complemented by AlphaFold3, predicts a wide diversity of Ets and Runx cobinding arrangements implicated in ageing and HSC self-renewal. We also provide TF scores across pseudo-bulks to enable exploration of TFs associated with gene programs (Supplementary Table 5).

**Extended Data Fig. 10:**
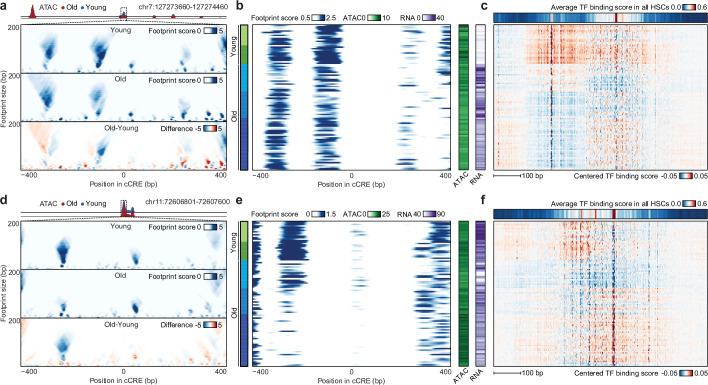
Examples of age-associated cCRE reorganization. **a**-**f**, Example promoter regions with age-associated cCRE reorganization, showing results for chr7:127273660-127274460 (**a**-**c**), chr11:72606801−72607600 (**d**-**f**). **a**, **d**, Multiscale footprints in young and old HSCs, and old-young differences. **b**, **e**, Nucleosome footprints (100 bp scale) across pseudo-bulks. Rows represent individual HSC pseudo-bulks and columns represent single base-pair positions. Left colour bar represents the HSC sub-populations with the same colour coding in Fig. 3e,f. Right colour bars: chromatin accessibility and RNA levels of the corresponding gene across pseudo-bulks. **c**, **f**, Top colour bar shows the average seq2PRINT-predicted TF binding scores across pseudo-bulks. Heatmap shows TF binding scores in each pseudo-bulk, after centring by subtracting column averages.

Across multiple systems, various studies have described a global loss of nucleosomes associated with ageing and senescence, although debate remains as to whether this loss is global or restricted to specific TF-associated sites^47^. To further explore the epigenetic decline of HSCs, we used PRINT to measure nucleosome occupancy across cCREs during ageing. We observed widespread loss of nucleosome footprints across cCREs in old HSCs (Fig. 4i and Extended Data Fig. 9k–q). We note that 38% of cCREs with differential nucleosome footprints did not show significant accessibility changes, highlighting the utility of footprinting in resolving changes. A reduction in nucleosome footprints does not necessarily indicate a loss of nucleosome number across the genome, however, because this could also result from loss of nucleosome positioning/phasing and the analysis is mainly limited to cCREs. To determine whether specific TFs were altered at these loci, we used seq2PRINT motifs and found strong enrichment for aging down-regulated TFs, including Yy1, Nrf1 and Ctcf, and gain of up-regulated TFs Nfyb, Atf, Jun and Klf (Fig. 4j,k and Extended Data Figs. 9m,n,p,q and 10a–f). Notably, Yy1, Nrf1 and Ctcf were also predicted to regulate nucleosome position in diverse cell lines (Fig. 2c,g). Yy1 has previously been shown to be a regulator of HSC self-renewal and quiescence^48^. Overall, we find widespread alteration of nucleosome footprints and rewiring of TF binding across ageing, suggesting a dysregulation of cCRE structure in ageing (Fig. 4l).

## Discussion

Our results demonstrate the complex dynamics of TF binding and nucleosome repositioning at cCREs across cell differentiation and ageing. Previous footprinting studies have suggested that TF binding is mostly determined by wholesale opening or closing of cCREs, rather than by differential binding of TFs within the same cCRE^11^. By contrast, our study identifies that cCREs are occupied by distinct sets of TFs across cell types. This is exemplified by the *SPI1*/PU.1 and *Wasl* promoter analysis above (Figs. 3b and 4k), in which multiple configurations of nucleosomes and TFs are observed despite similar levels of overall accessibility, showing mechanisms of gene regulation that would be missed by standard chromatin accessibility analysis. In direct support of this model, studies mapping TF binding by ChIP–seq report that TFs switch during development^49–51^. Along continuous trajectories of haematopoietic differentiation, we find that cCREs widen sequentially around central TFs, with flanking TFs binding at later stages of development, suggesting that the establishment of enhancers is an analogue rather than a digital process.

More broadly, PRINT generates an image of all DNA-binding proteins simultaneously in a given cell population. Modelling footprints with seq2PRINT infers TF binding regardless of its direct footprint strength, enables de novo identification of TF motifs and cooperative binding and suggests that TFs have specialized functions such as remodelling or stabilizing of neighbouring nucleosomes. These attributes contrast seq2PRINT with ChromBPNet^15^, which is tuned to predict accessibility profile rather than interpret the sequence features underlying footprints. Using LoRA^28^, we reduce the computational burden of footprint prediction, enabling us to extend seq2PRINT to single-cell epigenomics data. We anticipate that this approach will enable methods that connect high-resolution footprinting with diverse epigenomic data types such as genome structure and local gene expression. Similarly, identification of TF binding and attribution of footprints to specific sequences at base-pair resolution may also ascribe new functions to disease-causing genetic variation previously obscured by peak-based analyses^52^.

These methods have limitations. Our method does not incorporate transposed fragment length, which could be an additional feature of the analysis^21^. In addition, because deep learning requires multiple examples to identify patterns, rare or non-canonical binding events could be missed. Furthermore, footprinting of scATAC–seq requires pseudo-bulking to yield sufficient Tn5 insertion events to identify footprints. We envision that PRINT may be complemented by other methods, such as methyltransferase-based, single-molecule footprinting^53,54^ and DNA sequence mutagenesis assay^32,55^, to further analyse the structure and function of specific cCREs. However, because ATAC–seq has been broadly adopted and widely used for single-cell assay, seq2PRINT may uniquely enable both retrospective and prospective studies that atlas footprints across a broad range of healthy and diseased human tissues. Taken together, our approach extracts a rich multidimensional feature space from unidimensional chromatin accessibility data to reveal the dynamic structure of cCREs across high genomic and cell-state resolution.

## Methods

### Experimental methods

#### Cell culture

HepG2 cells (ATCC HB-8065; authenticated by short tandem repeat profiling and tested for mycoplasma by ATCC) were cultured in DMEM (catalogue no. 11965092, ThermoFisher), with the addition of 10% fetal bovine serum (FBS) and 1% penicillin/streptomycin. Cells were incubated at 37 °C in 5% CO_2_ and maintained in exponential phase, followed by digestion with TrypLE express (catalogue no. 12604013, ThermoFisher) for preparation of single-cell suspensions.

#### BMMC sample processing

Frozen human bone marrow mononuclear cells (BMMCs, AllCells; commercial donor cell experiment conducted under approval from Harvard Institutional Review Board) were thawed in a 37 °C water bath for 1 min and transferred to a 15-ml centrifuge tube. Next, 10 ml of prewarmed DMEM with 10% FBS was added dropwise to cells, which were then spun at 400*g* for 3 min at room temperature. Following removal of supernatant, cells were washed twice in 0.5 ml of PBS with 0.04% bovine serum albumin (BSA). To deplete neutrophils, cells were resuspended in 100 μl of chilled DPBS with 0.2% BSA and 10 μl of human TrueStain FcX (BioLegend, catalogue no. 422302) and incubated on ice for 10 min to reduce non-specific labelling. Cells were then incubated on ice for a further 30 min following the addition of 0.5 μl of biotin antihuman CD15 antibody (BioLegend, catalogue no. 301913). Following immunostaining, 25 μl of MyOne T1 beads was added to the sample for 5 min at room temperature, to capture neutrophils. We then added 900 μl of DPBS with 0.2% BSA to dilute the sample. The sample was placed on a magnet for 3 min, and 1 ml was transferred to a new tube while the sample was on the magnet. Cells were then ready for fixation and the SHARE–seq experiment.

#### Fixation

Cells were centrifuged at 300*g* for 5 min and resuspended at 1 million cells ml^−1^ in PBSI. Cells were fixed by the addition of formaldehyde (catalogue no. 28906, ThermoFisher) to a final concentration of 1%, and incubated at room temperature for 5 min. Fixation was stopped by the addition of 56.1 μl of 2.5 M glycine, 50 μl of 1 M Tris-HCl pH 8.0 and 13.3 μl of 7.5% BSA on ice. The sample was incubated at room temperature for 5 min and then centrifuged at 500*g* for 5 min to remove supernatant. All centrifugation was performed on a swing-bucket centrifuge. The cell pellet was washed twice with 1 ml of PBSI and centrifuged at 500*g* for 5 min between washings. Cells were resuspended in PBS with 0.1 U μl^−1^ Enzymatics RNase Inhibitor and aliquoted for transposition.

#### SHARE–seq

Following fixation, SHARE–seq was performed as previously described^27^, with the following modifications. To improve transposition, this was performed using preassembled Tn5 (seqWell, Tagify(TM) SHARE-seq Reagent). To improve RNA capture, we added poly-A to transcripts before reverse transcription. To do this, transposed cells (60 μl) were mixed with 240 μl of poly(A) mix (final concentration of 1× Maxima RT buffer, 0.25 U μl^−1^ Enzymatics RNase Inhibitor, 0.25 U μl^−1^ SUPERase RI, 0.018 U μl^−1^ *Escherichia coli* poly-A enzyme (New England Biolabs catalogue no. M0276L) and 1 mM ribonucleic ATP). The sample was aliquoted to 50 μl per PCR tube and incubated at 37 °C for 15 min.

#### Quantification and sequencing

Both scATAC–seq and scRNA-seq libraries were quantified with the KAPA Library Quantification Kit and pooled for sequencing. Single-cell libraries were sequenced on the Nova-seq platform (Illumina) using a 200 cycle kit (read 1, 50 cycles; index 1, 99 cycles; index 2, 8 cycles; read 2, 50 cycles). Bulk libraries were sequenced on the Nova-seq platform (Illumina) using a 100 cycle kit (read 1, 50 cycles; index 1, 8 cycles; index 2, 8 cycles; read 2, 50 cycles).

#### SHARE–seq data preprocessing

SHARE–seq data were processed using the SHARE-seqV2 alignment pipeline (https://github.com/masai1116/SHARE-seq-alignmentV2/) and aligned to hg38. Open chromatin region peaks were called on individual samples using MACS2 peak caller (v.2.2.9.1)^56^ with the following parameters: --nomodel –nolambda –keep-dup -call-summits. Peaks from all samples were merged, and those overlapping with ENCODE blacklisted regions (http://mitra.stanford.edu/kundaje/akundaje/release/blacklists/hg38-human/hg38.blacklist.bed.gz) were filtered out. Peak summits were extended by 150 bp on each side and defined as accessible regions (for footprinting analyses, these peaks were later resized to 1,000 bp in width). Fragment counts in peaks and TF scores were calculated using chromVAR (v.1.24.0)^10^. Cell barcodes with under 30% reads in peaks (fraction of reads in peaks, FRiP) or under 250 unique fragments were removed. Aligned reads were then intersected with peak window regions, producing a matrix of chromatin accessibility counts in peaks (rows) by cells (columns). To examine cell identity, cisTopic (50 topics)^57^ was used for dimension reduction, followed by Louvain clustering. Progenitor populations were subclustered to obtain finer cell identity. The data were projected into two-dimensional space by UMAP^58^. Seurat v.3 (v.5.0.3)^59^ was used to scale the digital gene expression matrix by total UMI count, multiplied by the mean number of transcripts, and values were log transformed.

#### Generation of BAC naked DNA data

We selected 25 chromatin regions based on overlap with a manually selected set of key transcription factors and differentiation related genes. Bacterial artificial chromosome (BAC) clones (BACPAC Resources) were cultured in lysogeny broth for 14 h. BAC DNA was extracted using a ZR BAC DNA Miniprep Kit (Zymo, catalogue no. D4048), following the manufacturer’s instructions. Purified DNA was quantified using Qubit (ThermoFisher). BAC DNA was tagmented similarly to the SHARE–seq ATAC–seq experiment. Briefly, 50 ng of BAC DNA from multiple clones was pooled for tagmentation following the SHARE–seq transposition condition. Tagmented DNA was purified using a Qiagen Minelute PCR clean-up kit and then PCR amplified for seven cycles. To minimize batch effect, we generated five biological replicates and pooled all materials for sequencing. The library was sequenced on a Nova-seq platform (Illumina) using a 100 cycle kit (read 1, 50 cycles; index 1, 8 cycles; index 2, 8 cycles; read 2, 50 cycles). Sequencing data were processed as for SHARE–seq ATAC–seq data.

#### Generation of human genomic DNA data

Human (female) genomic DNA was obtained from Promega (catalogue no. G1521). Genomic DNA was digested with the restriction enzyme SbfI-HF (New England Biolabs, catalogue no. R3642L). For each of two replicates, 25 μg of DNA was digested with 200 units of SbfI-HF in a 500 μl reaction at 37 °C overnight. The digested DNA was run on a 1% agarose gel and fragments corresponding to 2–2.5, 2.5–3, 3–4 and 4–5 kb were excised. All fragments from replicate 1 and the 3–4 kb fragment from replicate 2 were purified using the QIAquick gel extraction kit (Qiagen). Purified products were concentrated using the Zymo DNA clean and concentrator kit. Finally, tagmentation and library preparation were performed following the above protocol for BAC DNA tagmentation, using the same ratio of DNA mass to Tn5 when a lower amount of DNA was recovered following size selection.

#### In vitro footprinting

BAC DNA (50 ng) was incubated with either recombinant c-MYC/MAX (Active Motif, catalogue no. 81087) or CEBPA (OriGene, catalogue no. TP760685), tagmentation buffer (20 mM Tris, 10 mM MgCl_2_ and 20% dimethylformamide) and water in a 40-μl volume at room temperature for 1 h. Next, a master mix consisting of 0.15 μl of preassembled Tn5 (seqWell, Tagify(TM)), 4.85 μl of dilution buffer (50 mM Tris, 100 mM NaCl, 0.1 mM EDTA, 1 mM DTT, 0.1% NP-40 and 50% glycerol) and 5 μl of tagmentation buffer was added to the samples for tagmentation in a 50-μl final volume (final TF concentrations of 0, 50 and 100 nM) for 30 min at 37 °C. Tagmented DNA was purified using a Qiagen Minelute PCR clean-up kit and subsequently PCR amplified for five cycles. Samples were then pooled and sequenced on a Nova-seq platform (Illumina). Sequencing data were processed in the same way as bulk ATAC–seq data.

#### Ageing multiome experiment

Mouse experiments were approved and performed in compliance with Harvard University’s Institutional Animal Care and Use Committee. C57BL6 mice were obtained from either Jackson Laboratory or the National Institute on Aging Aged Rodent Colony (Charles River Laboratory), housed at a density of between two and five per cage in standard ventilated racks and provided with food and water ad libitum in a specific-pathogen-free facility accredited by the Association and Accreditation of Laboratory Animal Committee. Mouse cages contained Anderson’s Bed-o’Cob bedding (The Anderson Inc.), two nestlets (Ancare, 2 × 2-inch^2^ compressed cotton squares) and a red mouse hut (Bioserv). For HSC isolation and flow cytometry, cells from the bone marrow of long bones (two femurs and two tibias per mouse) from young (*n* = 10, 11 weeks old) and aged (*n* = 5, 24 months old) male C57BL/6 mice were flushed with a 21-gauge needle into staining medium (HBSS/2% FBS), pelleted and resuspended in ACK lysis buffer for 5 min on ice. Mouse numbers were determined by the anticipated cell yield and input needs for single-cell assay; cells from mice were pooled so that no blinding or randomization was performed. Cells were then washed with staining medium, filtered through a 40-micrometre cell strainer, pelleted and incubated with the following cocktail of rat anti-mouse, biotin-conjugated lineage antibodies on ice for 30 min: CD3 clone C145-2c11 (BioLegend, catalogue no. 100304; 1:100), CD4 clone GK15 (BioLegend, catalogue no. 100404; 1:400), CD5 clone 53-7.3 (eBioscience, catalogue no. 13-0051-85; 1:400), CD8 clone 53-6.7 (BioLegend, catalogue no. 100704; 1:400), CD19 clone 6D5 (BioLegend, catalogue no. 115504; 1:400), B220 clone RA3-6B2 (BioLegend, catalogue no. 103204; 1:200), GR1 (Ly6-G/Ly6-C) clone RB6-8C5 (eBioscience, catalogue no. 13-5931-82; 1:400), Mac1/CD11b clone M1/70 (BioLegend, catalogue no. 101204; 1:800) and Terr119 clone TERR-119 (BioLegend, catalogue no. 116204; 1:100). Cells were then washed in staining medium, with a small aliquot reserved for each sample to serve as a non-depleted control, and lineage depleted using sheep anti-rat Dynabeads (Invitrogen, catalogue no. 1135) on a magnet. Cells were washed, pelleted and incubated with the following cocktail of anti-mouse antibodies on ice for 45 min to identify HSCs: Pacific Orange Streptavidin (Invitrogen, catalogue no. S32365; 1:500), PE/Cy7 Sca1(Ly-6a/E) clone D7 (eBioscience, catalogue no. 25-5981-82; 1:200), APC cKit clone 2B8 (BD Pharmingen, catalogue no. 553356; 1:200), FITC CD48 clone HM48-1 (BioLegend, catalogue no. 103403; 1:200) and PE CD150 clone Tc15-12F12.2 (BioLegend, catalogue no. 115904; 1:200). Following incubation, cells were washed and resuspended in staining medium, and 7-AAD (BD Pharmingen, catalogue no. 559925; 1:50) added immediately before flow cytometry. Cell sorting of HSCs (Live Lin^−^ Sca1^+^ cKit^+^ CD48^−^ CD150^+^) was performed on a BD FACS Aria II. Cells within the same age group were sorted into the same tube for subsequent sequencing. Data analysis was performed using BD FACS Diva (v.8.0.2) and FlowJo (v.10.8.2) software. Data processing was performed using Cell Ranger ARC 2.0.0.

Following sorting, nuclei were isolated following the 10x Genomics demonstrated protocol Low Cell Input Nuclei Isolation, which is described in the CG000365 User Guide. Nuclei were then processed using the Chromium Single Cell Multiome ATAC + Gene Expression kit (10x Genomics), following the manufacturer’s instructions, to obtain between 2,000 and 10,000 cells per sample. Libraries were sequenced on an Illumina Next-seq system using the following sequencing formats: read 1, 28; i7 index, 10; i5 index, 10; read 2, 44 (scRNA-seq); read 1, 30; i7 index, 8; i5 index, 24; read 2, 30 (scATAC–seq). Data processing was performed using CellRanger ARC 2.0.0 software from 10x Genomics.

#### Tn5 sequence bias modelling

##### Getting Tn5 insertion counts

The ends of fragment files were shifted by +4/−4 to obtain the centre of the 9 bp staggered end created by Tn5 transposition. The number of insertions at each single base-pair position within each cCRE from each sample was then quantified and stored in a sample-by-cCRE-by-position three-dimensional tensor for rapid data retrieval.

##### Data preprocessing

The model takes local DNA sequence context as input and predicts single base-pair resolution Tn5 bias. To this end, the ±50-bp DNA sequence surrounding each position of interest is encoded by one-hot encoding into a 101 × 4 matrix and used as model input. For the prediction target, we use local relative Tn5 bias as the target value. More specifically, the raw Tn5 insertion count at each position is divided by the average Tn5 insertion count within a ±50-bp window. Positions with low local coverage (fewer than 20 insertions per base pair) were removed to guarantee quality of training data. To facilitate model training, the resulting observed Tn5 bias values were log_10_ transformed and rescaled. For dataset partition, we randomly split all BACs into 80%, 10% and 10% for the training, validation and test sets, respectively; in other words, all data originating from the same BAC belonged to the same partition. This is to prevent overlapping local sequence contexts being placed in both training and testing datasets, which might lead to overestimation of performance. To guarantee equal coverage of examples with different bias levels, we binned all training examples into five bins based on their Tn5 bias value, and upsampled each bin so that all bins ended up with the same number of examples. In addition, given the symmetric nature of Tn5 insertion, we generated reverse-complement sequences of training examples as data augmentation. The original and reverse-complement data were combined, shuffled and then used for model training.

##### Model architecture

The convolutional network consists of three convolution and max-pooling layers and two fully connected layers. Each convolution and max-pooling layer performs convolution, rectified linear unit (ReLU) nonlinear activation^60^ and max pooling sequentially. We used 32 filters of width 5 for each layer, along with the ‘same’ padding mode and stride size of 1. The two following fully connected layers have output dimension of 32 and 1, respectively; ReLU activation is used by the first fully connected layer and linear activation by the second (that is, the final output layer).

##### Model training and evaluation

The model was trained on the training set, and hyperparameters were optimized based on performance on the validation set. Final performance of the frozen model was evaluated on the test set. The model was implemented using Keras^61^, trained with mean-square error as loss function and optimized using the Adam optimizer^62^ with default parameters. Training was performed with a batch size of 64 and early stopping based on model loss on the validation set.

##### Benchmarking with other Tn5 bias models

Methods including *k*-mer models (*k* = 3, 5, 7) and PWM methods (single nucleotide and dinucleotide) were included in benchmarking. For *k*-mer methods, the foreground and background frequencies for all possible *k*-mer sequences were quantified. Foreground/background frequency ratio was used as estimated Tn5 bias for the corresponding *k*-mer. For single-nucleotide PWM, we calculated foreground and background base frequencies within a ±10-bp window (total length, 21 bp) and computed the PWM of Tn5 insertion. Dinucleotide PWM scores were calculated using TOBIAS^18^ with default settings. It is suggested to train custom ChromBPNet bias models on inaccessible chromatin regions for each dataset, to represent Tn5 sequence bias and achieve the highest-quality models^63^. Accordingly, we trained ChromBPNet on HepG2 and K562 ATAC–seq data to evaluate performance at its recommended setting.

##### Genome-wide Tn5 bias reference tracks

Sequences of reference genomes for *Homo sapiens* (hg38), *Mus musculus* (mm10), *Drosophila melanogaster* (dm6), *Saccharomyces cerevisiae* (sacCer3), *Caenorhabditis elegans* (ce11), *Danio rerio* (danRer11) and *Pan troglodytes* (panTro6) were downloaded from the UCSC genome browser website^64^ (https://hgdownload.soe.ucsc.edu/goldenPath/). The aforementioned Tn5 bias neural network model was applied to each position in the reference genomes to generate genome-wide Tn5 bias tracks.

#### Computation of footprint scores

For detection of DNA–protein interactions at different scales within cCREs, we implemented a framework for computing footprint scores for each base-pair position in the cCRE. In short, for each single base-pair position, we define a centre footprint window and flanking windows on both sides (Fig. 1a). We then calculated the observed ratio of centre/(centre + flanking) Tn5 insertion counts. The foreground observed ratio was compared with background distribution to calculate statistical significance, which was then converted to a footprint score.

##### Estimation of background dispersion

Given a specific combination of centre bias, flanking bias and local coverage, we expect a certain distribution of centre/(centre + flanking) insertion ratio when no protein is bound; this is defined as background distribution. Such background distribution can be estimated using BAC naked DNA Tn5 insertion data. To this end, we first randomly sampled 100,000 positions from the BAC dataset and retrieved their local coverage (defined as the total insertion number in centre and flanking areas), centre bias and flanking bias. Then, for each sampled position A, we identified the 500 nearest-neighbour positions (NN_1_–NN_500_) in the three-dimensional space of centre bias, flanking bias and local coverage. To ensure that each dimension was weighed equally, the values of each dimension were first normalized to zero mean and unit variance. The 500 nearest-neighbour observations can be considered as background observations with nearly identical bias and coverage, with the centre/(centre + flanking) ratio of NN_1_–NN_500_ forming the background distribution of position A. Therefore, for each of the 100,000 sampled positions, we can calculate the mean and standard deviation of its background ratio distribution. This allows us to train a background dispersion model that takes the tuple (centre bias, flanking bias and local coverage) as input and efficintly predicts the mean and standard deviation of background distribution. To ensure that the model is exposed to training examples with a wide range of local coverage, we downsampled the BAC dataset to 50, 20, 10, 5 and 1% of the original sequencing depth. Finally, we trained a neural network with a single hidden layer (32 nodes, ReLU activation^60^) and linear output layer activation. The dataset was randomly split into 80% training, 10% validation and 10% test. The model was implemented using Keras^61^, and trained on the training dataset with mean-squared error loss using the Adam optimizer^61,62^. Early stopping was determined using loss on the validation set, and performance of the final model was evaluated on the test set. In addition, we trained separate models for each footprint radius due to the marked differences in total centre or flank bias when footprint radius varied (Supplementary Notes).

##### Calculation of footprint scores

For each position in the cCRE, we define a centre footprint window and flanking windows on both sides. We first calculate the foreground observed centre/(centre + flanking) ratio of Tn5 insertion counts, then we apply the pretrained background dispersion model to calculate the mean and standard deviation of its background distribution. We next use a lower-tailed *z*-test to calculate the *P* value for footprinting. If the observed ratio is significantly lower than background distribution, this position is likely to be bound by a protein. More specifically, to avoid calling footprints at positions at which only one flanking side shows higher Tn5 insertion than the centre window but not the other, we perform centre-versus-left and centre-versus-right tests separately and retain the higher *P* value (Supplementary Notes). The −log_10_(*P* values) are smoothed by running-max and running-mean smoothing and then used as the final footprint scores.

##### Aggregate footprinting

For calculation of aggregate footprints, Tn5 insertions surrounding TF or nucleosome binding sites across the genome are first aggregated and then used to calculate footprint scores. For TFs, we selected sites with a matched TF motif using motifmatchr version 1.24.0 (ref. ^65^) (*P* cutoff 1 × 10^−5^) and those overlapping with a ChIP–seq peak of the corresponding TF. For motif matches on the reverse strand, the Tn5 insertion profile surrounding the motif is inverted so that insertions for different sites are aligned in the same direction.

#### Footprint-to-TF prediction

Note that seq2PRINT was chosen as our primary TF binding predictor, but we still provide this lightweight footprint-to-TF prediction model for its speed advantages. For comparison between footprint-to-TF prediction and seq2PRINT-based TF binding prediction, see ‘Multiple methods to predict TF binding from multiscale footprinting’ in Supplementary Notes.

To predict the landscape of TF binding, we trained a binary classifier that predicts whether any TF motif site is bound by the corresponding TF. Motif sites are identified by the matchMotifs function in the motifmatchr package^65^. All sites with a matching *P* value below 5 × 10^−5^ are retained. For any TF motif site, we use multiscale (20, 40, 60, 100, 160 or 200-bp diameter) footprints within a ±100-bp local area centred around the motif, as well as a motif match score as input to the model. The motif match score returned by the matchMotifs function is quantile transformed to uniform distribution. As a result, by combining of the 201-dimensional footprint vectors from six different scales with a single motif match score, we create a 1,207-dimensional vector as the final model input. The first 1,206 dimensions of footprint scores are standardized individually to zero mean and unit variance. For the prediction target, we assign a label of 1 to all sites overlapping with a ChIP peak of the same TF, and a label of 0 to those not overlapping with ChIP. Some TFs were found to have a very low percentage of motif sites overlapping with ChIP (10% or under), potentially due to low quality of the motif or ChIP dataset, and these were removed from model training and testing. We also added additional negative examples as well as reverse-complement examples for data augmentation.

For data partition, HepG2 data were used as training data and GM12878 data (previously published in the original SHARE–seq paper^27^) as validation. After fixing model hyperparameters, HepG2 and GM12878 data were combined into a larger training dataset to train a final footprint-to-TF model. The final model was tested on K562 scATAC data^66^, as well as on three cell types (naive B cells, CD14 monocytes and late-erythroid cells) in the human BMMC SHARE–seq dataset.

##### Model architecture and training

The TF binding prediction model is a neural network model with two hidden layers (128 + 32 nodes). ReLU activation^60^ is used by both hidden layers, and sigmoid activation by the final output layer. The model was implemented using Keras^61^, and trained on the training dataset with a batch size of 128 using the Adam optimizer^67^. Binary cross-entropy is used as the loss function. Early stopping was used based on model loss on the validation set.

##### ChIP validation and benchmarking with previous methods

For evaluation of model performance, we used ChIP–seq as ground truth and validated predicted binding events. HepG2 and GM12878 data were downloaded from UniBind^68^ (https://unibind.uio.no/search) for model training. ChIP–seq for BMMC cell types was downloaded from cistromeDB^69^. For benchmarking with previous methods, to ensure that we included only high-quality TF binding sites, we downloaded K562 ChIP-based TF binding data from UniBind. For cistromeDB datasets, we applied quality control filters as specified on the cistromeDB website (http://cistrome.org/db/#/about). More specifically, we included the following filters: FRiP 0.01 or over, FastQC 0.25 or over, uniquely mapped ratio 0.6 or over, peaks with fold change above ten, 500 or more, peaks union DNase I hypersensitive site ratio 0.7 or above and PCR bottleneck coefficient 0.8 or above. Datasets with the following cell type labels were included: Monocyte, B Lymphocyte, Erythroid cell, Erythroid Progenitor Cell and Erythroid progenitor. If there was more than one dataset for the same TF, the intersection of all datasets for the same TF was retained as the final list of high-confidence binding sites for model training.

The K562 datasets from UniBind were used for benchmarking with previous methods, including DNase I footprinting, TOBIAS and sequence attribution scores obtained from ChromBPNet. In short, the same ATAC–seq data were used as input to all ATAC footprinting methods and ChromBPNet, whereas DNase I footprinting results for K562 were obtained from ENCODE datasets ENCLB253REF, ENCLB843GMH and ENCLB096YUZ from ref. ^11^. To guarantee fair comparison, we used the same set of motif match positions (previously published in ref. ^70^) as candidate binding sites, and mapped the predicted scores of each method to these sites for comparison. For TFs with multiple UniBind datasets, their intersection was used for benchmarking. Then, for each method, we ranked candidate sites by predicted binding score and evaluated the precision of prediction using the top 10% of sites. Visualization of predicted and ground truth binding sites was done with the Gviz package (v.1.46.1)^71^. Furthermore, to evaluate the false-positive rate of each model, we also tested all three ATAC-based models on our BAC naked DNA data. The same data were used as input to each model, and the number of predicted binding events was used to represent false-positive predictions.

#### Footprint-to-nucleosome prediction

##### Input data

For prediction of nucleosome occupancy, we trained a regression neural network model. For any genomic position, we used multiscale (20, 40, 60, 100 and 160 bp in diameter) footprints within a ±100-bp local area as input to the model. A scale of 200 bp was not included, to prevent the model from learning the co-occupancy of adjacent nucleosomes. To train this model, we used previously published chemically mapped nucleosome occupancy data on *S. cerevisiae*^72^ as the training label, and computed multiscale footprints using previously published *S. cerevisiae* ATAC–seq data^20^ as training input. We retained observations in regions with local ATAC–seq coverage above 10, and rescaled the 5 and 95% percentiles of nucleosome occupancy values to 0 and 1, respectively. For data partition, we randomly split all data by chromosomes into training (chrVII, chrXI, chrIX, chrI, chrV, chrX, chrVIII and chrXII), validation (chrIV and chrII) and test (chrVI, chrXVI, chrXIII, chrIII, chrXIV and chrXV) sets.

##### Model architecture and training

The nucleosome prediction model is a neural network model with two hidden layers (64 + 16 nodes). ReLU activation is used by both hidden layers, and linear activation by the final output layer. The model was implemented using Keras. The model was trained on the training dataset with a batch size of 128 using the Adam optimizer. Mean-squared error was used as the loss function. Early stopping was used based on model loss on the validation set.

##### Performance evaluation

Model performance was evaluated using data on the test yeast chromosomes mentioned above. In total, 859 regions of length 5 kb on the test yeast chromosomes (chrVI, chrXVI, chrXIII, chrIII, chrXIV and chrXV) were used for testing. We detected summits of predicted nucleosome signal and ground truth nucleosome occupancy as predicted and observed nucleosomes, respectively. Precision was calculated as the percentage of predicted nucleosomes having a ground truth nucleosome within a certain cutoff distance (50 or 75 bp in this study). Recall was calculated as the percentage of ground truth nucleosomes having a predicted nucleosome within the same cutoff distance.

#### Seq2PRINT

##### Model architecture and training

The seq2PRINT model is a convolutional neural network that takes one-hot encoded DNA sequences (a DNA sequence of length *L* encoded into an *L* × 4 matrix, where each row has one element set to 1 representing the specific nucleotide) as input and predicts the corresponding multiscale footprints at base-pair resolution. The architecture is depicted in Extended Data Fig. 4a. To facilitate articulation of the architecture, we divide the seq2PRINT model into three parts. Although the architecture of seq2PRINT can be flexible depending on the available computational resources and the depth and scale of the training data, for all results in this work we build the model and choose the hyperparameters as follows.

The first convolutional layer consists of 1,024 filters of width 21 bp with Gaussian error linear unit (GELU) activation, aiming to capture informative sequence patterns from the input DNA sequences (that is, sequence motifs). The output is subsequently passed to eight layers of convolutional blocks with residual connection. Each convolutional block consists of one grouped dilated convolutional layer (*n* filters = 1,024, width = 3, groups = 8, dilation = 2^*i*^, *i* = 1,…, 8), followed by a position-wise feed-forward layer (implemented as a convolutional layer with *n* filters = 1,024, width = 1). Batch normalization layers with GELU activations are inserted between these convolutional blocks. The increased dilation rate results in an expanding receptive field for the neural network, capturing the relationship of the sequence patterns and their context. With eight layers of the convolutional block, seq2PRINT has a receptive field of ±920 bp for each cCRE. The use of grouped convolutional layers enables the construction of wider models that capture richer information with limited parameters, providing a regularization effect and reduced computational complexity. Finally, the output of the stacks of convolutional blocks is passed to the output layers.

In this paper we designed two output layers, a multiscale footprint layer (a convolutional layer of filter width 1) that outputs the multiscale footprints and an accessibility layer (a global average pooling layer followed by a fully connected layer) to predict the number of Tn5 insertions in a specific cCRE.

To facilitate the training of the seq2PRINT model, we implemented batch-efficient multiscale footprint calculation on graphics processing units, which follows the same mathematical models as the described footprint calculation, the only difference being that it outputs the *z*-statistics rather than the *P* value calculated from the *z*-test.

During training, model weights are updated to minimize the following loss function:

$$\begin{array}{c}{\rm{Loss}}={{\rm{Loss}}}_{{\rm{footprint}}}+{{\rm{Loss}}}_{{\rm{accessibility}}}\\ {{\rm{Loss}}}_{{\rm{footprint}}}={\rm{MSE}}({{\rm{footprint}}}_{{\rm{PRINT}}},{{\rm{footprint}}}_{{\rm{pred}}})\\ {{\rm{Loss}}}_{{\rm{accessibility}}}={\rm{MSE}}(\log (1+{n}_{{\rm{obs}}}),\log (1+{n}_{{\rm{pred}}})),\end{array}$$

where MSE represents mean-squared error ${\rm{MSE}}(x,y)=\sum {(x-y)}^{2}$, ${{\rm{footprint}}}_{{\rm{PRINT}}}$ and ${{\rm{footprint}}}_{{\rm{pred}}}$ represent multiscale footprints calculated by the PRINT framework and seq2PRINT model, respectively, and *n*_obs_ and *n*_pred_ represent the observed and predicted Tn5 insertions, respectively, in this region.

Notably, the gradient back-propagation for the accessibility layer is broken before the convolutional blocks: in other words, the sequence patterns and relationships among them learned by the preceding layers of the seq2PRINT model are driven solely by the multiscale footprint objective. The accessibility output layer and corresponding loss function reweight these learned sequence features for interpretation purposes only (see the following section for a detailed explanation). This design alleviates the need to choose weights between footprint loss and accessibility loss, and also makes seq2PRINT a footprint-driven sequence model, differentiating it from previous accessibility-driven models (for example, Basset^73^).

The seq2PRINT model is optimized with the Adam optimizer at a learning rate of 3 × 10^−4^, and uses exponential moving averages to stabilize and improve model convergence. In this study we used chromosome-based, fivefold cross-validation, with outputs across folds averaged for use as the final predictions.

##### Deriving sequence attribution scores

We use the DeepLIFT^74^ method to calculate sequence attribution scores, which represents how each base pair in a given input DNA sequence contributes to a specific scalar output from the trained seq2PRINT model. The output of the accessibility layer is a scalar for each region, making it naturally suitable as the target for DeepLIFT to calculate attribution scores. However, the multiscale footprint layer is not a scalar but a matrix of size *L* × number of scales. Therefore, we designed two strategies to summarize output footprints into a scalar value.

Both strategies involve conversion of the predicted *z*-statistics to log(*P* value) footprint scores as the PRINT framework. The first strategy involves manual inspection, as demonstrated in Fig. 2b, where we locate the region × scale of interest from the observed and predicted multiscale footprints, sum footprint scores within the region and calculate the corresponding sequence attribution score. The second strategy sums footprint scores within the whole peak region, and is more suitable for genome-wide calculations. Without further specification, we refer to sequence attribution scores calculated from the accessibility layer as count sequence attribution scores, and those calculated from the footprint layer as footprint sequence attribution scores.

For all results in this manuscript, we use 20 dinucleotide-shuffled input sequences as reference sequences for the DeepLIFT algorithm. We adapted implementation of the DeepLIFT algorithm from the DeepSHAP package to accommodate the custom nonlinear functions used in this framework; custom implementation is available as part of https://github.com/buenrostrolab/scPrinter.

##### De novo motif identification based on sequence attribution scores

TF-MoDISco (tfmodisco-lite v.2.2.1)^75^ was utilized to infer de novo motifs based on sequence attribution scores. Briefly, TF-MoDISco identifies local regions in input sequences with high sequence attribution scores (seqlets), then aligns and clusters similar seqlets into groups of de novo motifs. We set the number of seqlets as 1,000,000, and the remaining parameters as default. The de novo discovered motifs are assigned to known motifs using TomTom (meme suite v.5.5.7)^76^. To infer the matching of de novo motifs at cCREs, we use the software finemo (commit no. 830d7f3)^77^, which accepts both de novo motifs and sequence attribution scores.

##### TF binding prediction using attribution scores

The calculated sequence attribution scores highlight TF binding sites affecting footprints and accessibility. We thus trained a binary classifier that is similar to the footprint-to-TF model but, rather than multiscale footprints, it accepts both count and footprint sequence attribution scores.

The training and validation TF binding sites remain the same as in the previous footprint-to-TF model. For each motif site, the features include the count and footprint sequence attribution scores within a ±100-bp area centred around the motif, the motif-matching score and also the Pearson correlation between the motif of interest and sequence attribution score at the motif-matching site, in a 405-dimensional vector.

The TF binding model consists of three hidden layers (256, 128 and 64 neurons), with GELU activations and 0.25 dropout rates in between. The model is trained using the Adam optimizer, with binary cross-entropy as the objective function.

This TF binding model is the final TF binding prediction model that we used for Figs. 2–4, due to its superior performance. For a comparison between footprint-to-TF prediction and seq2PRINT-based TF binding prediction, see ‘Multiple methods to predict TF binding from multiscale footprinting’ in Supplementary Notes.

##### LoRA enables efficient sequence modelling for scATAC–seq data

To make the sequence model much more scalable on scATAC–seq with diverse cell types or cell states, we used the LoRA technique for parameter-efficient fine-tuning of neural network models. Specifically, for given scATAC–seq data, we first train a seq2PRINT model (referred to as the pretrained model) by aggregating Tn5 insertions over all cells in the dataset. Next, for each pseudo-bulk aggregating cells over specific cell states, we use the LoRA fine-tuning technique to derive a pseudo-bulk-specific seq2PRINT model. We describe this fine-tuning process as follows.

For any neural network layer parameterized by initial weights *W*_0_, where ${W}_{0}\in {R}^{{d}_{{\rm{p}}}}$, the LoRA model would learn an updating parameter $\varDelta W\in {R}^{{d}_{{\rm{p}}}}$, where *d*_p_ is the number of learnable parameters of the layer, and the sum of these two parameters is used as the weight parameter *W* for the resulting fine-tuned neural network $W={W}_{0}+\varDelta W$. For the residual grouped convolutional layer we used in seq2PRINT,

$${d}_{{\rm{p}}}=\frac{n\_{\rm{filter}}}{{\rm{group}}}\times n\_{\rm{filter}}\times {\rm{kernel}}\_{\rm{size}}$$

If we fine-tune the model individually for *n* pseudo-bulks of interest, this results in a total of *n* × *d*_p_ parameters to be learned.

Motivated by the LoRA model, we instead learn a low-rank decomposition of this updating parameter, guided by the intrinsic low ranking of cell states captured by cell embeddings. Specifically, with *d*_*e*_ as the dimension of single-cell embeddings and *r* as the rank hyperparameter, we learn two sets of weights, A and B, where $$A\in {R}^{{d}_{{\rm{e}}}\times r+r\times \frac{n\_{\rm{filter}}}{{\rm{group}}}}$$$$B\in {R}^{{d}_{{\rm{e}}}\times r+r\times n\_{\rm{filter}}\times {\rm{kernel}}\_{\rm{size}}}$$. This design reduces the number of parameters to be learned from *n* × *d*_p_ to $$r\times ({d}_{{\rm{p}}}^{* }+{d}_{{\rm{e}}})$$, where $${d}_{{\rm{p}}}^{* }=\frac{n\_{\rm{filter}}}{{\rm{group}}}\,+$$$$n\_{\rm{filter}}\times {\rm{kernel}}\_{\rm{size}} < \,\frac{n\_{\rm{filter}}}{{\rm{group}}}\times n\_{\rm{filter}}\times {\rm{kernel}}\_{\rm{size}}$$. In this study we chose *r* = 32, which is much smaller than the number of pseudo-bulks that we studied.

##### Predicted impact of TF motifs on footprints

To probe the relationships between multiscale footprints and DNA sequences learned by the seq2PRINT model, we generated the marginalized prediction from seq2PRINT given a known or de novo discovered motif. For a de novo discovered motif of interest, we first identified its consensus sequence by taking the nucleotide with highest probability at each position. We then randomly selected 25,000 cCREs from the dataset, planted the consensus sequence at the centre and averaged model predictions around the sampled cCREs. For a known motif, we gathered its motif-matching positions in cCREs, scrambled these and averaged model predictions. In both approaches, we calculated marginalized prediction as the difference between the presence and absence of a given motif .

#### Tracking TF binding dynamics across human haematopoiesis

##### Generation of pseudo-bulks

Single cells in the human BMMC dataset were first embedded into lower dimensional space using cisTopic^57^, and then grouped into 1,000 pseudo-bulks based on their spatial proximity in the cisTopic space. More specifically, we first sampled 1,000 cells as pseudo-bulk centres and then identified the *k*-nearest neighbours (KNN, *k* = 5,000) of each centre cell in the cisTopic space as other members of the same pseudo-bulk. We reasoned that sampling of centre cells with low local connectivity could help increase coverage of the state space by prevention of oversampling of densely connected local neighbourhoods. Therefore, we first randomly sampled 10,000 scaffold cells and used these to construct a KNN graph (*k* = 10), then selected the 1,000 cells with the lowest in-degree in the KNN graph as pseudo-bulk centres.

##### Computing pseudo-time

Pseudo-time along human haematopoietic lineages was computed using the Palantir package (v.1.0.0)^78^. To reduce computing time, we randomly sampled 100,000 cells from the human BMMC dataset as scaffold cells. The cisTopic embedding of both scaffold and pseudo-bulk centre cells was used as input to Palantir.

##### Footprinting repressor TFs

Because BHLHE40 has been studied extensively for its role in T cells, the BHLHE40 footprinting results in Extended Data Fig. 2 were obtained using CD8^+^ T cells from the human bone marrow dataset.

##### TF binding in erythroid and lymphoid cell cCREs

For erythroid and lymphoid development trajectories, we first identified relevant genes by selecting those with a correlation between RNA level and pseudo-time above 0.5, respectively. We then filtered cCREs within ±50 kb of the transcriptional start sites of related genes and correlation between cCRE accessibility and pseudo-time above 0.5. For all cCREs, we located candidate TF binding sites activated during development by retaining those at which the correlation between seq2PRINT TF binding score and pseudo-time was above 0. The remaining TF binding sites were used for dynamic quantifications. To quantify the inferred timing of binding, we rescaled the TF binding score of each binding site to [0,1] and calculated the area under the curve, with higher values representing earlier rise in binding signals and vice versa.

##### PCA measures TF binding pattern complexity at each cCRE

To reveal the complexity of TF binding patterns within each cCRE across diverse cell populations in the human BMMC dataset, we used a principal component analysis (PCA)-based method. To reduce computational complexity, we first collapsed the 1,000 LoRA fine-tuned seq2PRINT models (corresponding to 1,000 pseudo-bulks) into 20 models corresponding to 20 cell types. Model weights of pseudo-bulks corresponding to the same cell type were averaged during this process. We then generated cCRE-wide TF binding scores for these 20 cell types. We tiled each cCRE with 10-bp windows, and averaged TF binding scores within each window. PCA was then used as a dimension reduction method on this 20 × 10-bp window matrix for each cCRE, with the minimum number of PCs explaining 98% of the variance being used to quantify the complexity of TF binding patterns.

#### Characterizing age-related cCRE reorganizations

##### Data preprocessing

Cells with FRIP lower than 0.3 and depth less than 300 were first removed. In addition, we used ArchR (https://www.archrproject.com/)^79^ to calculate doublet scores for each single cell and removed those with the top 5% of doublet scores. The remaining cells were then processed with the Seurat V3 package (v.5.0.3)^80^. Cells were embedded into lower dimensional space using latent semantic indexing^81^ and then clustered. Seurat clusters corresponding to HSCs were selected for pseudo-bulking and downstream differential testing. Cells with the LinNeg FACS sort label were excluded from HSC-specific analyses. To identify representative cell states, we used SEACells (https://github.com/dpeerlab/SEACells/tree/main)^82^ to identify 100 representative cell states across HSCs. Representative cells were used as centres to form pseudo-bulks. Each pseudo-bulk was generated by serial inclusion of nearest-neighbour cells from the centre cell in order of increasing distance, until we reached a total of 5 million fragments.

##### Defining HSC subpopulations

The scRNA expression data obtained from 10x Multiome was first filtered by removal of the top 5% of highly expressed genes, as well as ribosomal and mitochondrial genes; cells with fewer than 100 RNA counts were removed. We then ran SCTransform (v.0.4.1) with 5,000 variable features to normalize the data. Normalized values were used as input to Spectra^83^ (https://github.com/dpeerlab/spectra). We next ran Spectra initialized with default HSC and global pathways, and including additional gene sets obtained from the published literature; gene sets with fewer than five genes covered were removed. We scored the expression of each Spectra program in each pseudo-bulk. To this end, we used DESeq2 to normalize the pseudo-bulk-by-gene RNA count matrix, and then rescaled per-gene values. For any specific Spectra program, we generated 100 background programs consisting of genes with matched overall expression levels. The average gene expression in the Spectra program of interest was then compared wih that in sampled background programs to derive a *z*-score. Finally, the pseudo-bulk-by-program *z*-score matrix was used to cluster pseudo-bulks into HSC subpopulations.

##### Differential testing

Differential RNA and cCRE accessibility testing was performed using DESeq2 (v.1.42.1)^84^. For each pseudo-bulk, we quantified total RNA read counts for each gene and Tn5 insertion counts in each peak (resized to 1 kb), and used DESeq2 to identify significant differential genes and cCREs, with age as the covariate.

#### Predicting Ets/Runx dimer structures using Alphafold3

For each de novo identified motif representing the Ets/Runx composite motif, we generated the consensus DNA sequence. We then input the same segments of the amino acid sequences of Ets and Runx as those in PDB-4L0Z (Runx, 54–212; Ets, 332–432), along with the consensus DNA sequence and its reverse complements, into Alphafold3. For the structure corresponding to the validated dimer structure, we aligned the AF3-predicted structure to the PDB structure using PyMol (v.2.6) and calculated root mean-squared deviation. All structures were visualized using ChimeraX v.1.8. Solvent-inaccessible bases were identified using the ChimeraX interface function with default parameters.

### Reporting summary

Further information on research design is available in the Nature Portfolio Reporting Summary linked to this article.

## Online content

Any methods, additional references, Nature Portfolio reporting summaries, source data, extended data, supplementary information, acknowledgements, peer review information; details of author contributions and competing interests; and statements of data and code availability are available at 10.1038/s41586-024-08443-4.

## Supplementary information

Supplementary Information This file contains notes, legends for tables and data.

Reporting Summary

Supplementary Tables Supplementary Tables 1–8.

## Data Availability

Raw and processed sequencing data from this study are available on Gene Expression Omnibus (GEO), with accession no. GSE216464. Additional resources, such as pretrained machine learning models and precomputed Tn5 bias tracks, are available at Zenodo (10.5281/zenodo.7121026)^85^. Interactive visualization of human bone marrow and mouse ageing datasets, as well as tracks of seq2PRINT footprint and TF predictions, are available through https://github.com/buenrostrolab/PRINT. Reference genomes for *H. sapiens* (hg38), *M. musculus* (mm10), *D. melanogaster* (dm6), *S. cerevisiae* (sacCer3), *C. elegans* (ce11), *D. rerio* (danRer11) and *P. troglodytes* (panTro6) were accessed at https://hgdownload.soe.ucsc.edu/goldenPath/. SHARE–seq data from GM12878 were obtained from GEO (GSE140203). Chemical mapping of nucleosomes was obtained from GSE97290. ChIP–exo data were sourced from GSE151287. TF ChIP–seq data were obtained from UniBind (https://unibind.uio.no/static/data/20220914/bulk_Robust/Homo_sapiens/damo_hg38_all_TFBS.tar.gz) and ENCODE (full list of accession numbers given in Supplementary Table 8). CTCF degron data were obtained from ENCODE (ENCSR328JGW, ENCSR260SWI). Data on dexamethasone treatment were obtained from ENCODE (full list of accession numbers given in Supplementary Table 8) and EMBL BioStudies (E-MTAB-9910, E-MTAB-9911 and E-MTAB-9912). Data on interferon treatment were obtained from GEO (GSE75306). All resources used in this study can be found in Supplementary Table 8.

## Code availability

The code for Python implementation of PRINT and seq2PRINT can be accessed at https://github.com/buenrostrolab/scPrinter. The code and notebook for reproducing the analysis can be found at https://github.com/buenrostrolab/PRINT. A static version of the code used to generate specific analyses in the Article is available at Figshare (https://figshare.com/s/e173a06b0c62c2f9dd1c)^86^.
