# Supplementary material for: Multiscale footprints reveal the organization of cis-regulatory elements
Source: Nature. 2025 Jan 22;638(8051):779–86. doi: 10.1038/s41586-024-08443-4 (PMC11839466; doi:10.1038/s41586-024-08443-4)

**Multiscale footprints reveal the organization of cis-regulatory elements**

Yan Hu^1,2,7^, Max A. Horlbeck^1,2,3,7^, Ruochi Zhang^1,2,4,7^, Sai Ma^1,2,6^, Rojesh Shrestha^1,2^, Vinay K. Kartha^1,2^, Fabiana M. Duarte^1,2^, Conrad Hock^1,2^, Rachel E. Savage^1,2^, Ajay Labade^1,2^, Heidi Kletzien^2,5^, Alia Meliki^1,2^, Andrew Castillo^1,2^, Neva C. Durand^1^, Eugenio Mattei^1^, Lauren J. Anderson^1^, Tristan Tay^1,2^, Andrew S. Earl^1,2^, Noam Shoresh^1^, Charles B. Epstein^1^, Amy J. Wagers^2,5^, Jason D. Buenrostro^1,2,#^

^1^Gene Regulation Observatory, Broad Institute of MIT and Harvard, Cambridge, MA 02142 USA.

^2^Department of Stem Cell and Regenerative Biology, Harvard University, Cambridge, MA 02138 USA.

^3^Division of Genetics and Genomics, Boston Children’s Hospital, Boston, MA 02115 USA.

^4^Eric and Wendy Schmidt Center, Broad Institute of MIT and Harvard, Cambridge, MA 02142 USA.

^5^Paul F. Glenn Center for the Biology of Aging, Harvard Medical School, Boston MA 02115 USA.

^6^Current address: Department of Genetics and Genomic Sciences, Icahn School of Medicine at Mount Sinai, New York, NY 10029 USA.

^7^These authors contributed equally to this work

^#^Correspondence: [jason_buenrostro@harvard.edu](mailto:jason_buenrostro@harvard.edu)

##

Supplementary information

[**Supplementary Notes 1**](#_3sufn88f16u9)

[Calculation of footprint scores 1](#_l4puy9bvfne4)

[Multiscale footprinting 4](#_z214q1ncljlu)

[Multiple methods to predict TF binding from multiscale footprinting 6](#_iedzn22vvka3)

[Multiscale footprinting vs. V-plot analysis 6](#_ai9n1d4kwxr0)

[Sequence models for TF binding prediction 8](#_v9yjjthr78h9)

[HSC subtypes 9](#_rseulm3lgip0)

[AlphaFold3 Prediction of Runx1/Ets1 interactions 10](#_g693n0yjakro)

[**Supplementary Tables 12**](#_ok9za5eu55jg)

[**Supplementary Data 13**](#_k1ye5sv3ihr6)

## Supplementary Notes

### Calculation of footprint scores

Framework for statistical testing

For each position in the CRE, we define a center footprint region and a flanking region (Fig. 1**e**). We then calculate a footprinting score using statistical testing. The test statistic $\lambda$ is the ratio of total Tn5 insertions in the footprint region divided by the total Tn5 insertions in the footprint and flanking regions combined.

$\lambda= \frac{\sum_{i\in A_{footprint}} x_{i}}{\sum_{i\in A_{flank}} x_{i} + \sum_{i\in A_{footprint}} x_{i}}$

Here,$x_{i}$ is the number of Tn5 insertions at position $i$. $A_{flank}$ and $A_{footprint}$ are the sets of position indices in the flanking and footprint regions, respectively. The goal is to estimate the background distribution of $\lambda$ when no protein is bound, and then compare the observed value of $\lambda$ to its background distribution. If the position of interest is protected from Tn5 insertion by a protein, the observed $\lambda$ should be significantly lower than the background distribution. Hence, we can calculate a *p*-value to represent the significance of such deviation.

We perform two tests on each side (i.e., center-vs-left and center-vs-right) and then keep the less significant *p*-value as the result. More specifically, we calculate the below $\lambda_{left}$ and $\lambda_{right}.$

$\lambda_{left} = \frac{\sum_{i\in A_{footprint}} x_{i}}{\sum_{i\in A_{flankL}} x_{i} + \sum_{i\in A_{footprint}} x_{i}}$, $\lambda_{right} = \frac{\sum_{i\in A_{footprint}} x_{i}}{\sum_{i\in A_{flankR}} x_{i} + \sum_{i\in A_{footprint}} x_{i}}$

$A_{flankL}$ and $A_{flankR}$ are the sets of position indices in the left and right flanking regions, respectively. The reason behind testing each side is to reduce false positive results. Consider the case illustrated below where one accessible CRE is flanked by two nucleosomes:


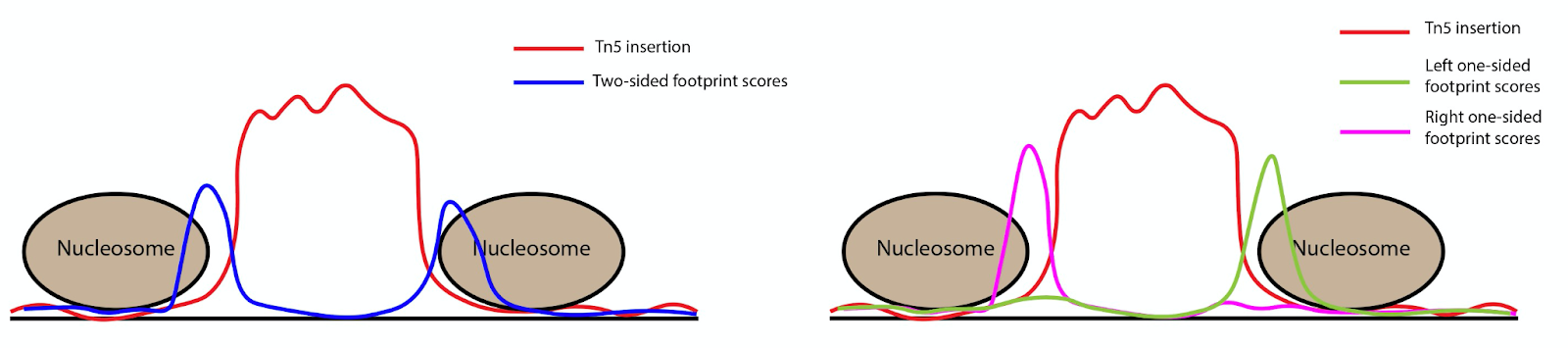


For positions at the edge of the accessible region, Tn5 insertion will be low in the center footprint region as well as one of the two flanking regions. High Tn5 insertion will be observed in the other flanking region. If we compare the footprint region with both flanks combined, we might detect false positive signal since $\lambda$ can still be lower than expected. On the contrary, performing two tests on each side and keeping the less significant result solves such issues.

Modeling the background distribution

BAC DNA Tn5 insertion data was used to estimate the background distribution of $\lambda$. We reason that for each flanking side, the background distribution of $\lambda$ should be determined by center and flanking Tn5 bias, as well as the total number of reads in center and flank. Suppose $b$ is the vector of predicted Tn5 bias (i.e., predicted bias at position $i$ is $b_{i}$), we have

$b_{left} = \sum_{i\in A_{flankL}} b_{i}$, $b_{center} = \sum_{i\in A_{center}} b_{i}$, $b_{left} = \sum_{i\in A_{flankR}} b_{i}$

$c_{left} = \sum_{i\in A_{flankL}\cup A_{center}} x_{i}, c_{right} = \sum_{i\in A_{flankL}\cup A_{center}} x_{i}$

$b_{left}$, $b_{center}$, and $b_{right}$ are total biases in the left flanking, center footprint, and right flanking regions, respectively. $c_{left}$, and $c_{right}$ are coverage for the left and right side testing, respectively. We then aim to model the below distributions

$\lambda_{left}\sim F_{left}(b_{left},b_{center},c_{left})$

$\lambda_{right}\sim F_{right}(b_{right},b_{center},c_{right})$

To this end, the most straightforward approach would be to use the BAC naked DNA data as a lookup table. Suppose we want to compute the footprint score for a specific position (here referred to as “foreground”) in an ATAC-Seq dataset. We compute the center bias, flanking bias, as well as coverage for the foreground observation. Next, we search the BAC naked DNA data to find the k=500 nearest neighbor (KNN) observations in the (center bias, flanking bias, coverage) 3-dimensional space. We compute the $\lambda$ for these background observations and denote as $\lambda_{bg}$. The distribution of $\lambda_{bg}$ is then used as the background distribution for the foreground observed ratio $\lambda_{obs}$ and a *p*-value is computed using *z*-test. To make sure the KNN matching weighs the three features equally, coverage values are first log10-transformed and then all three features are standardized before KNN matching. Additionally, to make sure we cover a wide range of coverage values, the BAC dataset was down-sampled to 100%, 50%, 20%, 10%, 5%, 2%, and 1% and then pooled before KNN matching.

In practice, performing KNN matching for each foreground observation is extremely time-consuming. Therefore, we instead train a neural network dispersion model to learn the below relationship:

($\mu_{left}, {SD}_{left}, \mu_{right}, {SD}_{right})\sim F(b_{left},b_{right},b_{center},c_{left},c_{right})$

We randomly sample 100,000 observations from the BAC dataset. For each of them, we match 500 nearest neighbor observations in the BAC dataset and compute the distribution of $\lambda_{bg}$. Then these 100,000 observations along with their background distribution are used to train the dispersion model.


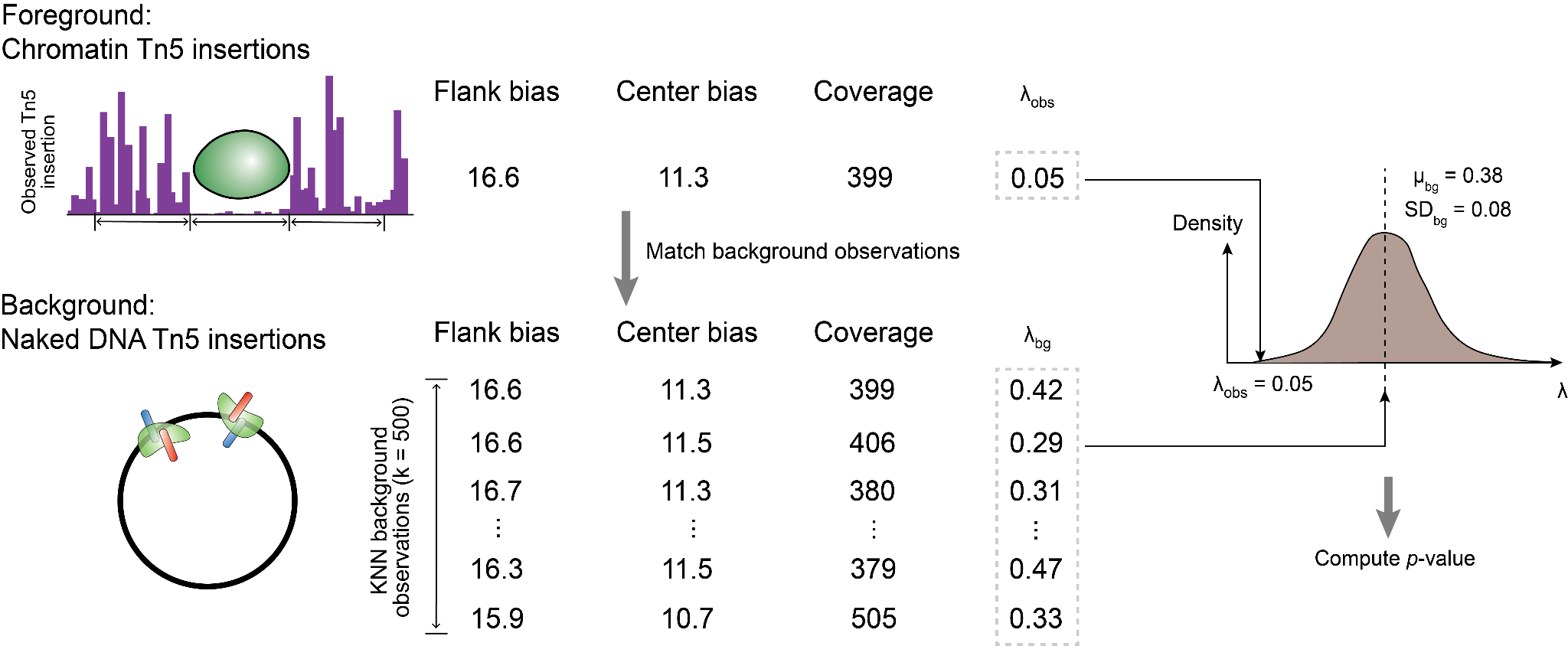


### Multiscale footprinting

There are two main motivations behind using multiscale footprinting for object detection. The first one is that some DNA-binding factors do not leave footprints on their own, potentially due to weak or transient binding. Therefore, we can only infer the binding of such factors through the binding and positioning of nearby objects such as nucleosomes. In these cases, using only footprints detected at the scale corresponding to the size of the object itself will lead to false negative results (e.g., only using the 40 bp scale footprint to detect YY1 binding).

The second motivation is to filter signal bleed through across scales. We realized that the footprint signal of an object can bleed through into lower scales, potentially due to non-linear impact on Tn5 insertion by object binding. For instance, examine the example below：


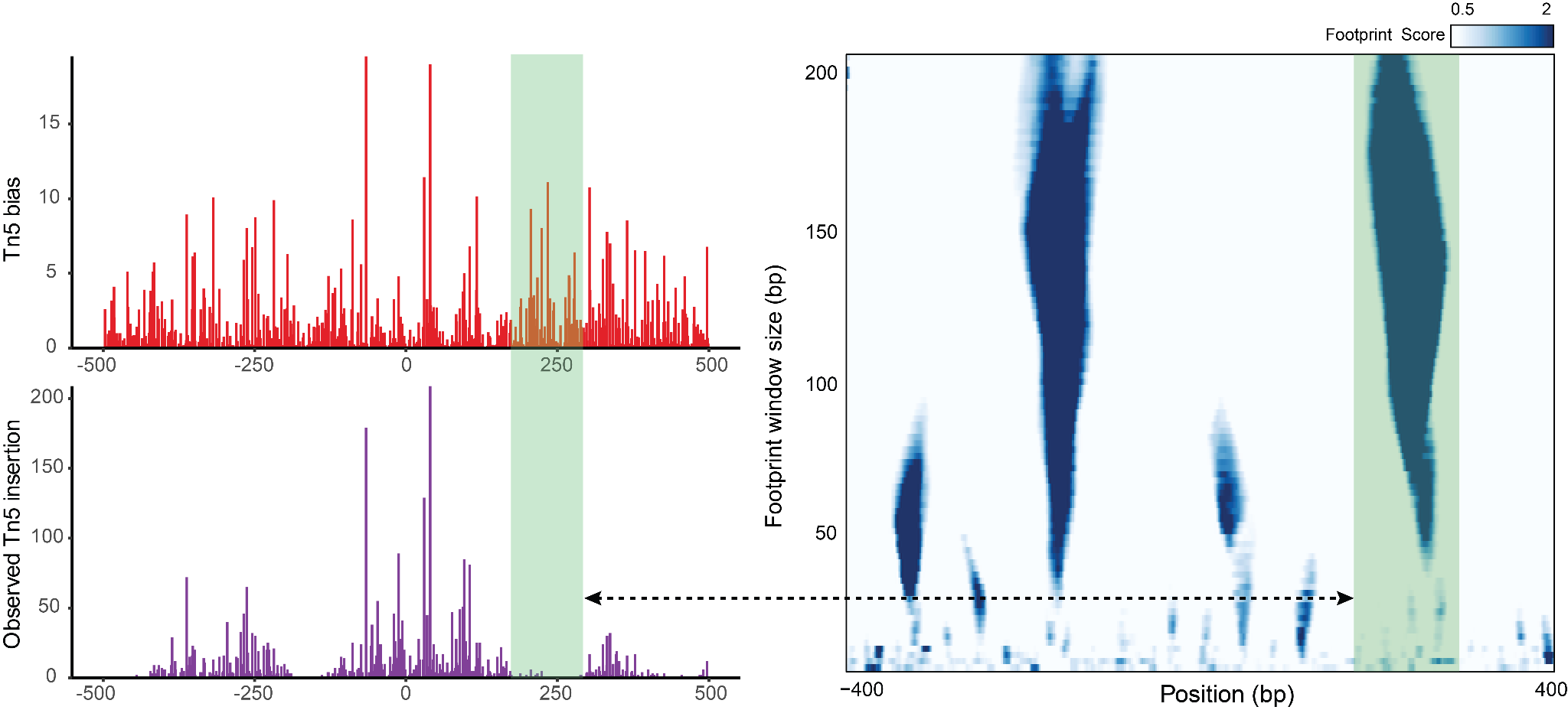


The highlighted boxes in the left and right plots represent the same genomic region. We can see that this region shows footprint signals from around 40 up to 200 bp scales, representing a bound nucleosome. Although the nucleosome-bound region is around 140 bp in diameter, the footprint signal is not limited to 140 bp scale, but bleeds through to a much lower spatial scale. In the case shown above, this is because nucleosome binding does not affect Tn5 insertion at all positions equally. Positions with very high Tn5 bias, shown as “spikes” in the Tn5 bias track, are affected most dramatically. In the highlighted region, several positions have Tn5 bias spikes, and are thus expected by the model to have a high center / (center + flank) ratio of Tn5 insertion. However, in the observed insertion track, due to the binding of the nucleosome, Tn5 insertion at these locations are suppressed to a low baseline level, similar to flanking regions. Therefore, the observed center / (center + flank) ratio is lower than the expected, and the model will return significant footprint signals at such positions. In essence, the above results from the unequal impact of the bound object on Tn5 insertion. In other words, if insertion at all base pairs in the bound regions are reduced by the same scaling factor, including positions with Tn5 bias spikes, then most of the bleed-through will no longer appear.

As a result, if we detect TF binding only by examining footprint signals at the scale matching the size of TFs, such bleed-through will result in false positive signals. The bleed-through effect is theoretically not specific to our footprinting method. For any method that defines a footprint window and calculates deviation of observed Tn5 insertion in the footprint window from the expected level, this could be a potential source of false positive signal. Positions with Tn5 bias spikes but bound by nucleosomes will tend to have high expected but low observed cutting, and could show up as a false positive footprint at the scale of TFs.

Given the above, we realized that trying to detect objects using footprint scores at a single scale and a single location is under-powered. We instead leverage footprint signals across scales and positions for accurate object detection.

### Multiple methods to predict TF binding from multiscale footprinting

Throughout the development of PRINT and seq2PRINT, we have explored a few different ways to predict TF binding, each with their strengths and limitations. Overall, they fall into two categories: (1) Footprint-to-TF prediction, which uses local multiscale footprints as model input, and predicts whether a motif site is bound by the TF or not. (2) seq2PRINT, which first trains a model that uses DNA sequence to predict multiscale footprints. We then use the sequence attribution scores derived from the model to predict TF binding.

In terms of predictive power, seq2PRINT is superior in both accuracy and resolution (as it can score each single base pair on their contribution to footprint prediction). Hence, we recommend using seq2PRINT when there are adequate computational resources. However, seq2PRINT requires training a new convolutional neural network for each new dataset and computing sequence attribution scores, both of which are relatively resource-intensive. Footprint-to-TF prediction, which we described in early versions of this manuscript including our pre-print (<https://www.ncbi.nlm.nih.gov/pmc/articles/PMC10081223/>), uses a single light-weight model for all datasets and runs significantly faster at the cost of moderate precision loss. We provide code and a pre-trained footprint-to-TF model in addition to seq2PRINT on our Github page to allow users to use the appropriate model for their use case.

### Multiscale footprinting vs. V-plot analysis

V-plot analysis is an alternative method for representing DNA-protein interactions across spatial scales that has been applied to DNAse-, MNase-, and ATAC-seq. However, there are a few key differences between multiscale footprinting and V-plot methods

(1) V-plot methods mostly examine the position-fragment size relationship and often do not account for sequence biases. For assays such as ATAC-seq where sequence bias is very strong, the V-plot will be heavily confounded especially for the detection of TFs. For example, we visualized the V-plot for an example region in our naked DNA dataset (see figure below). Even though there is no protein bound, the V-plot shows patterns resembling TF binding patterns such as those shown in **Figure 4B** of the original V-plot paper (PMID:22025700).


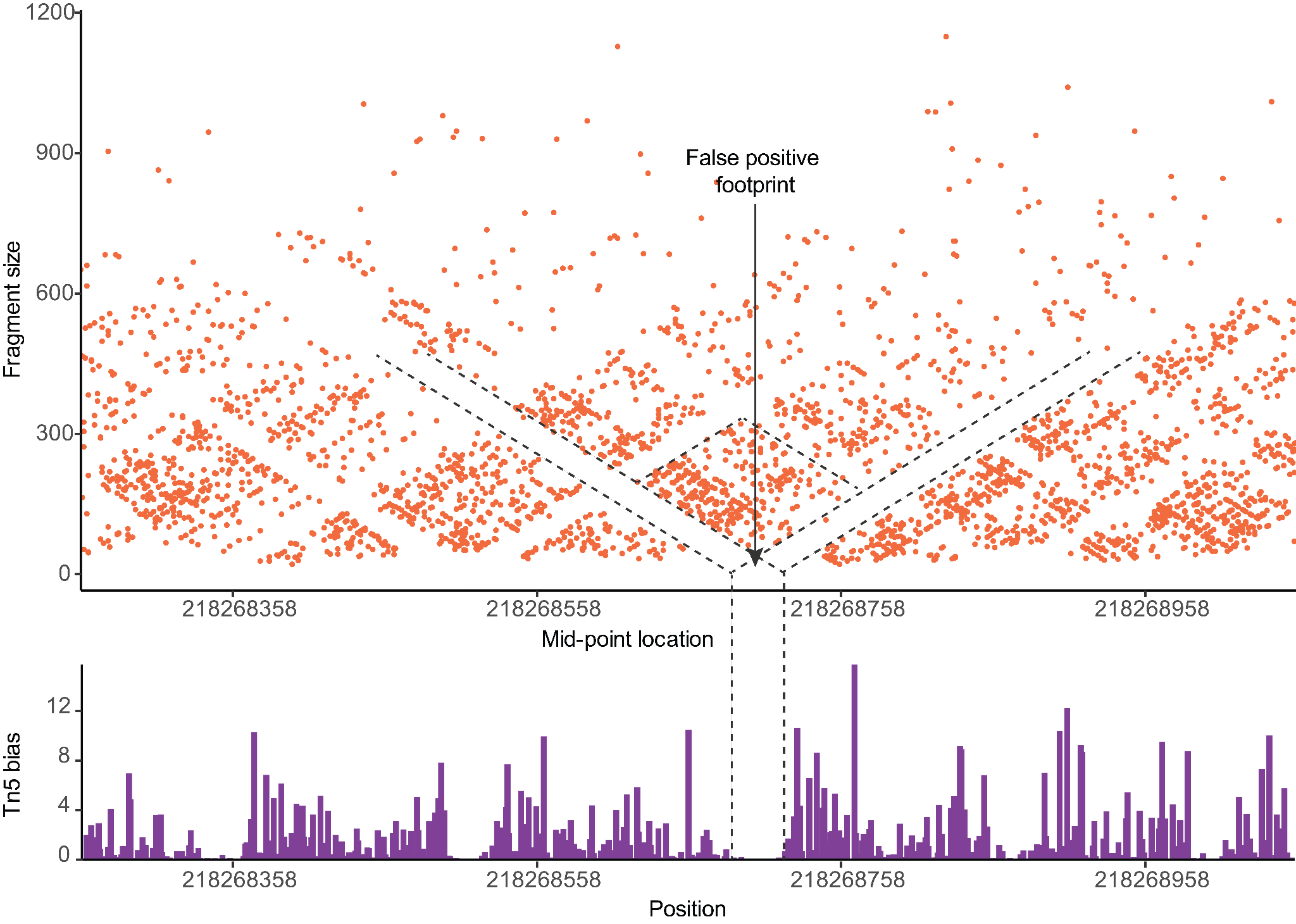


(2) Even with completely accurate bias correction, it is equally important to model background dispersion of Tn5 insertion due to random noise. When data is sparse and noisy such as pseudo-bulked scATAC-seq data, it is crucial to be able to distinguish background noise and events that are statistically significant particularly when evaluating single loci (as opposed to aggregating across motifs).

(3) Multiscale footprinting provides a feature map that is amenable to downstream statistical or deep-learning analysis as it separates the footprints of different objects along the x and y axes. By comparison, in V-plots the footprints of nearby objects can be convoluted by overlapping V patterns. Discrete footprints enable analyses where we examine seq2PRINT attribution scores for individual footprints. It also allows sub-sampling of the multiscale pattern at representative scales to enable signal filtering and reduce the computational load for machine learning tasks.

(4) A particular strength of V-plot analysis is the use of fragment size to further define regions protected from nuclease/transposase activity. During the initial development of PRINT, we explored using this feature and did not find it enhanced the accuracy of footprint detection. However, it remains a possible additional feature in future versions, particularly as a parameter in seq2PRINT-like deep learning.

### Sequence models for TF binding prediction

Throughout the development of seq2PRINT, we have found several improvements that enhanced TF binding prediction accuracy:

First of all, we noticed that some true TF binding sites are captured by the model but have strong negative attribution scores. This suggests that using the raw attribution scores directly was not the optimal way to rank TF motif sites for binding prediction. The attribution-TF binding relationship is also not necessarily linear. We therefore trained a lightweight neural network that takes the attribution scores in a local window to predict the TF binding at any specific base-pair position, and we adopted this TF binding model as the final seq2PRINT TF binding predictor. We observed that the relationship between the sequence attribution score and TF binding score at the same base pair has an asymmetric distribution with respect to the origin, as can be seen in the figure below:


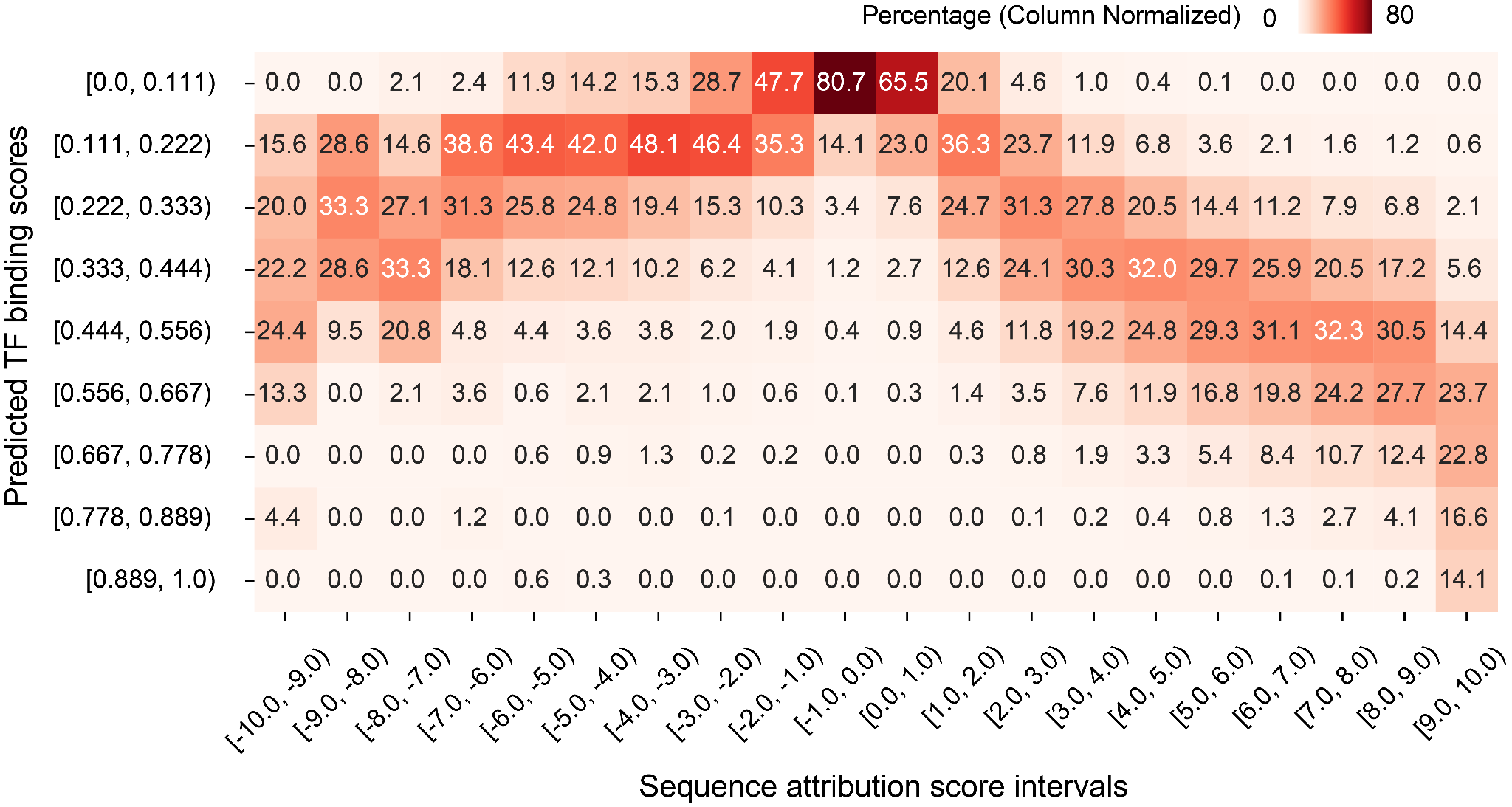


Additionally, compared to previous methods which trained on accessibility magnitude and shape, such as ChromBPNet, using multiscale footprint also seemed to enable the model to learn additional information and improve accuracy. Note that for seq2PRINT, the training is only dependent on the footprint head, although sequence attribution scores can be calculated based on either the footprint head, the count head, or both. The performance of different model versions can be seen in the figure below:


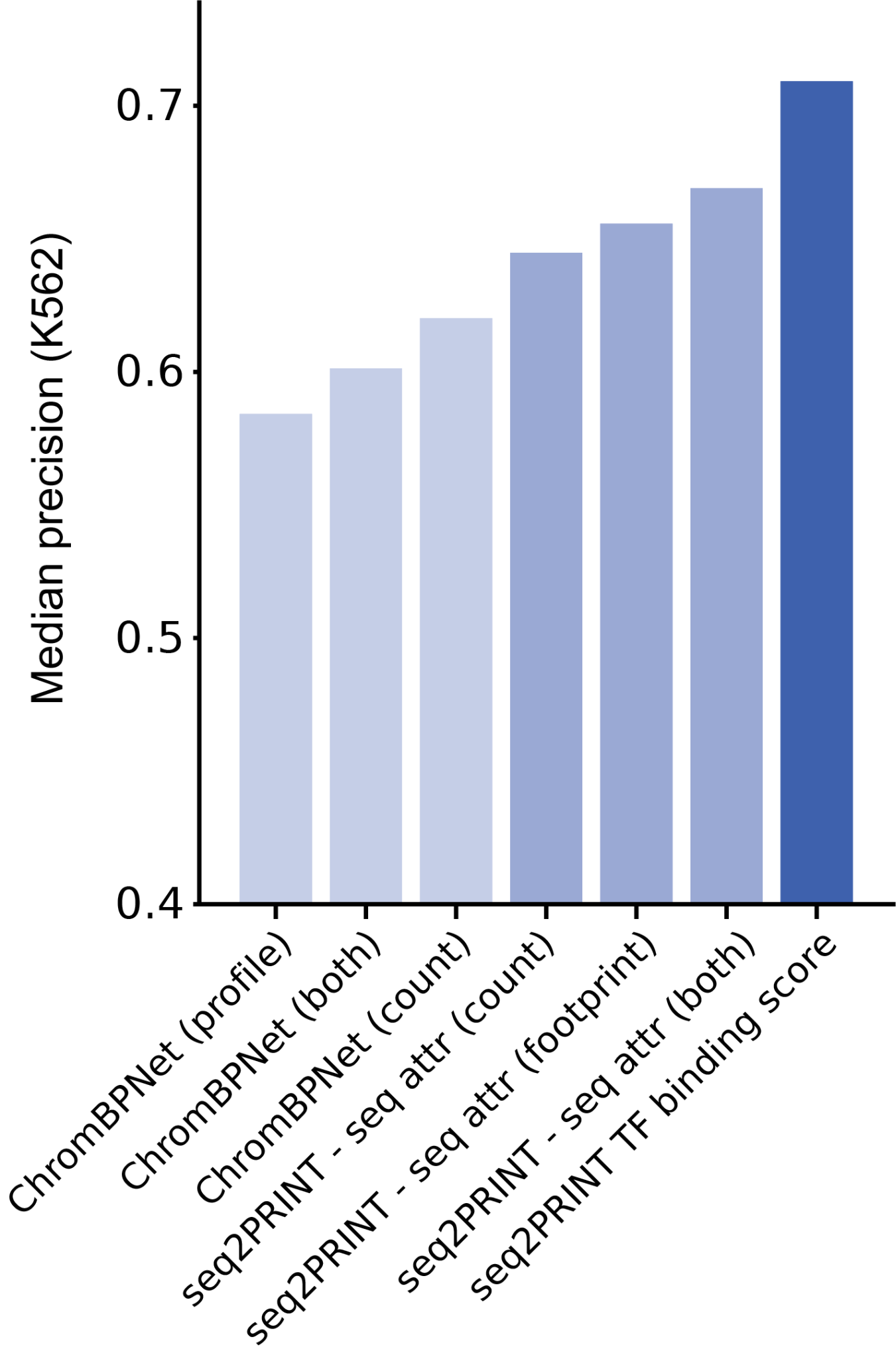


### HSC subtypes

For the mouse HSC aging analysis, we examined whether the previously reported HSC subtypes can be recapitulated by our dataset.

We first defined representative cell states using the ATAC data. To do this, we used SEACells (Persad et al. Nature Biotech. 2023) which uses a graph embedding to define maximally distinctive states. SEACells was designed to achieve a good balance between signal detection and cell state resolution. We chose 100 meta-cells as it samples the diversity of the data set without creating overwhelming computational complexity.

We next labeled meta-cells by taking each meta-cell and aggregating the transcriptome of similar cells to compute a pseudo-bulk. We obtained gene signatures curated using Spectra (Kunes et al. Nature Biotech. 2023) and published HSC signatures and scored meta-cells using the average expression of these gene signatures. Using these gene signatures as features, we then used hierarchical clustering and identified 5 meta-cell clusters reflecting lineage bias and age (see **Fig. 4e-f**). We labeled each pseudobulk based on their cluster identity, but also retained the independent pseudobulks for unbiased seq2PRINT analysis.

Previous studies such as Rodriguez-Fraticelli et al., 2020 (PMID: 32669716) and Pei et al., 2020 (PMID: 32783885) reported the existence of two major HSC subtypes: (i) one with low lineage output, more megakaryocyte-biased, and expressing higher levels of quiescence and self-renewal markers (i.e., low-output or Mk-biased subtype), and (ii) one with higher multilineage output (i.e., multilineage subtype). We scored our mouse aging HSC dataset with gene signatures obtained from both studies and confirmed the existence of similar HSC subtypes. Interestingly, we also observed a general increase in megakaryocyte bias during aging.


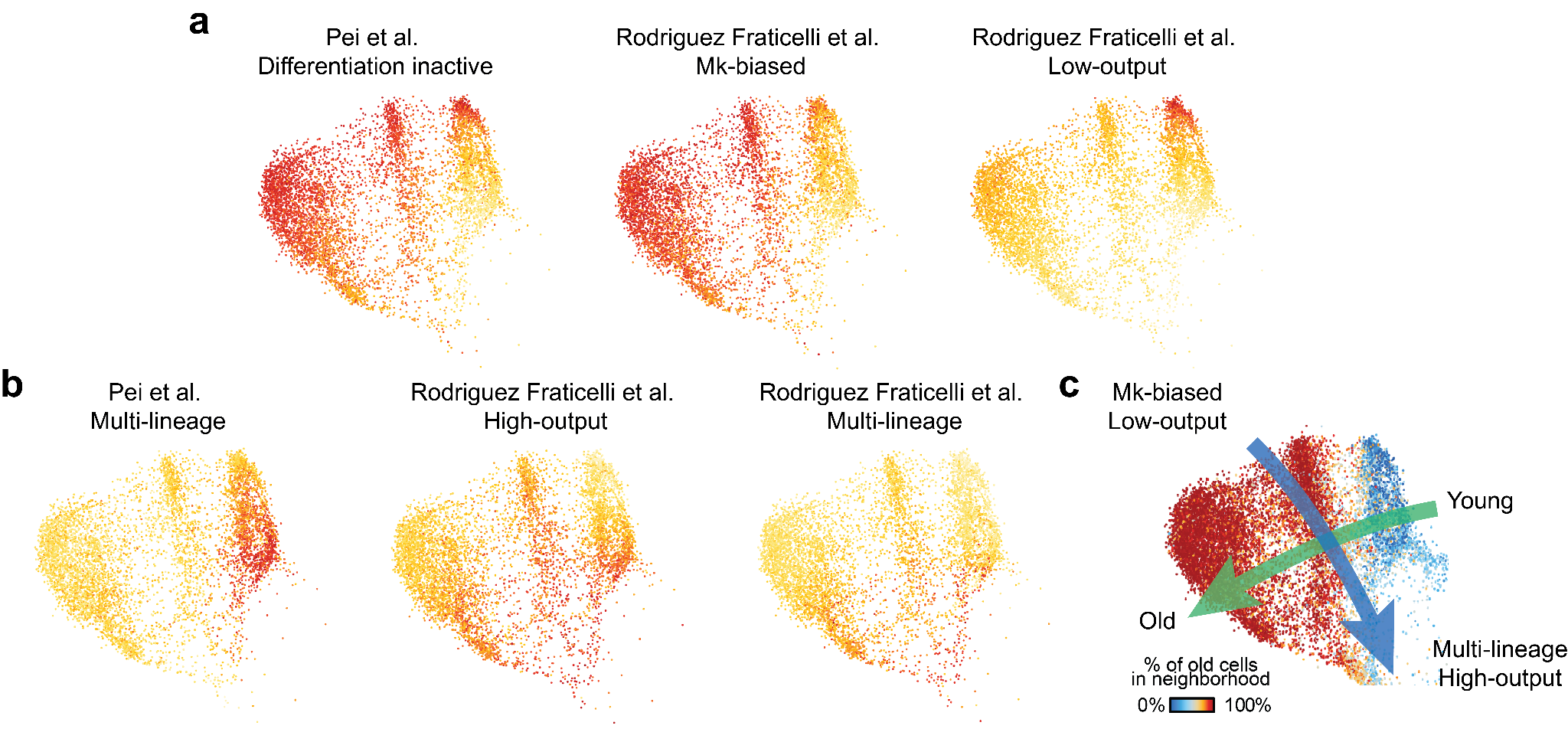


### AlphaFold3 Prediction of Runx1/Ets1 interactions

We generated AlphaFold3 predictions for the Ets dimer predictions. Overlap of the predicted structure (blue) and crystal structure (red) aligned in Pymol and visualized in Mol* Viewer showed (RMSD= 0.787 Å):


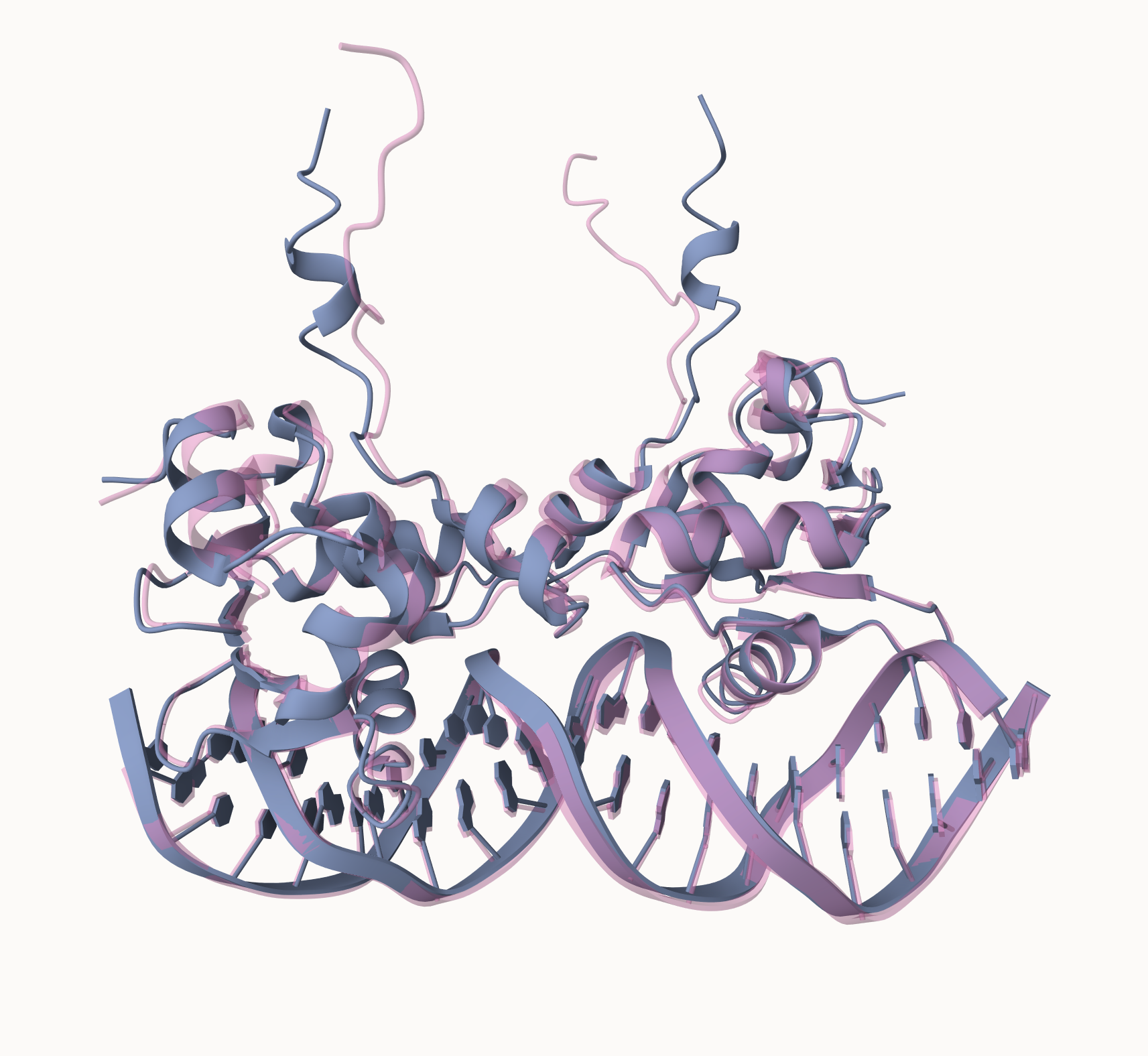


We also generated AlphaFold3 predictions for all *de novo* identified composite motifs with Runx and Ets predictions (detailed in Methods). Motif #10 matches the Runx1/Ets1 binding configuration resolved in PDB 4L0Z. Overlap of the predicted structure (blue) and crystal structure (red) aligned in Pymol and visualized in Mol* Viewer showed (RMSD=0.825 Å) :


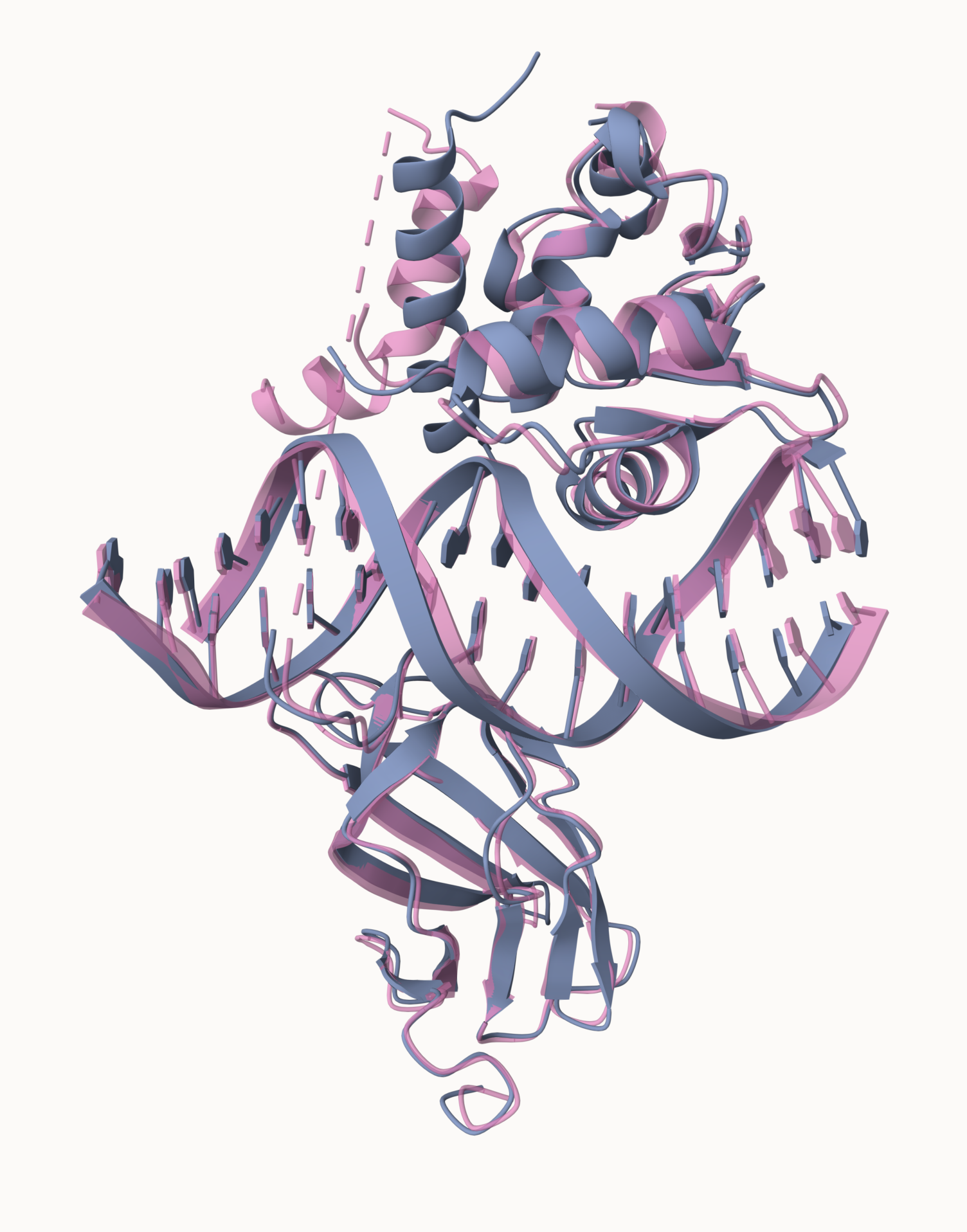


Representative structures are included in **Extended Data Fig. S9h**; all predicted structures for these motifs are included below colored as in the supplementary figure.


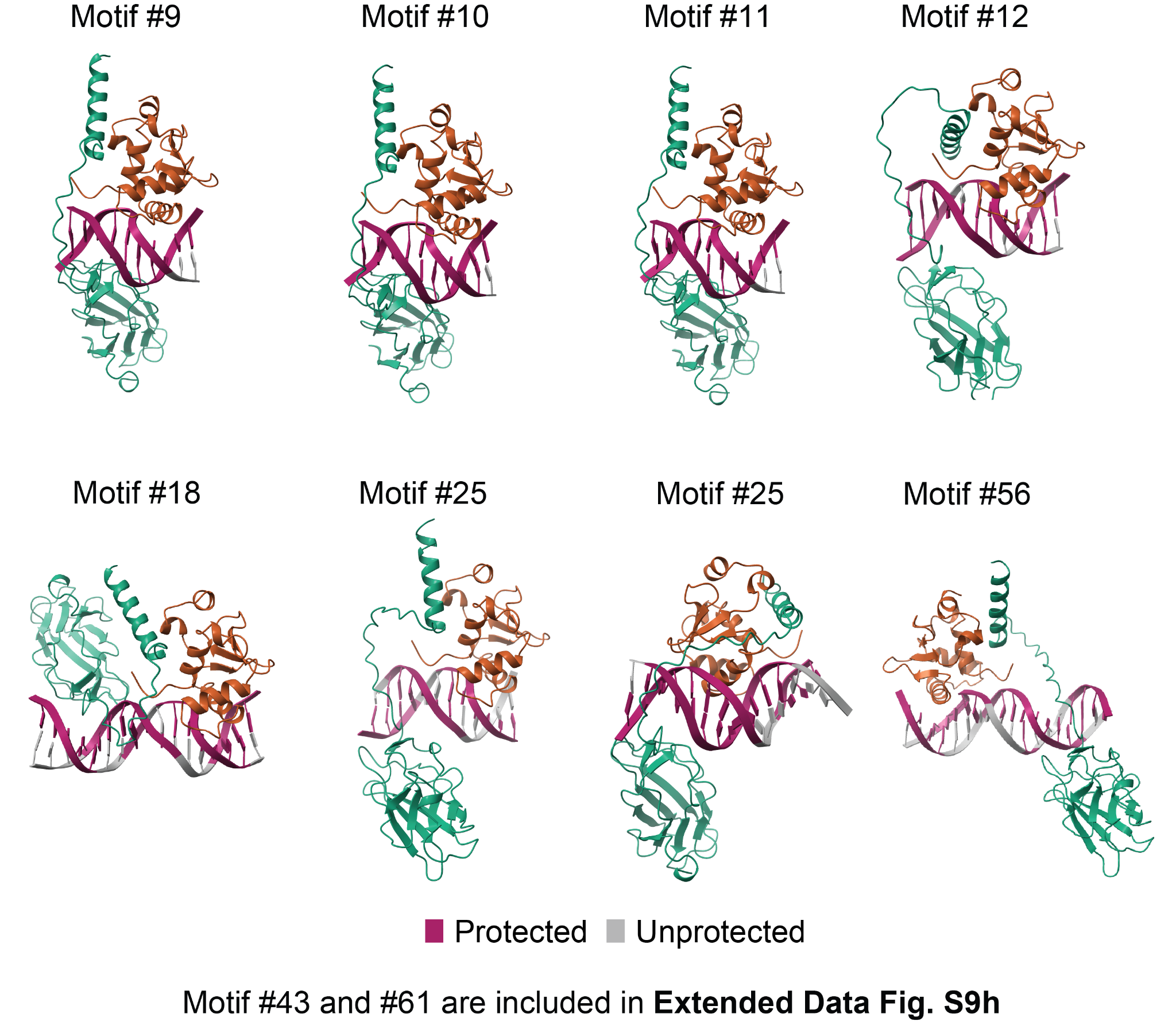


## Supplementary Tables

**Supplementary Table 1. BACs used in this study.**

Summary table of all BACs included in the study. Contains BAC IDs and genomic coordinates.

**Supplementary Table 2. TF binding prediction benchmark.**

Summary table of TF binding prediction benchmark. Contains precision at top 10% high scoring sites by each method and AUPRC in K562. Also contains precision and recall tested on representative bone marrow cell types.

**Supplementary Table 3. TF binding dynamics during hematopoiesis.**

TF binding dynamics predicted by seq2PRINT during erythroid and B cell differentiation. Corresponds to results in **Fig. 3f** and **Extended Data Fig. S6s**. Contains the below columns. CRE-lag: time-lag between TF binding and gain of cCRE accessibility. RNA-lag: time-lag between TF binding and gain of RNA level. Postive lag values mean that the inferred TF binding score at the motif sites of this TF rises prior to opening/widening of the cCRE or expression of the RNA and vice versa. Distance: Average distance of TF binding site to cCRE centers.

**Supplementary Table 4. Differential gene expression in mouse HSC aging.**

Table of differential expression of genes during HSC aging. Results were derived from DESeq2 with a two-sided Wald test by comparing young and old HSC pseudo-bulks.

**Supplementary Table 5. Spectra programs and TF motif scores across HSC pseudobulks.**

Summary table of Spectra gene programs and TF changes during mouse HSC aging. Contains the below tabs. Spectra program scores: Spectra program expression levels normalized as z-scores in each HSC pseudo-bulk. Spectra gene programs: Lists of genes included in each Spectra gene program. HSC pseudo-bulk clustering: k-means cluster labels of HSC pseudo-bulks. Pseudo-bulk center barcodes: Barcodes of the center cells for each pseudo-bulk. TF score - de novo motifs: chromVAR motif scores of each *de novo* TF in each HSC pseudo-bulk. TF score - cisBP motifs: chromVAR motif scores of each cisBP TF in each HSC pseudo-bulk.

**Supplementary Table 6. TF and nucleosome changes during HSC aging**

Summary table of differential TF and nucleosome testing across aging. Contains the below tabs. de novo motifs aging test: two-tailed t-test results of chromVAR motif scores using *de novo* motifs learned by seq2PRINT. cisBP motifs aging test: two-tailed t-test results of cisBP motif scores. Enrichment at lost nucleosomes: motif enrichment at regions with age-associated loss of nucleosome footprints. Motif bagging: grouping of motifs and representative motifs within each group.

**Supplementary Table 7. Runx-Ets interaction at composite motifs.**

Summary table of different Runx-Ets or Ets-Ets dimer structures at *de novo* composite motifs learned by seq2PRINT. For publicly available PDB structures, the PDB accession ID is provided. For AlphaFold3 predictions, the motif ID (same as the “ID” column in **Supplementary Table 6**), the DNA sequence, as well as the TF protein sequence used as input for AlphaFold3 are provided.

**Supplementary Table 8. Resources used in this study.**

Summary table of different datasets and resources used in this study. Including links to code and packages as well as accession numbers of external datasets.

## Supplementary Data

**Supplementary Data 1.** De novo motifs identified by TF-MoDISco in HepG2.


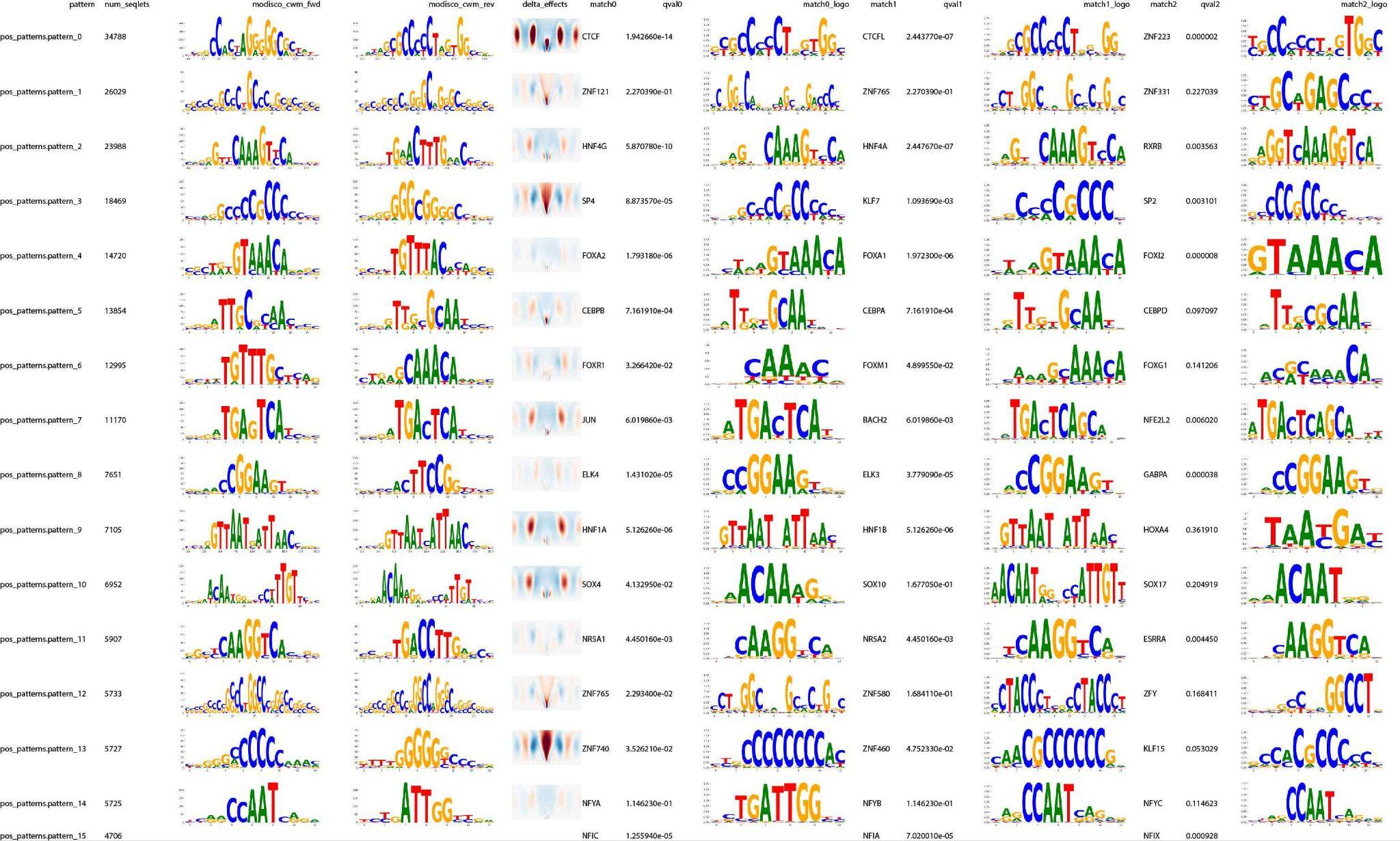

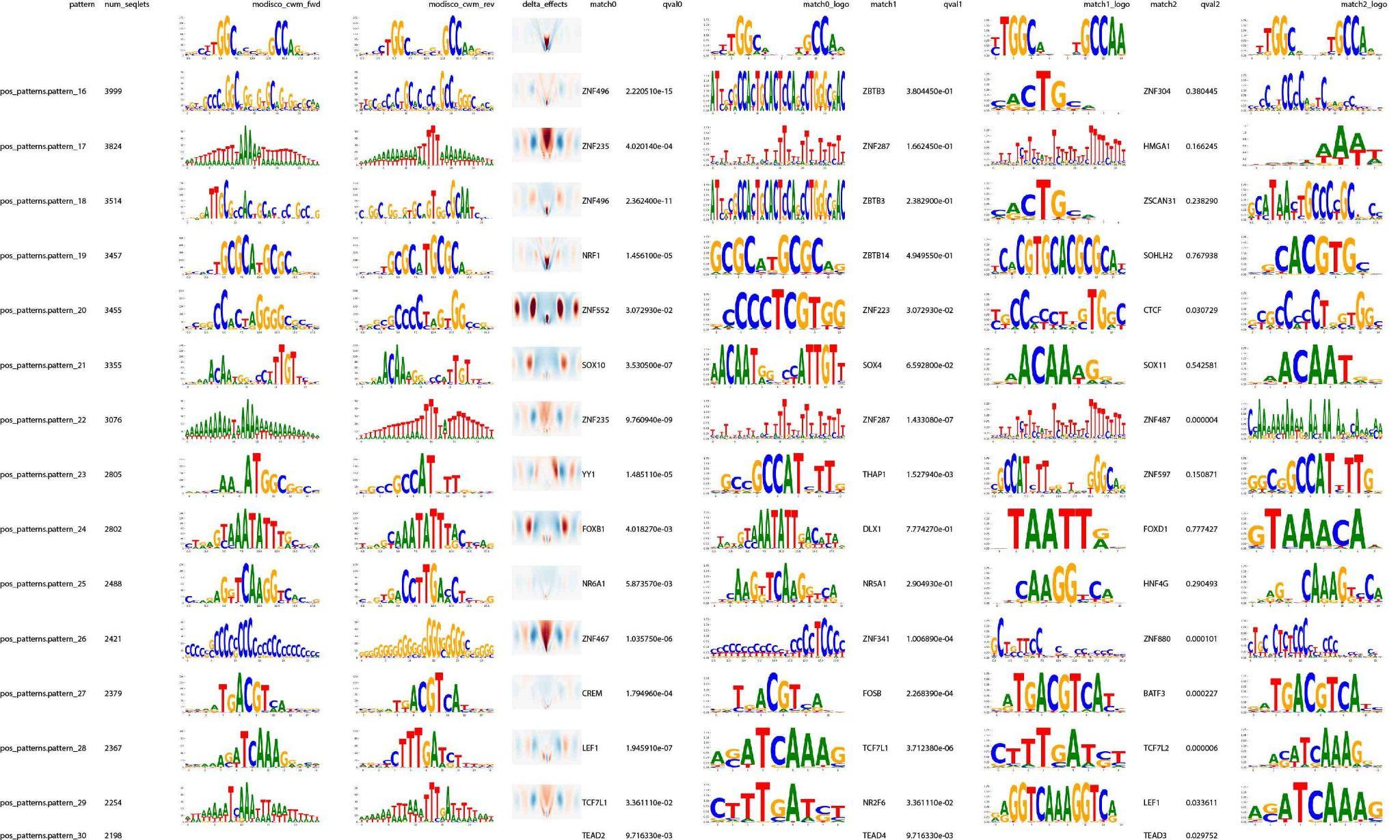


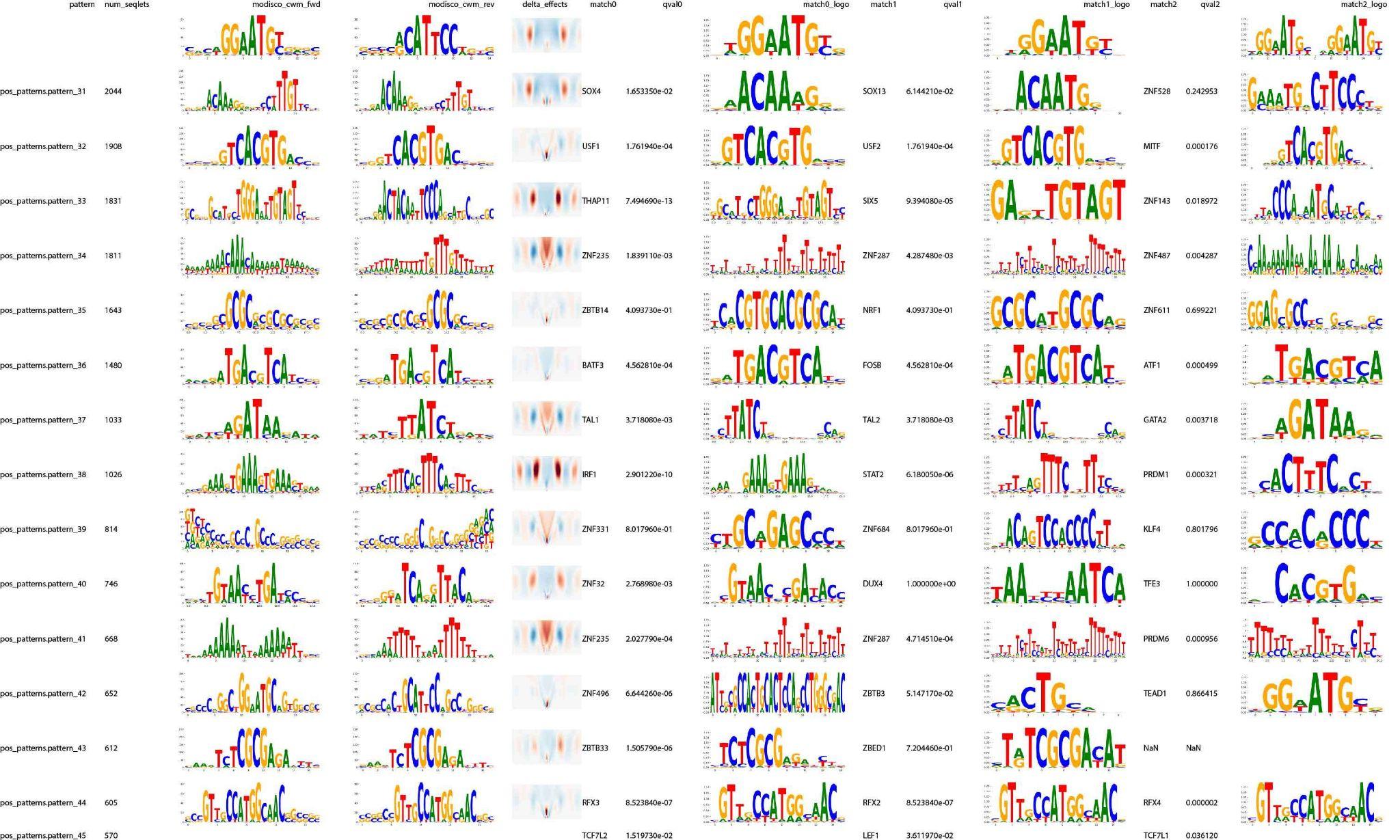

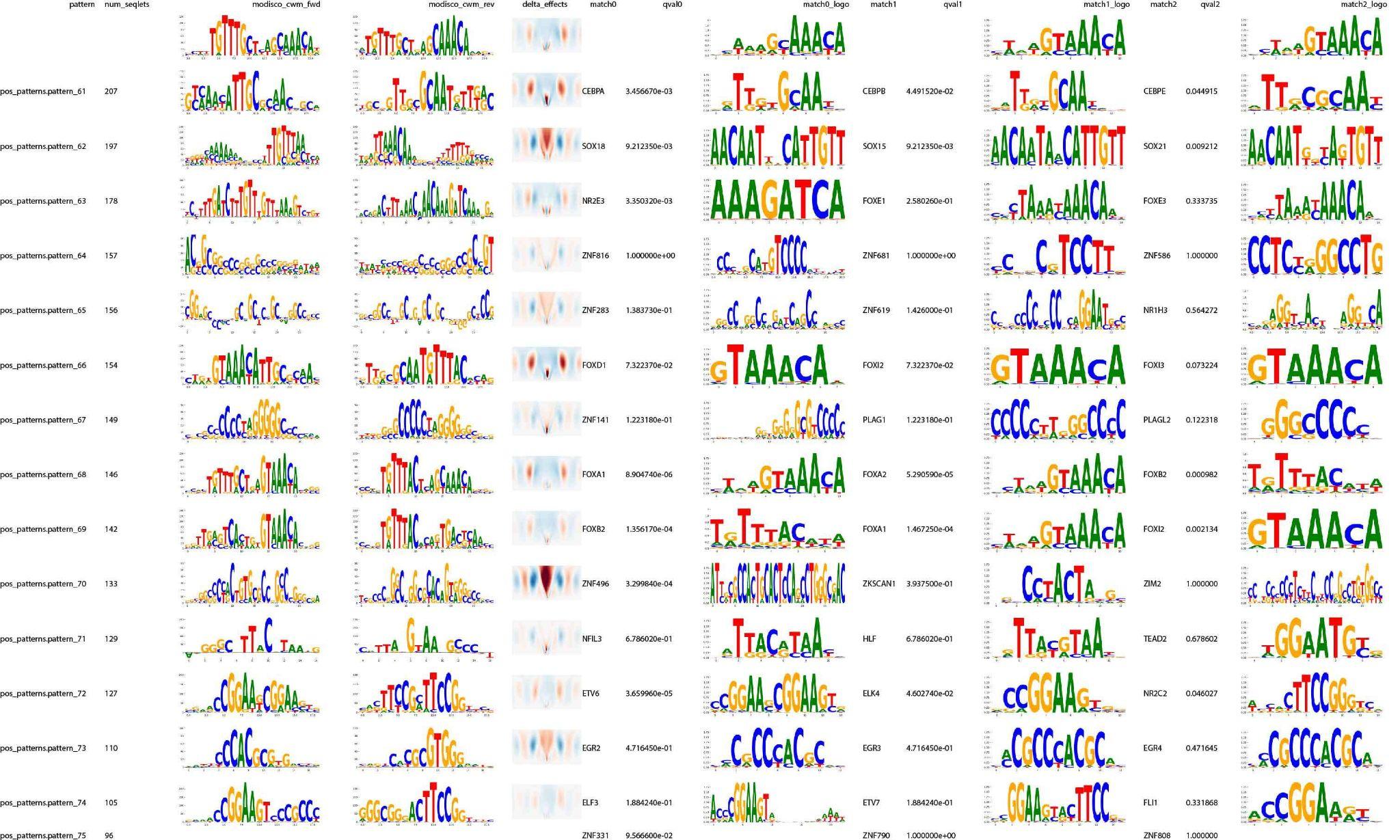

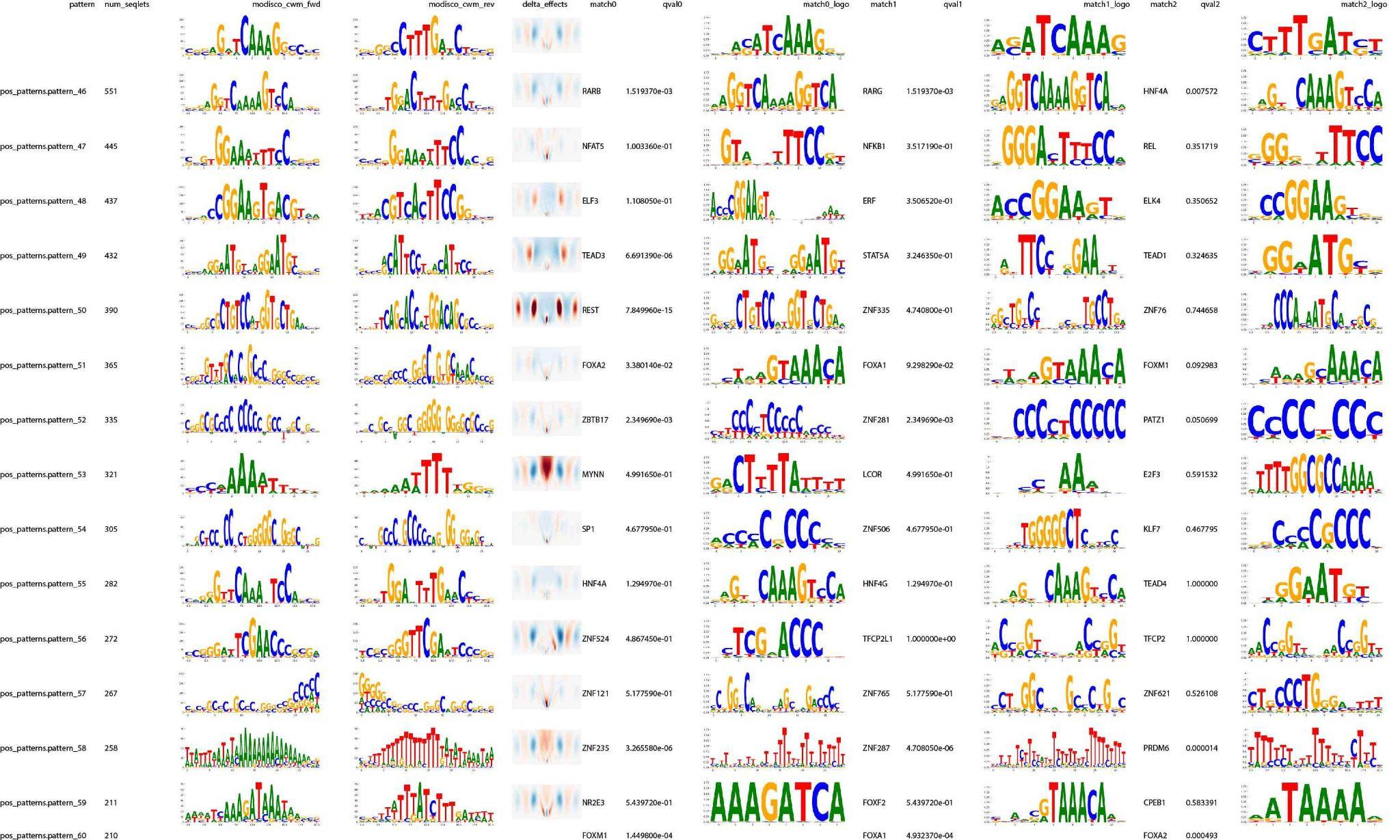


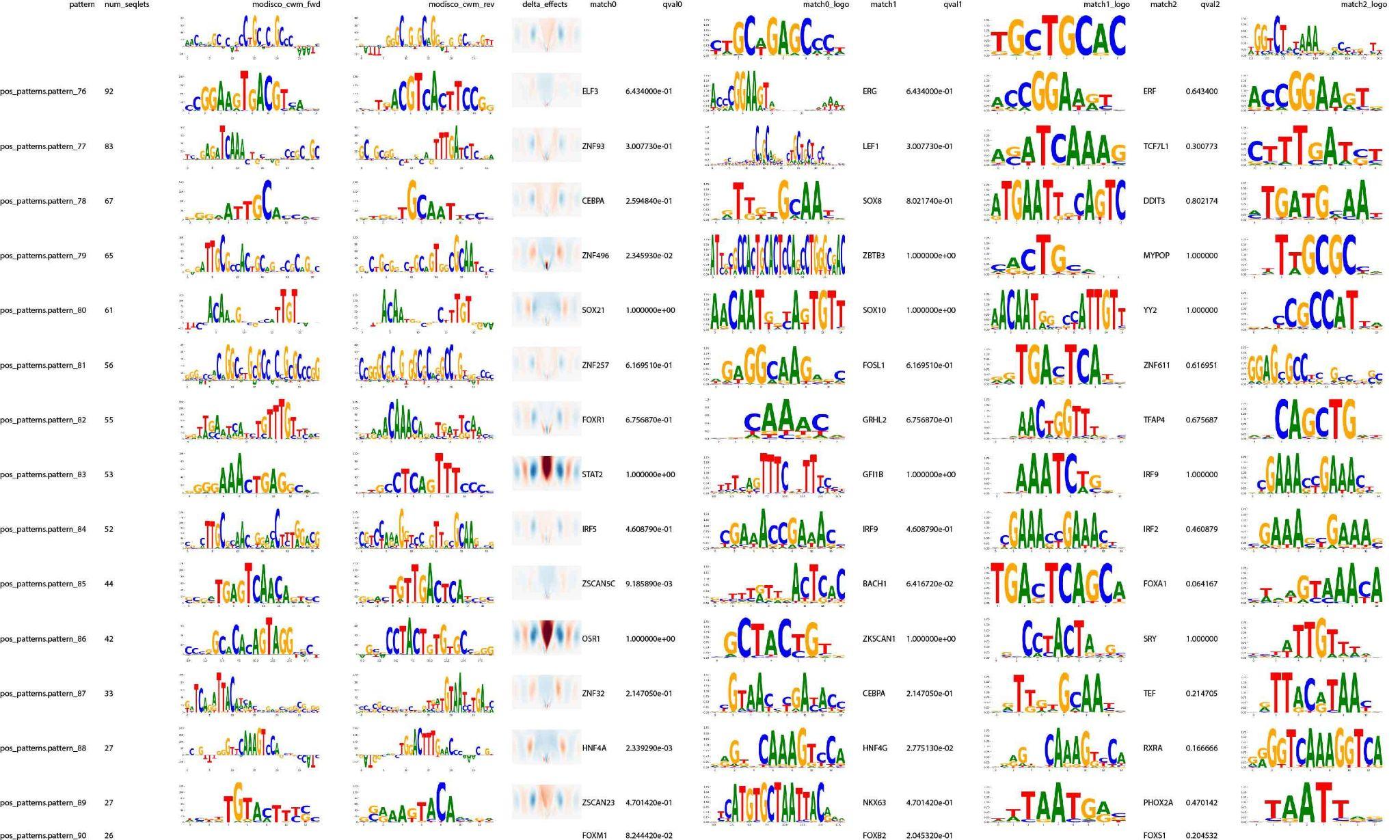


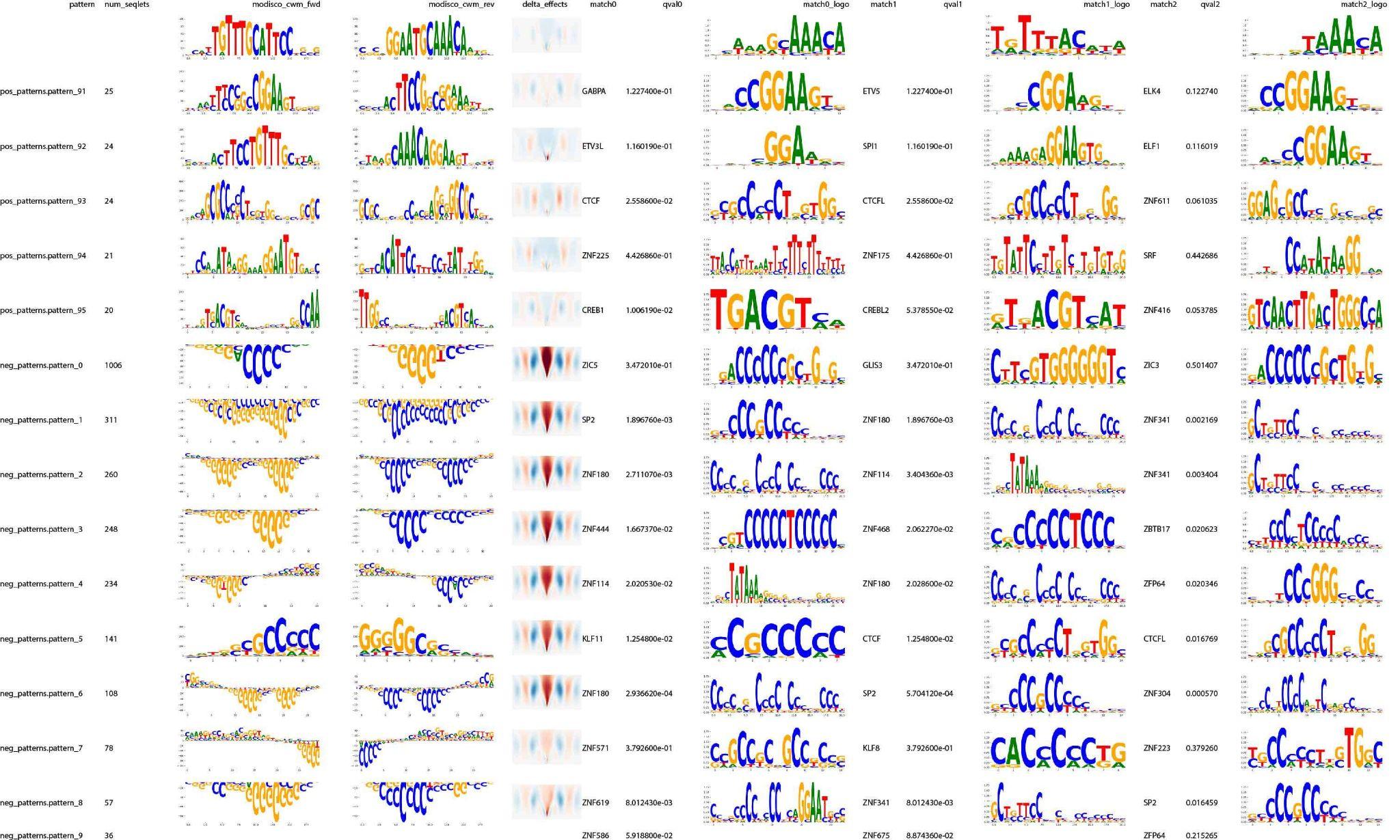


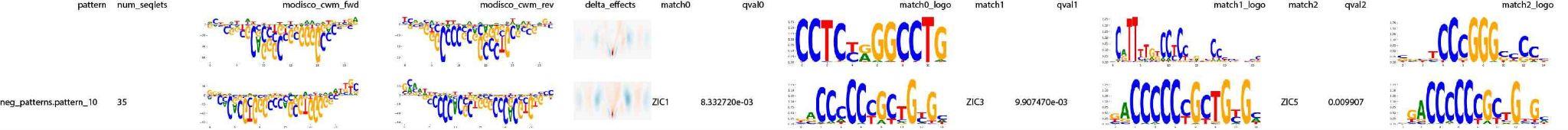


**Supplementary Data 2.** De novo motifs identified by TF-MoDISco in mouse aging HSCs. Details on motifs can be found in **Supplementary Table 6**.


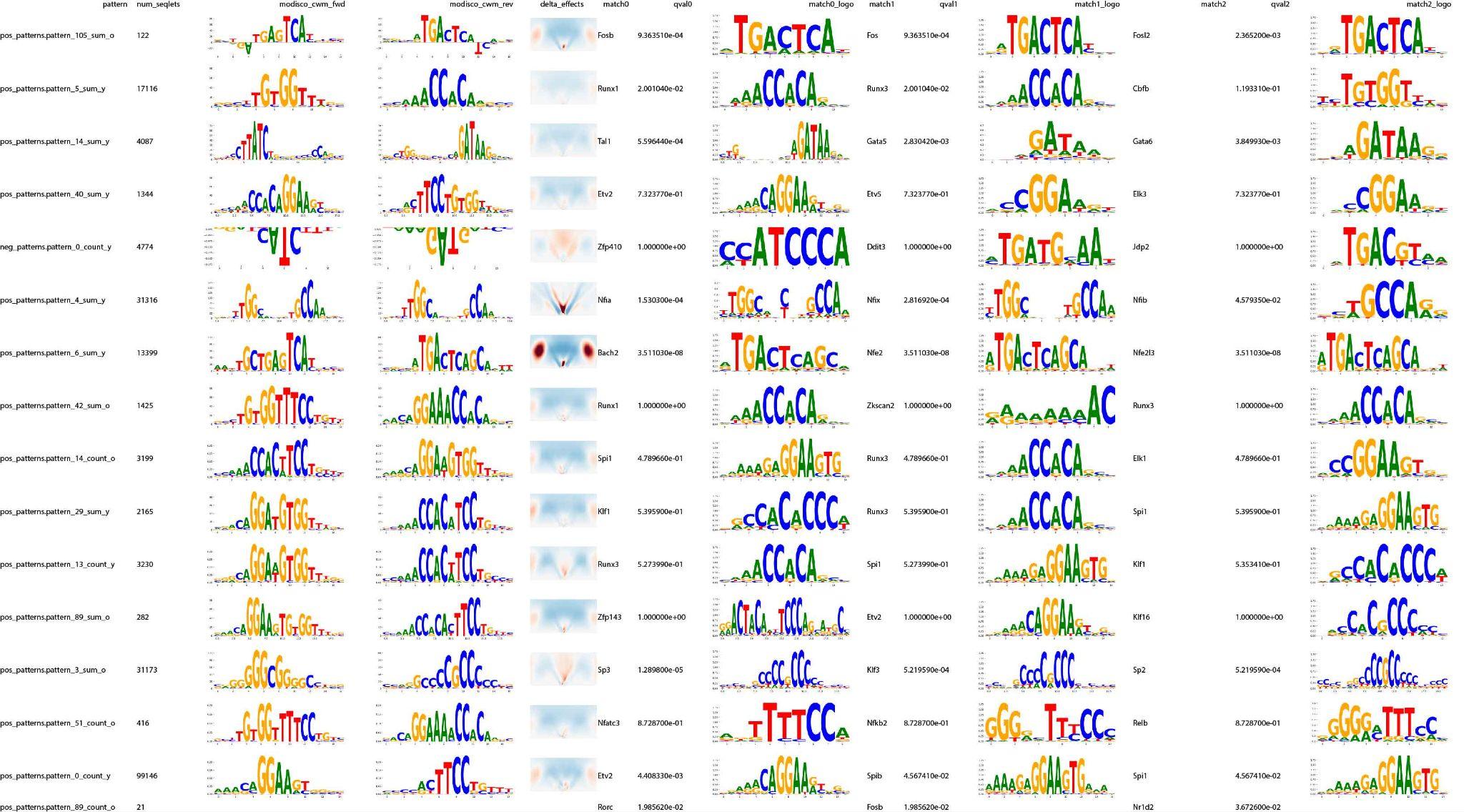

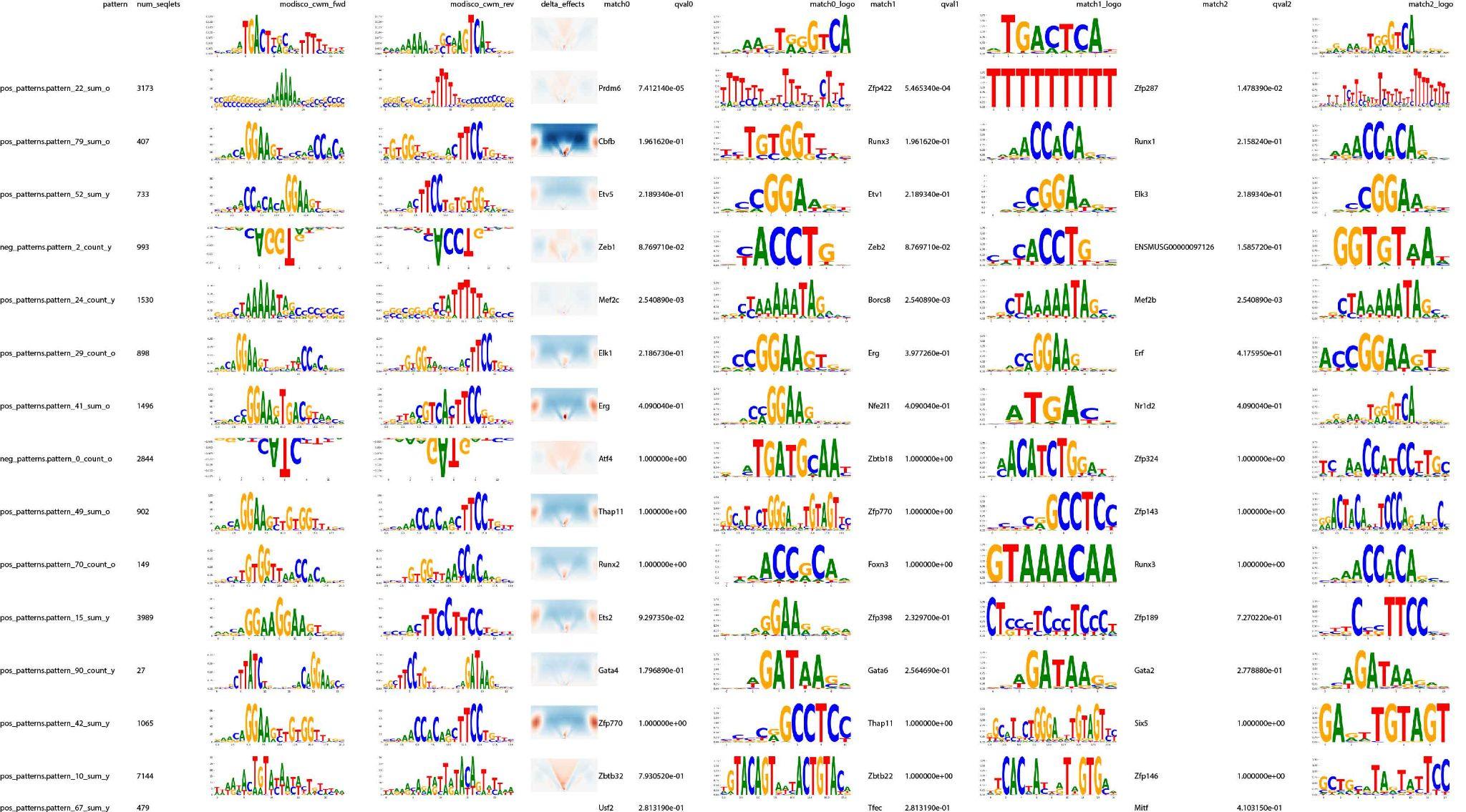


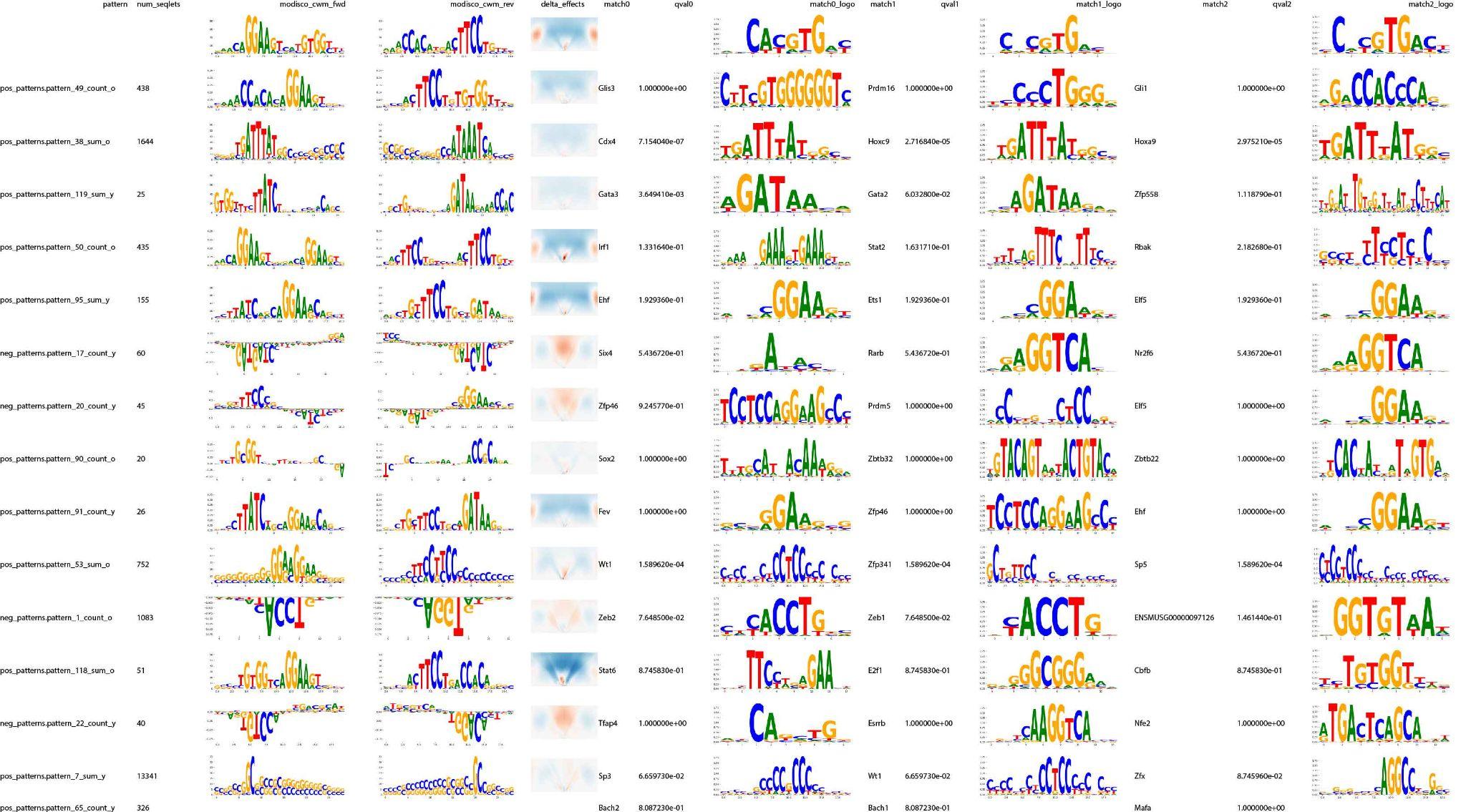

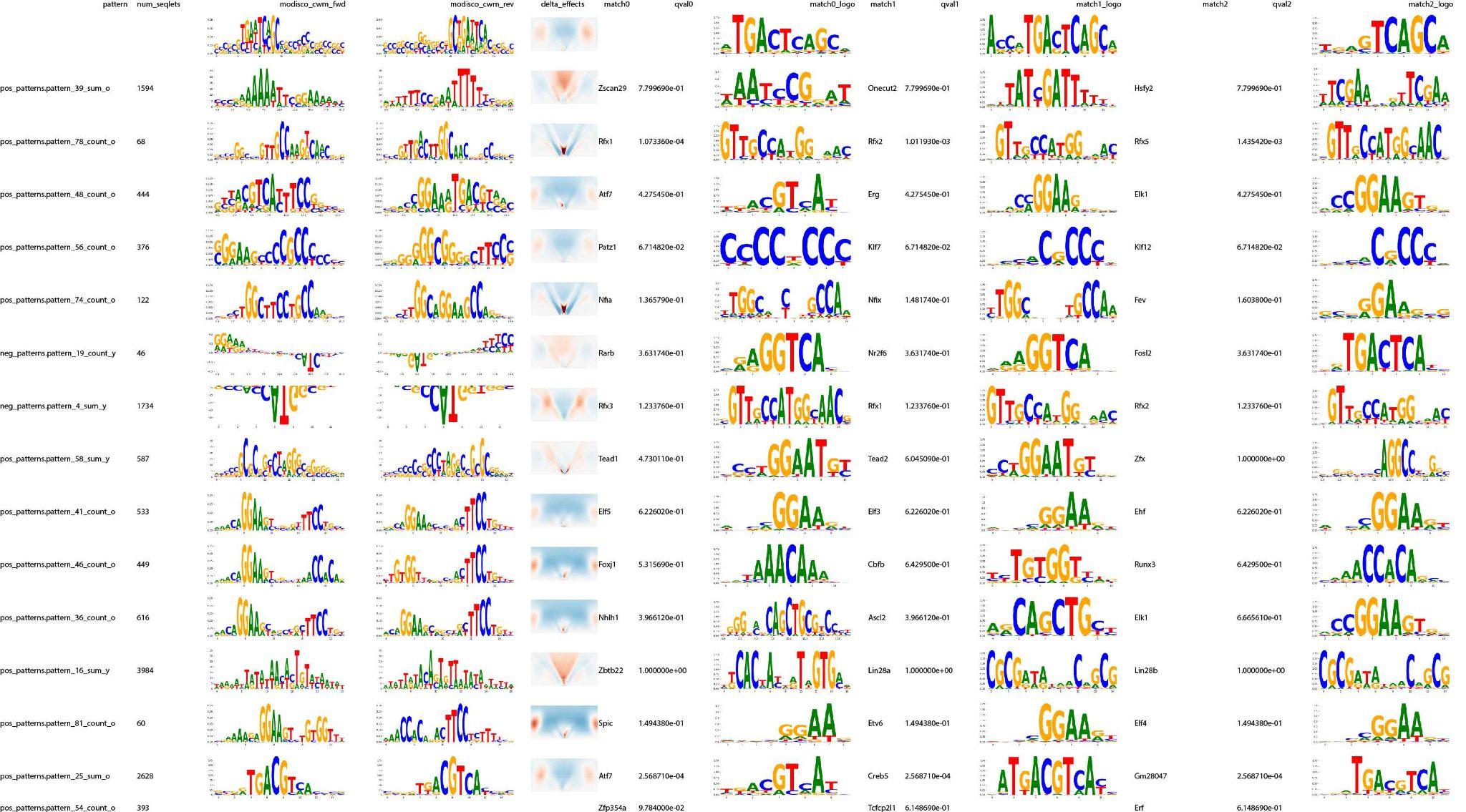

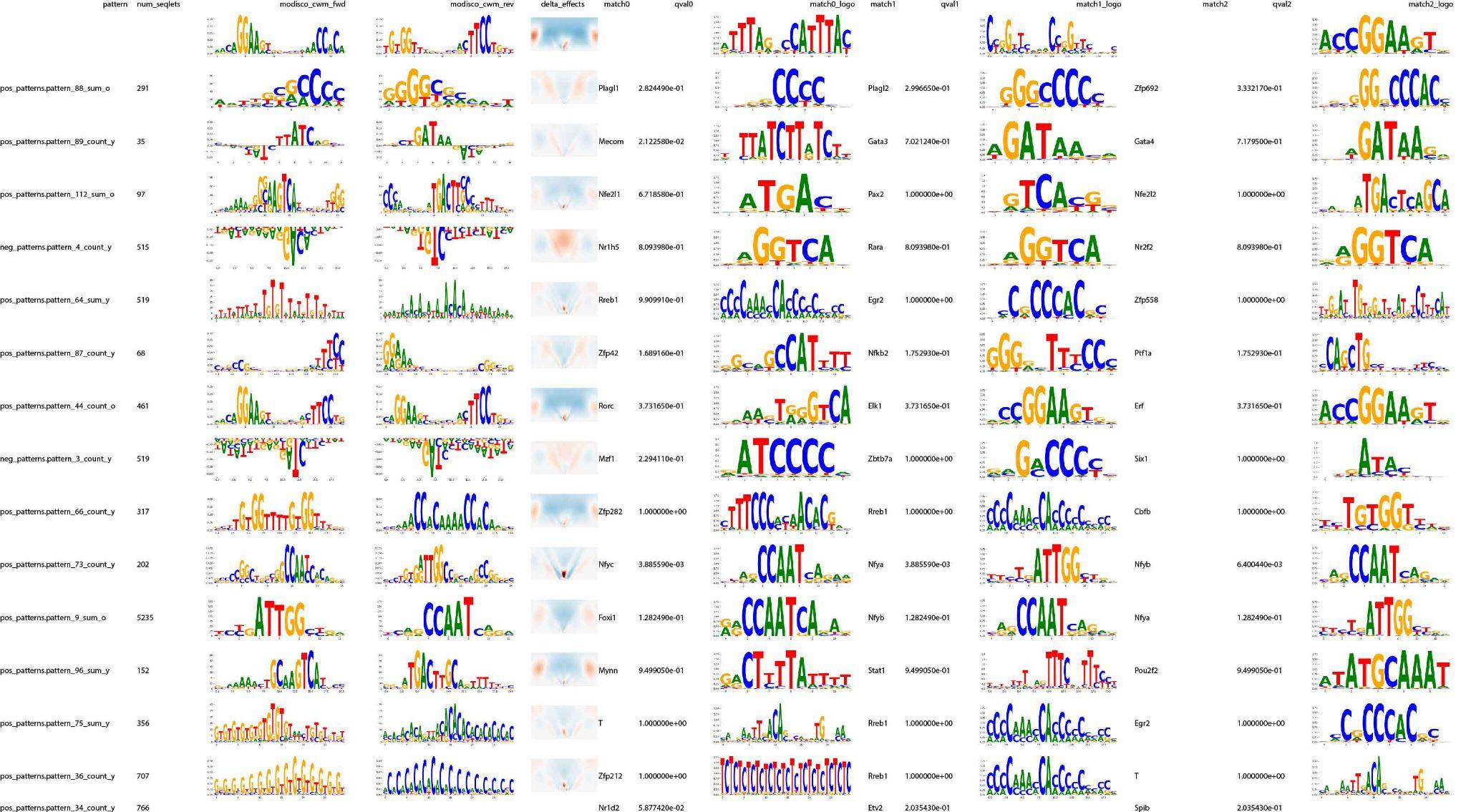

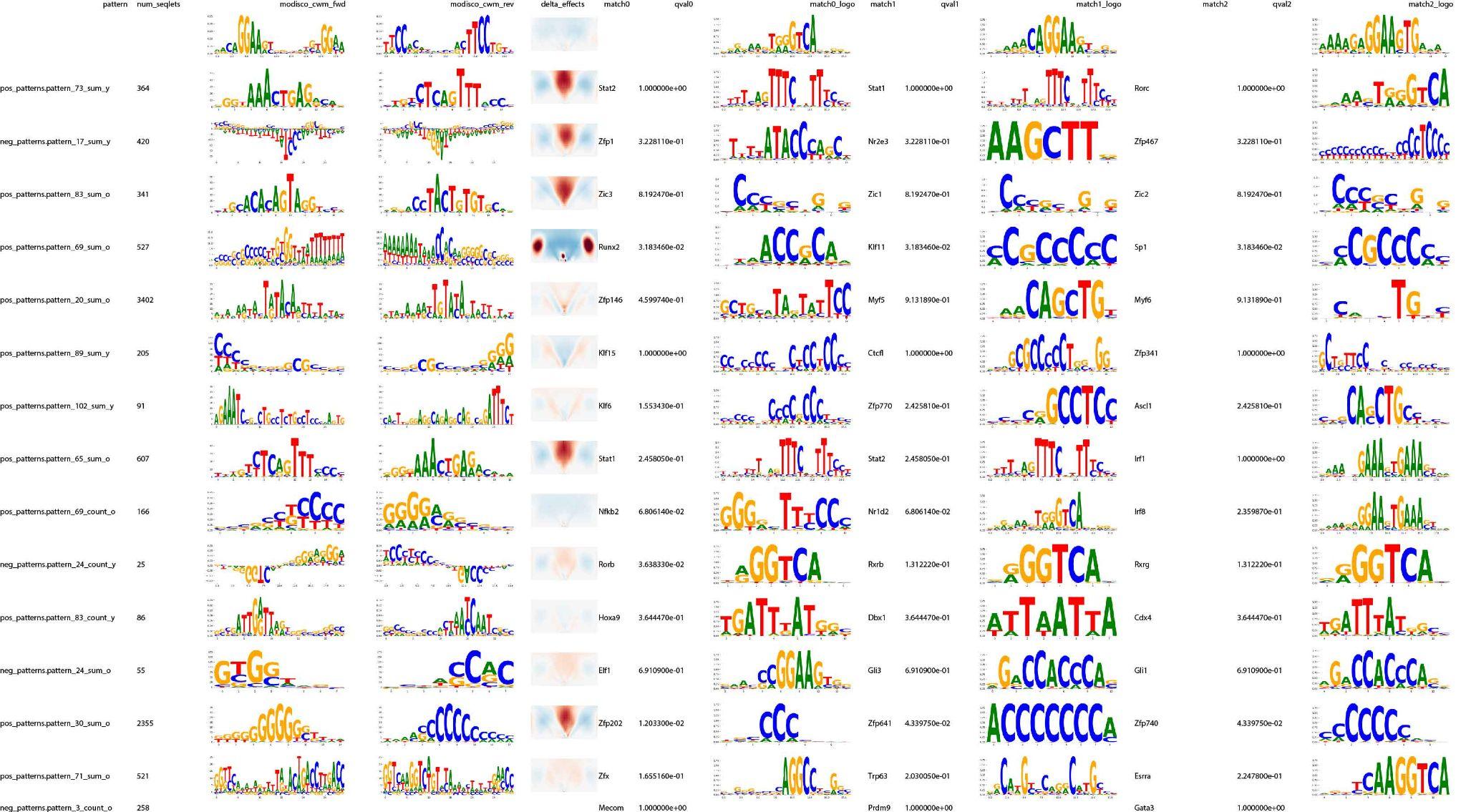

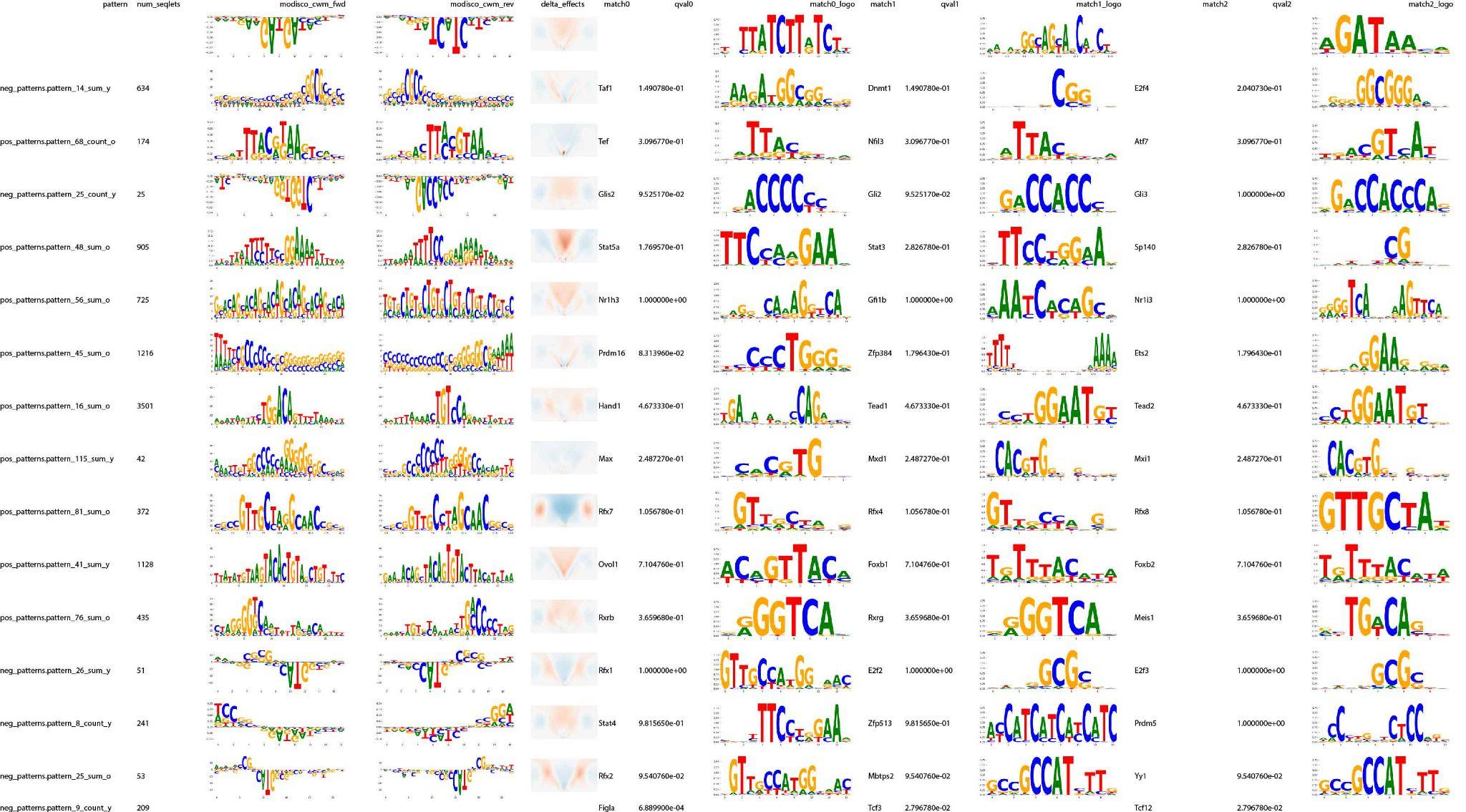

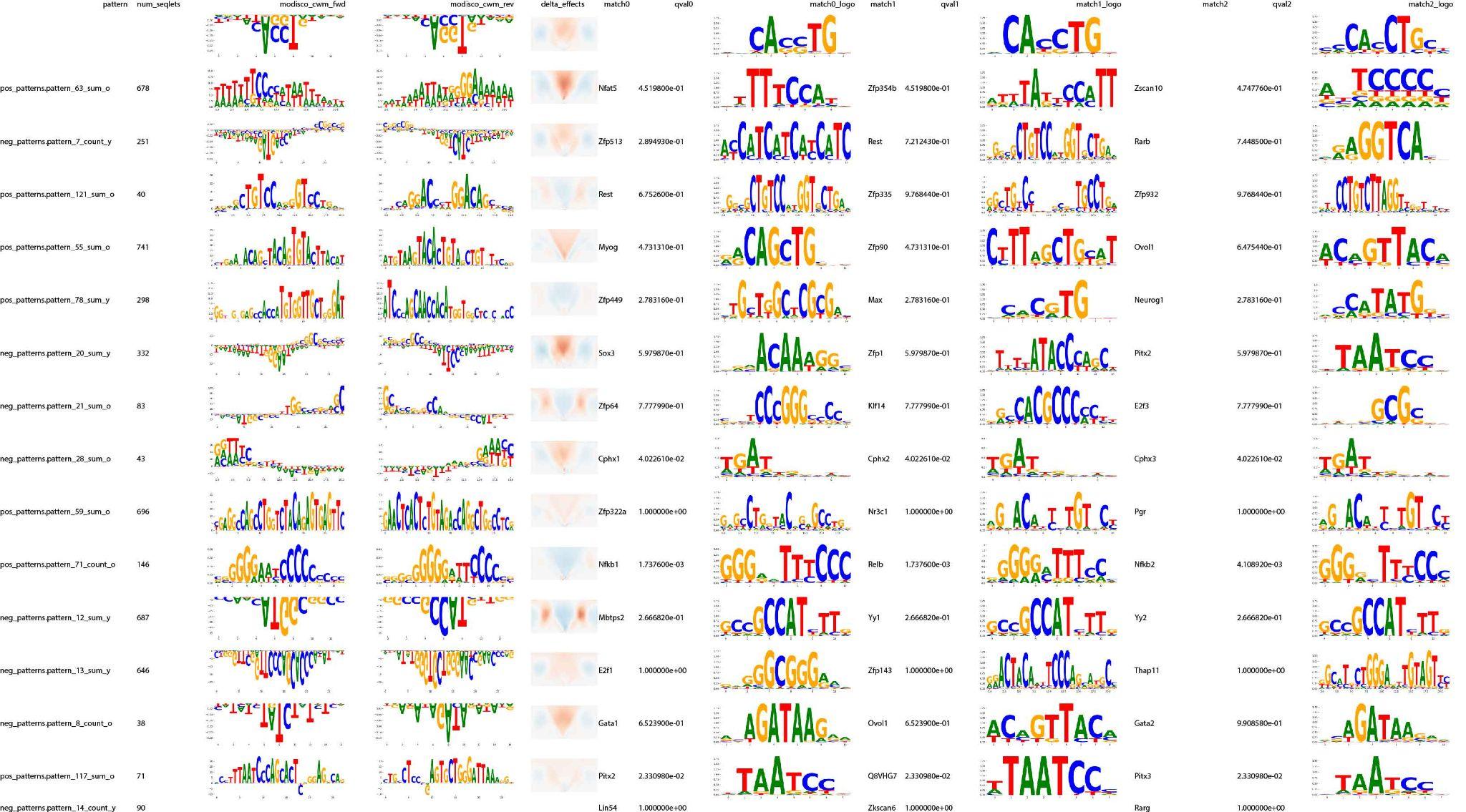

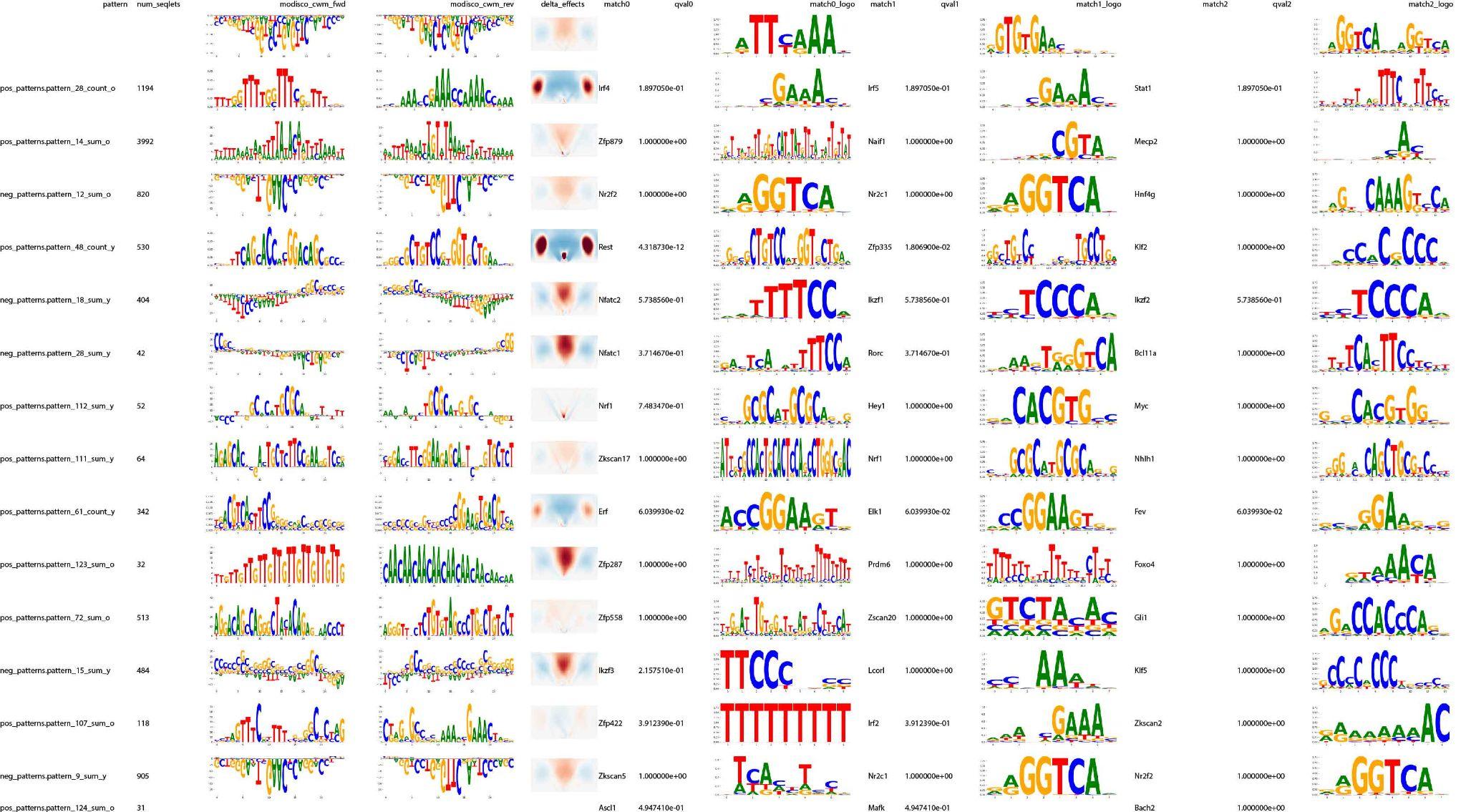

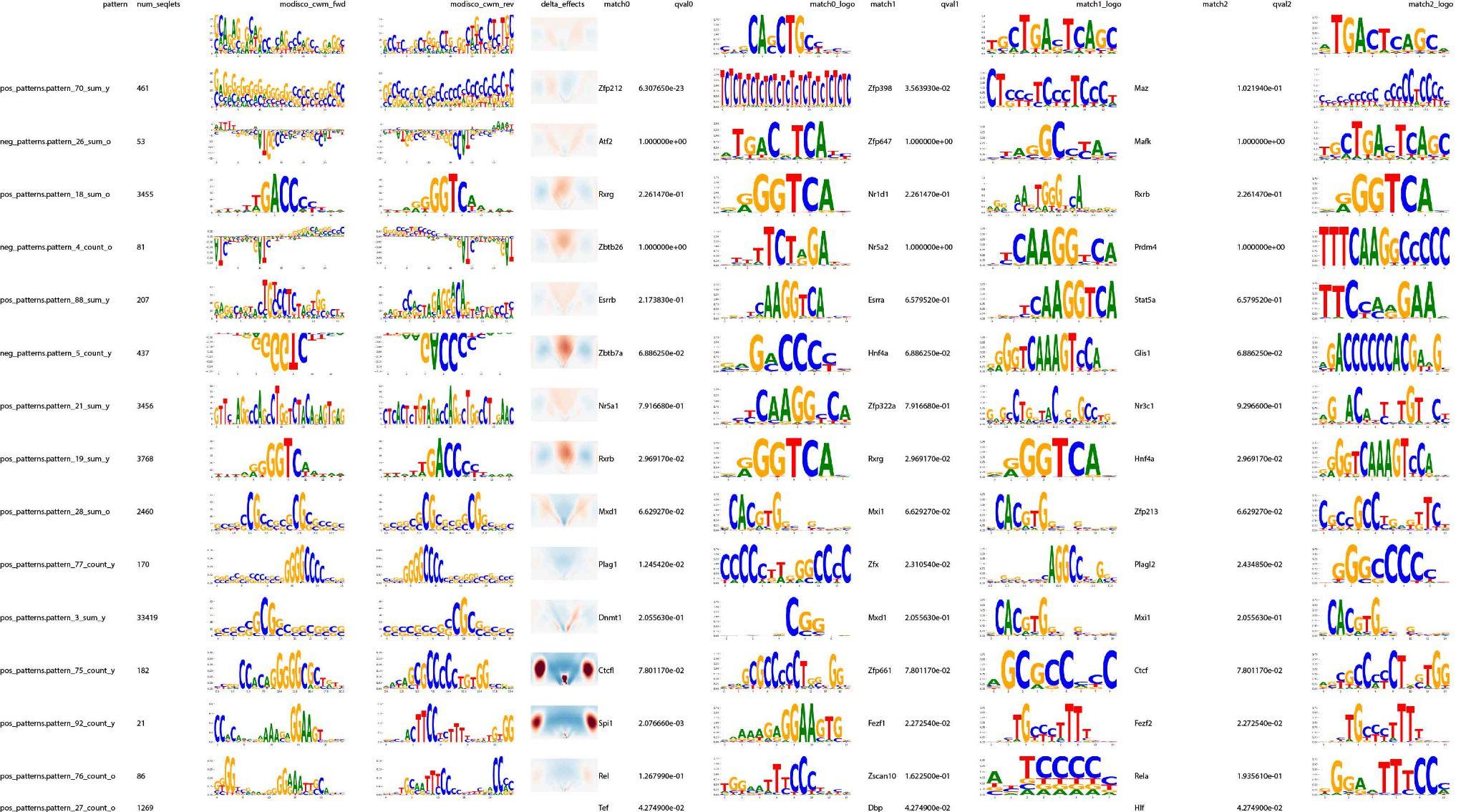

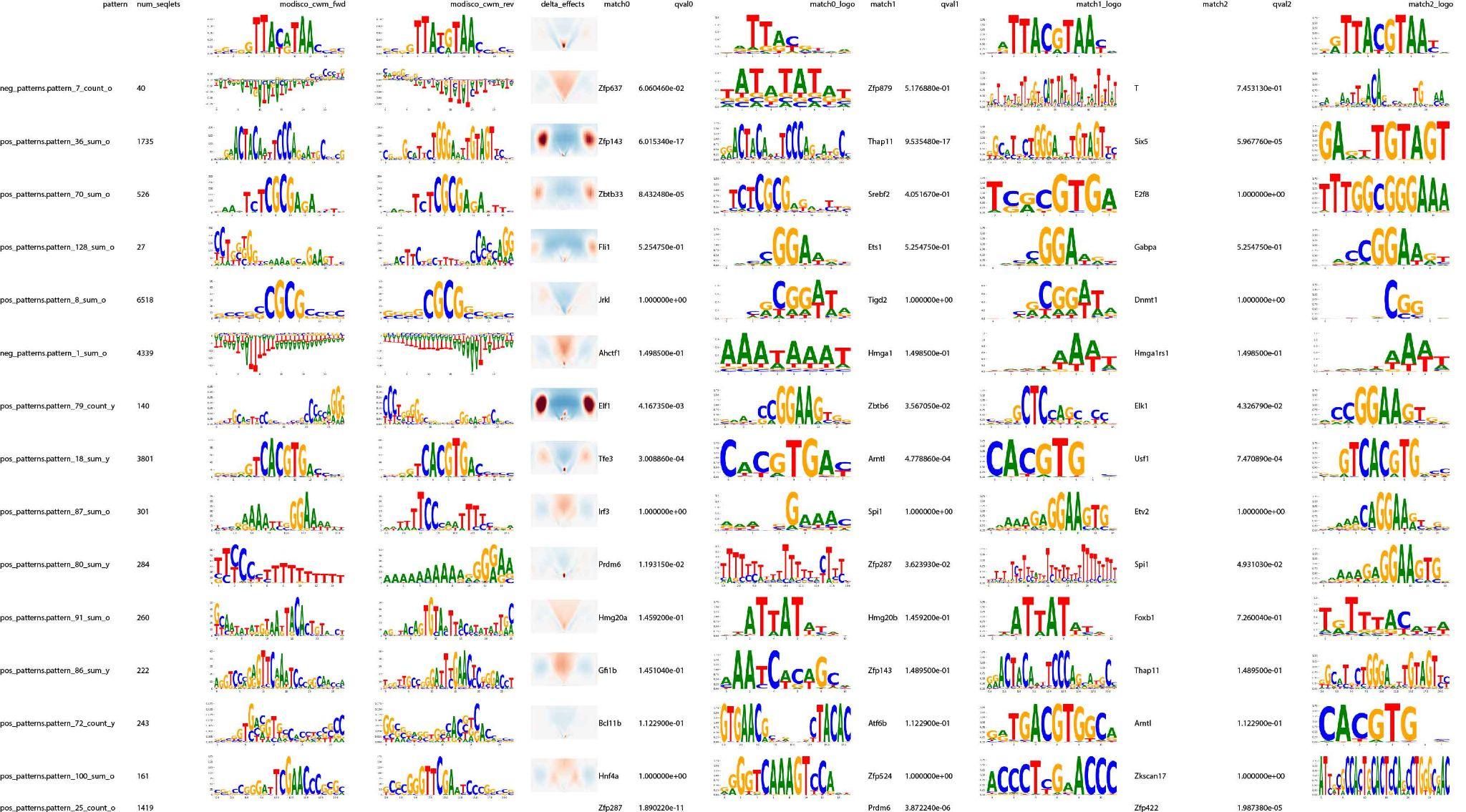

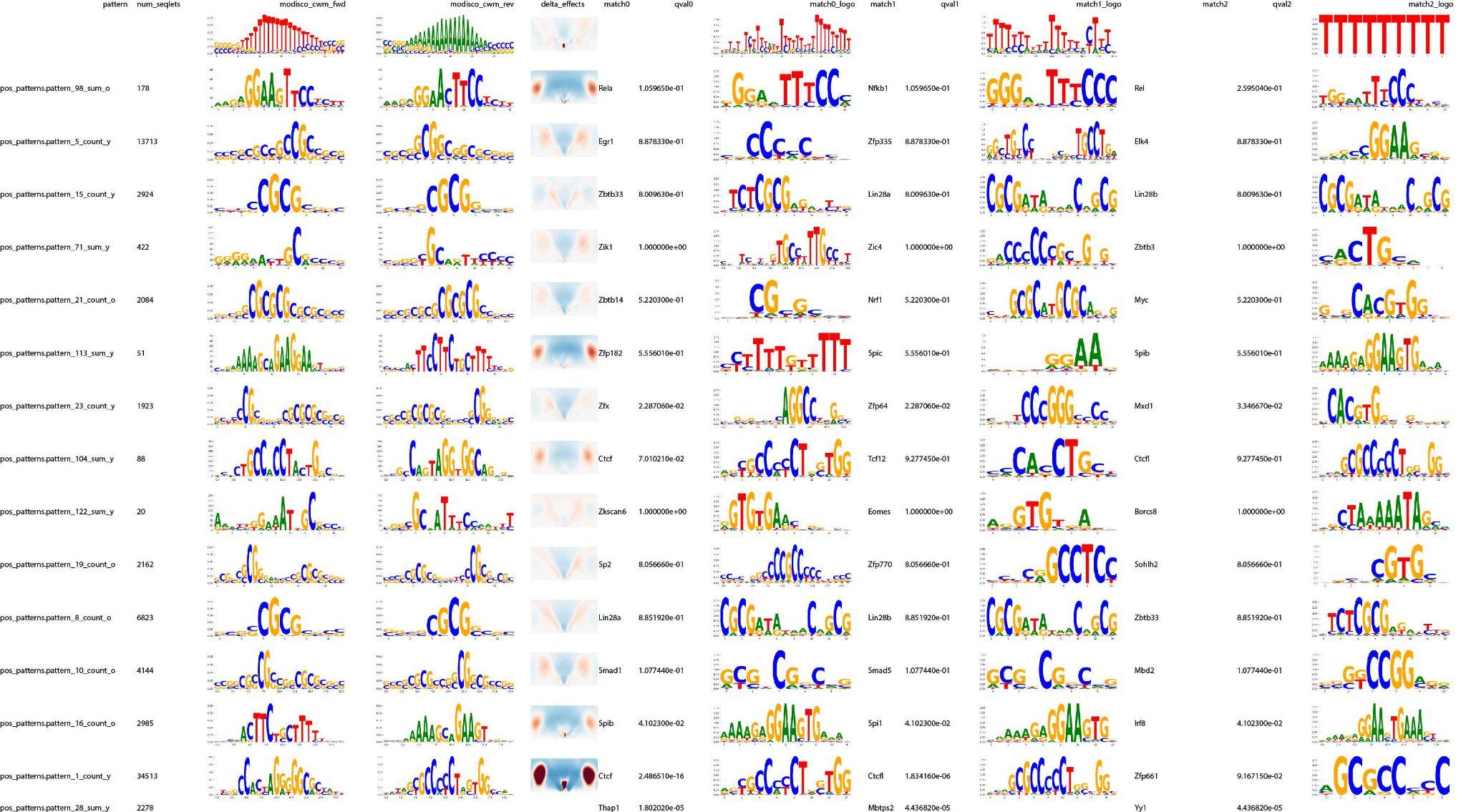


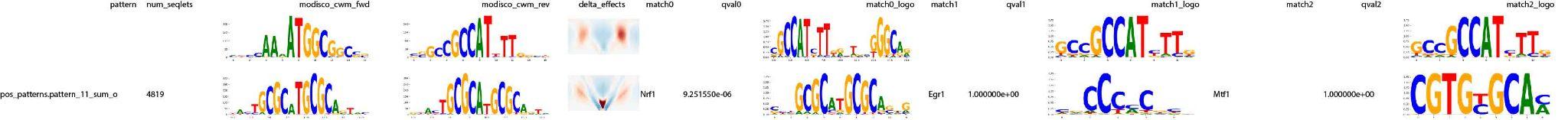


**Supplementary Data 3.** FACS gating strategy for sorting mouse HSCs containing results for each donor mouse.


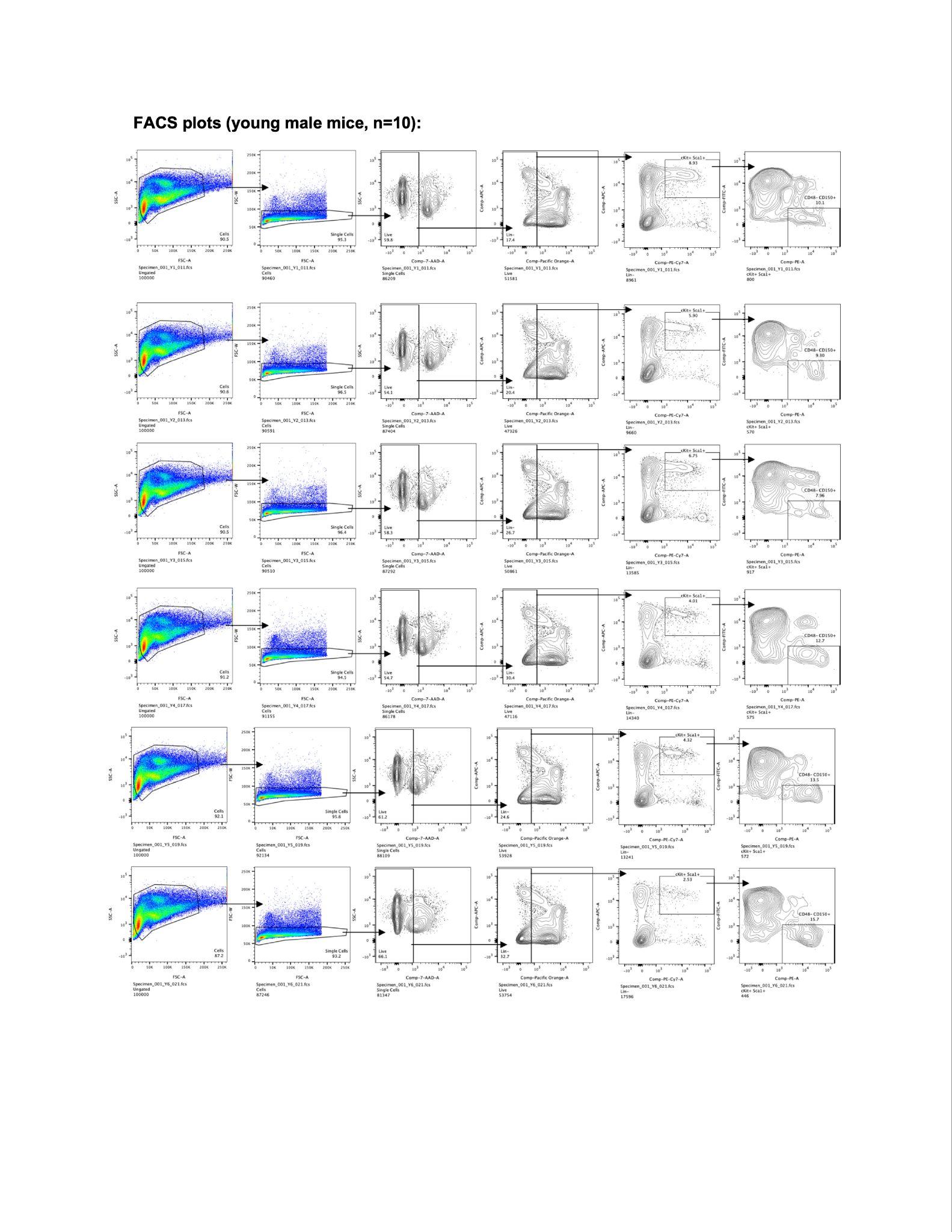


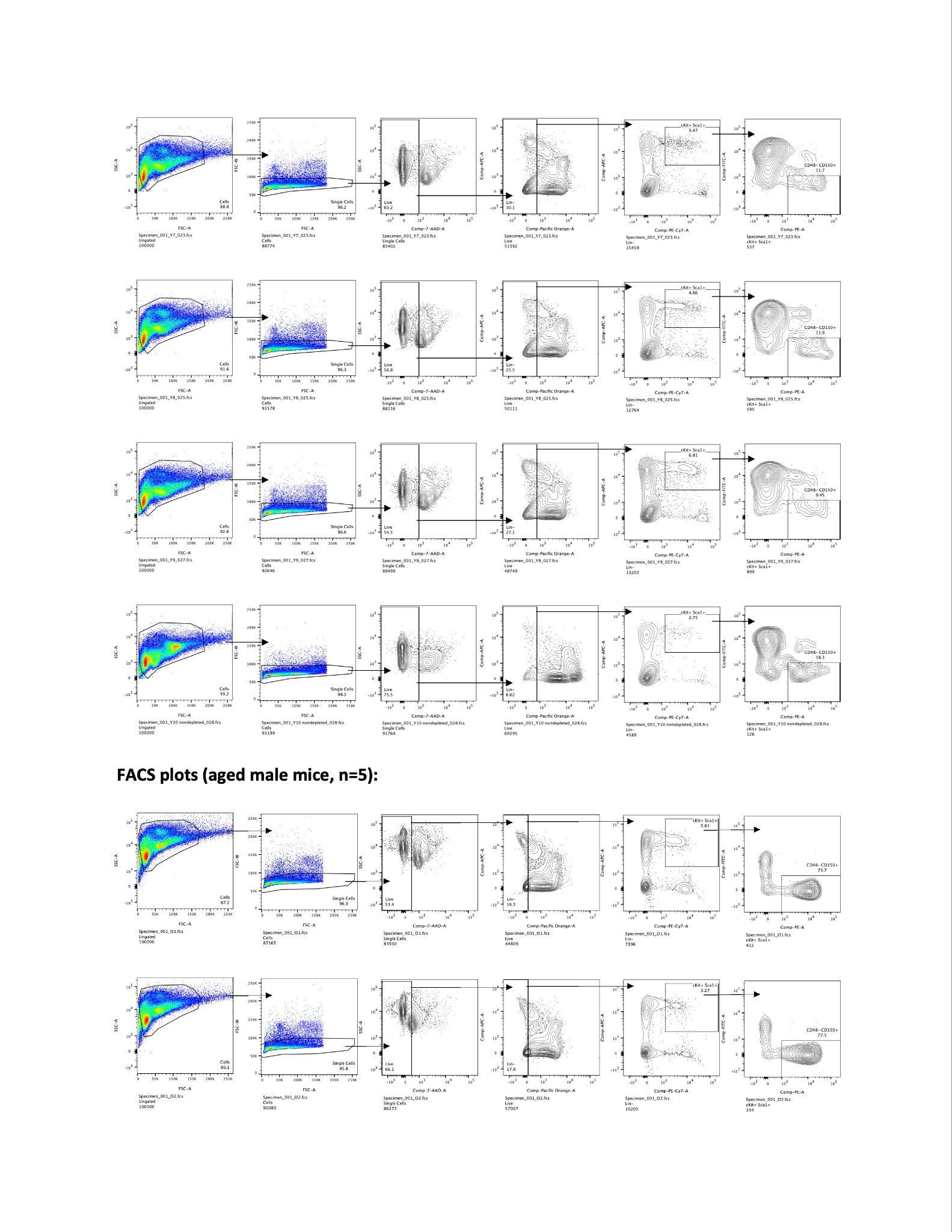


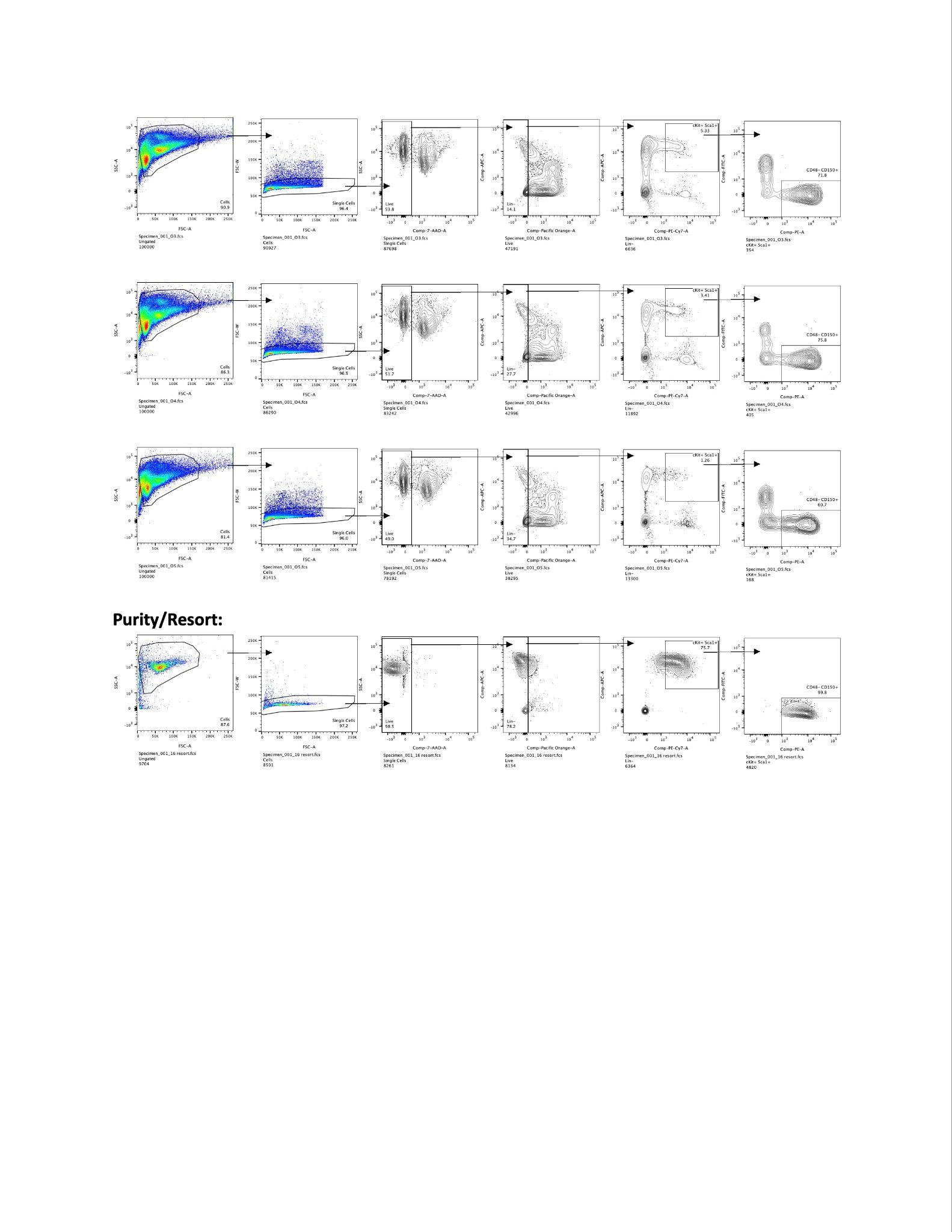

Supplement: Supplementary file 1 — This file contains notes, legends for tables and data. [file 41586_2024_8443_MOESM1_ESM.docx]
