# Supplementary material for: Multiscale footprints reveal the organization of cis-regulatory elements
Source: Nature. 2025 Jan 22;638(8051):779–86. doi: 10.1038/s41586-024-08443-4 (PMC11839466; doi:10.1038/s41586-024-08443-4)
Supplement: Supplementary file 2 — Reporting Summary [file 41586_2024_8443_MOESM2_ESM.pdf]

nature portfolio

Corresponding author(s): Jason Buenrostro

Last updated by author(s): Nov 7, 2024

Reporting Summary

Nature Portfolio wishes to improve the reproducibility of the work that we publish. This form provides structure for consistency and transparency in reporting. For further information on Nature Portfolio policies, see our Editorial Policies and the Editorial Policy Checklist.

Statistics

For all statistical analyses, confirm that the following items are present in the figure legend, table legend, main text, or Methods section.

n/a	Confirmed	
☐	☒	The exact sample size (*n*) for each experimental group/condition, given as a discrete number and unit of measurement
☐	☒	A statement on whether measurements were taken from distinct samples or whether the same sample was measured repeatedly
☐	☒	The statistical test(s) used AND whether they are one- or two-sided<br>*Only common tests should be described solely by name; describe more complex techniques in the Methods section.*
☒	☐	A description of all covariates tested
☐	☒	A description of any assumptions or corrections, such as tests of normality and adjustment for multiple comparisons
☐	☒	A full description of the statistical parameters including central tendency (e.g. means) or other basic estimates (e.g. regression coefficient) AND variation (e.g. standard deviation) or associated estimates of uncertainty (e.g. confidence intervals)
☐	☒	For null hypothesis testing, the test statistic (e.g. *F*, *t*, *r*) with confidence intervals, effect sizes, degrees of freedom and *P* value noted<br>*Give P values as exact values whenever suitable.*
☒	☐	For Bayesian analysis, information on the choice of priors and Markov chain Monte Carlo settings
☐	☒	For hierarchical and complex designs, identification of the appropriate level for tests and full reporting of outcomes
☐	☒	Estimates of effect sizes (e.g. Cohen's *d*, Pearson's *r*), indicating how they were calculated

Our web collection on statistics for biologists contains articles on many of the points above.

Software and code

Policy information about availability of computer code

Data collection	For flow cytometry, data analysis performed using BD FACS Diva (8.0.2) and FlowJo (10.8.2) software. Preprocessing of sequencing data was performed using CellRanger ARC (2.0.0) for 10x multi-ome data and the standard SHARE-seq pipeline for SHARE-seq data (https://github.com/masai1116/SHARE-seq-alignmentV2/).
Data analysis	An up-to-date GitHub repository containing all software related to this study and tutorials is available at https://github.com/buenrostrolab/PRINT and https://github.com/buenrostrolab/scPrinter. A static version of code used to generate the specific analyses in the manuscript is available at: https://figshare.com/s/e173a06b0c62c2f9dd1c.<br><br>Public packages and versions used in this manuscript and the accompanying software are as follows (for public repositories without version labeling, commit IDs are provided wherever available): MACS2 (2.2.9.1), chromVAR (1.24.0), cisTopic (0.3.0), Keras (2.15.0), TOBIAS (0.13.2), chromBPNet (v0.1.7), motifMatchr (1.24.0), Gviz (1.46.1), DeepLIFT (custom implementation included in scprinter), TFModisco (tfmodisco-lite v2.2.1), TOMTOM (meme suite v5.5.7), finemo (commit number 830d7f3), Palantir (1.0.0), ArchR (https://www.archrproject.com/), Seurat (5.0.3), SEACells (https://github.com/dpeerlab/SEACells/tree/main), Spectra (https://github.com/dpeerlab/spectra), sctransform (0.4.1), DESeq2 (1.42.1), AlphaFold3 (r2024.05.23), PyMol (2.6), ChimeraX (1.8)

For manuscripts utilizing custom algorithms or software that are central to the research but not yet described in published literature, software must be made available to editors and reviewers. We strongly encourage code deposition in a community repository (e.g. GitHub). See the Nature Portfolio guidelines for submitting code & software for further information.

Data

Policy information about availability of data

All manuscripts must include a data availability statement. This statement should provide the following information, where applicable:

- Accession codes, unique identifiers, or web links for publicly available datasets
- A description of any restrictions on data availability
- For clinical datasets or third party data, please ensure that the statement adheres to our policy

Raw and processed sequencing data from this study is available on Gene Expression Omnibus (GEO) with the accession number GSE216464.

Additional resources such as pre-trained machine learning models and pre-computed Tn5 bias tracks can be accessed on Zenodo at https://zenodo.org/record/7121027#.ZCbw4uzMI8N. Interactive visualization of human bone marrow and mouse aging datasets, as well as tracks of seq2PRINT footprint and TF predictions, are available through https://github.com/buenrostrolab/PRINT.

Reference genomes for Homo sapiens (hg38), Mus musculus (mm10), Drosophila melanogaster (dm6), Saccharomyces cerevisiae (sacCer3), Caenorhabditis elegans (ce11), Danio rerio (danRer11), and Pan troglodytes (panTro6) were accessed at https://hgdownload.soe.ucsc.edu/goldenPath/. SHARE-seq data from GM12878 was obtained from GEO GSE140203. Chemical mapping of nucleosomes was obtained from GSE97290. ChIP-exo data was from GSE151287. TF ChIP-seq was obtained from UniBind (https://unibind.uio.no/static/data/20220914/bulk_Robust/Homo_sapiens/damo_hg38_all_TFBS.tar.gz) and ENCODE (full list of accession numbers are in Supplementary Table 8). CTCF degron data was obtained from ENCODE (ENCSR328JGW, ENCSR260SWI). Dexamethasone-treatment data was obtained from ENCODE (full list of accession numbers are in Supplementary Table 8) and EMBL BioStudies (E-MTAB-9910, E-MTAB-9911, E-MTAB-9912). Interferon-treatment data was obtained from GEO GSE75306. All resources used in this study can be found in Supplementary Table 8.

Research involving human participants, their data, or biological material

Policy information about studies with human participants or human data. See also policy information about sex, gender (identity/presentation), and sexual orientation and race, ethnicity and racism.

Reporting on sex and gender	N/A
Reporting on race, ethnicity, or other socially relevant groupings	N/A
Population characteristics	N/A
Recruitment	N/A
Ethics oversight	N/A

Note that full information on the approval of the study protocol must also be provided in the manuscript.

Field-specific reporting

Please select the one below that is the best fit for your research. If you are not sure, read the appropriate sections before making your selection.

☒ Life sciences ☐ Behavioural & social sciences ☐ Ecological, evolutionary & environmental sciences

For a reference copy of the document with all sections, see nature.com/documents/nr-reporting-summary-flat.pdf

Life sciences study design

All studies must disclose on these points even when the disclosure is negative.

Sample size	Human bone marrow samples from 7 donors were included in the study. For mouse HSC aging experiments, 10 young mice and 5 old mice were included. In both cases, samples were pooled for single-cell sequencing and no comparisons were performed amongst donors. Sample size was chosen based on the number of organisms needed to yield sufficient numbers of the target cell populations for single-cell sequencing assays.
Data exclusions	Datasets generated in this study were filtered using standard quality thresholds commonly used for bulk ATAC-seq, scATAC-seq, SHARE-seq, and 10x Multiome data. All filters used are specified in the methods.
Replication	In vitro transposition of BAC DNA was performed in duplicate with R=0.97. All additional experiments were performed as analyses of large single-cell datasets, with comparisons performed internally among thousands of single cells or pseudobulk populations of cells as relevant. All summary statistics of these comparisons are included throughout the manuscript.
Randomization	All of our analyses were conducted on single-cell datasets and internal comparisons between populations were applied. No interventional studies on independent samples was conducted, so sample randomization is not relevant.

Blinding | All of our analyses were conducted on single-cell datasets and internal comparisons between populations were applied. Wherever possible, cell populations were applied without bias to population label as detailed in the Methods. However, it was not possible to blind investigators to individual datasets.

Reporting for specific materials, systems and methods

We require information from authors about some types of materials, experimental systems and methods used in many studies. Here, indicate whether each material, system or method listed is relevant to your study. If you are not sure if a list item applies to your research, read the appropriate section before selecting a response.

Materials & experimental systems

n/a	Involved in the study	
☐	☒	Antibodies
☐	☒	Eukaryotic cell lines
☒	☐	Palaeontology and archaeology
☐	☒	Animals and other organisms
☒	☐	Clinical data
☒	☐	Dual use research of concern
☒	☐	Plants

Methods

n/a	Involved in the study	
☒	☐	ChIP-seq
☐	☒	Flow cytometry
☒	☐	MRI-based neuroimaging

Antibodies

Antibodies used

Human: anti-human CD15 antibody (BioLegend, 301913).
Mouse: CD3 clone C145-2c11 (Biolegend, 100304; 1:100), CD4 clone GK15 (Biolegend, 100404; 1:400), CD5 clone 53-7.3 (eBioscience, 13-0051-85; 1:400), CD8 clone 53-6.7 (Biolegend, 100704; 1:400), CD19 clone 6D5 (Biolgend, 115504; 1:400), B220 clone RA3-6B2 (Biolegend, 103204; 1:200), GR1 (Ly6-G/Ly6-C) clone RB6-8C5 (eBioscience, 13-5931-82; 1:400), Mac1/CD11b clone M1/70 (Biolegend, 101204; 1:800), and Terr119 clone TERR-119 (Biolegend, 116204; 1:100), Sca1(Ly-6a/E) clone D7 (eBioscience, 25-5981-82; 1:200), cKit clone 2B8 (BD Pharmingen, 553356; 1:200), CD48 clone HM48-1 (Biolegend, 103403; 1:200), CD150 clone Tc15-12F12.2 (Biolegend, 115904; 1:200).

Validation

Validation was performed for antibodies as stated by the manufacturer:

BioLegend, 301913: by IF-FC
Biolegend, 100304: by IF-FC, IHC
Biolegend, 100404: by IF-FC
eBioscience, 13-0051-85: by IF-FC
Biolegend, 100704: by IF-FC
Biolegend, 115504: by IF-FC
Biolegend, 103204: by IF-FC, IHC
eBioscience, 13-5931-82: by IF-FC
Biolegend, 101204: by IF-FC, IHC, ICC
Biolegend, 116204: by IF-FC
eBioscience, 25-5981-82: by IF-FC
BD Pharmingen, 553356: by IF-FC
Biolegend, 103403: by IF-FC
Biolegend, 115904: by IF-FC

(IF-FC = immunofluorescence flow cytometry on relevant species and cell type; IHC = immunohistochemistry; ICC = immunocytochemistry)

Eukaryotic cell lines

Policy information about cell lines and Sex and Gender in Research

Cell line source(s) | HepG2 cells were obtained from ATCC (HB-8065).

Authentication | ATCC validated cell line identity via STR profiling prior to shipment.

Mycoplasma contamination | Cell lines were tested for mycoplasma by ATCC prior to shipment. Mycoplasma contamination is readily identifiable via ATAC-seq, and no evidence of contamination was observed.

Commonly misidentified lines (See ICLAC register) | No commonly misidentified lines were used in this study.

Animals and other research organisms

Policy information about studies involving animals; ARRIVE guidelines recommended for reporting animal research, and Sex and Gender in Research

Laboratory animals	C57BL6 mice were obtained from either Jackson Laboratory or the National Institute on Aging Aged Rodent Colony (Charles River Laboratory). Cells were harvested from young (11 week old) and old (24 month old) mice.
Wild animals	The study did not involve wild animals.
Reporting on sex	All mice used in this study are males.
Field-collected samples	The study did not involve collection of samples from the field.
Ethics oversight	Mouse experiments were approved and performed in compliance with Harvard University's Institutional Animal Care and Use Committee.

Note that full information on the approval of the study protocol must also be provided in the manuscript.

Plants

Seed stocks	*Report on the source of all seed stocks or other plant material used. If applicable, state the seed stock centre and catalogue number. If plant specimens were collected from the field, describe the collection location, date and sampling procedures.*
Novel plant genotypes	*Describe the methods by which all novel plant genotypes were produced. This includes those generated by transgenic approaches, gene editing, chemical/radiation-based mutagenesis and hybridization. For transgenic lines, describe the transformation method, the number of independent lines analyzed and the generation upon which experiments were performed. For gene-edited lines, describe the editor used, the endogenous sequence targeted for editing, the targeting guide RNA sequence (if applicable) and how the editor was applied.*
Authentication	*Describe any authentication procedures for each seed stock used or novel genotype generated. Describe any experiments used to assess the effect of a mutation and, where applicable, how potential secondary effects (e.g. second site T-DNA insertions, mosiacism, off-target gene editing) were examined.*

Flow Cytometry

Plots

Confirm that:

☒ The axis labels state the marker and fluorochrome used (e.g. CD4-FITC).

☒ The axis scales are clearly visible. Include numbers along axes only for bottom left plot of group (a 'group' is an analysis of identical markers).

☒ All plots are contour plots with outliers or pseudocolor plots.

☒ A numerical value for number of cells or percentage (with statistics) is provided.

Methodology

Sample preparation	For HSC isolation and flow cytometry. Cells from the bone marrow of long bones (2 femurs and 2 tibias per mouse) from young (n = 10; 11 weeks old) and aged (n = 5; 24 mo. old) male C57BL/6 mice were flushed with a 21-gauge needle into staining media (HBSS/2% fetal bovine serum), pelleted, and resuspended in ACK lysis buffer for 5 min on ice. Cells were then washed with staining media, filtered through a 40mm cell strainer, pelleted, and incubated with the following cocktail of rat anti-mouse, biotin conjugated lineage antibodies on ice for 30 min: CD3 clone C145-2c11 (Biolegend, 1000304; 1:100), CD4 clone GK15 (Biolegend, 1000404; 1:400), CD5 clone 53-7.3 (eBioscience, 13-0051-85; 1:400), CD8 clone 53-6.7 (Biolegend, 100704; 1:400), CD19 clone 6D5 (Biolgend, 115504; 1:400), B220 clone RA3-6B2 (Biolegend, 103204; 1:200), GR1 (Ly6-G/Ly6-C) clone RB6-8C5 (eBioscience, 13-5931-82; 1:400), Mac1/CD11b clone M1/70 (Biolegend, 101204; 1:800), and Terr119 clone TERR-119 (Biolegend, 116204; 1:100). Cells were then washed in staining media, with a small aliquot reserved for each sample to serve as a non-depleted control, and lineage depleted using sheep anti-rat Dynabeads (Invitrogen, 1135) on a magnet. Cells were washed, pelleted, and incubated with the following cocktail of anti-mouse antibodies on ice for 45 min. to identify hematopoietic stem cells (HSC): Pacific Orange Streptavidin (Invitrogen, S32365; 1:500), PE/Cy7 Sca1(Ly-6a/E) clone D7 (eBioscience, 25-5981-82; 1:200), APC cKit clone 2B8 (BD Pharmingen, 553356; 1:200), FITC CD48 clone HM48-1 (Biolegend, 103403; 1:200), and PE CD150 clone Tc15-12F12.2 (Biolegend, 115904; 1:200). Following incubation, cells were washed and resuspended in staining media, and 7-AAD (BD Pharmingen, 559925; 1:50) added immediately prior to flow cytometry.
Instrument	Cell sorting of HSCs (Live Lin- Sca1+ cKit+ CD48- CD150+) was performed on a BD FACS Aria II.
Software	Data analysis performed using BD FACS Diva and FlowJo software.

Cell population abundance	Frequency of HSCs in live cells range from around 0.2% (young mice) to 0.5% (old mice).
Gating strategy	The gating strategy used in this paper can be found in Extended Data Figures.

☒ Tick this box to confirm that a figure exemplifying the gating strategy is provided in the Supplementary Information.
